# Actinofuranones D-I from a Lichen-Associated Actinomycetes, *Streptomyces gramineus*, and Their Anti-Inflammatory Effects

**DOI:** 10.3390/molecules23092393

**Published:** 2018-09-18

**Authors:** Jian Ma, Bixuan Cao, Chengbin Liu, Peipei Guan, Yu Mu, Yi Jiang, Li Han, Xueshi Huang

**Affiliations:** 1 Institute of Microbial Pharmaceuticals, College of Life and Health Sciences, Northeastern University, Shenyang 110819, China; sherrie525358@126.com (J.M.); caobixuan1995@163.com (B.C.); guanpp@mail.neu.edu.cn (P.G.); muyu@mail.neu.edu.cn (Y.M.); huangxs@mail.neu.edu.cn (X.H.); 2 Yunnan Institute of Microbiology, School of Life Science, Yunnan University, Kunming 650091, China; liuchengbin17@163.com

**Keywords:** lichen-associated actinomycetes, actinofuranones, RAW 264.7 macrophages, anti-inflammatory

## Abstract

Six new metabolites, actinofuranones D-I (compounds **1**–**6**), were isolated together with three known compounds—JBIR-108 (**7**), E-975 (**8**), and E-492 (**9**)—from a fermentation broth of *Streptomyces gramineus* derived from the lichen *Leptogium trichophorum*. The structures of the new compounds **1**–**6** were established using comprehensive NMR spectroscopic data analysis, as well as UV, IR, and MS data. The anti-inflammatory activity of these isolated compounds were evaluated by examining their ability to inhibit nitric oxide (NO) production in LPS-stimulated RAW 264.7 macrophage cells. Compounds **4**, **5**, **8**, and **9** attenuated the production of NO due to the suppression of the expression of nitric oxide synthase (iNOS) in LPS-induced RAW 264.7 cells. Moreover, **4**, **5**, **8**, and **9** also inhibited LPS-induced release of proinflammatory cytokines interleukin-6 (IL-6) and tumor necrosis factor α (TNF-α).

## 1. Introduction

Lichens are symbiotic organisms, composed of fungi (mycobiont) and algae or cyanobacteria (photobiont or phycobiont). Like mycorrhizal fungi and fungal endophytes, they frequently host diverse bacterial communities as symbiotic niches [1]. The lichen-forming fungi and endolichenic fungi inhabiting the lichen thalli had been widely investigated as a source for discovering new bioactive natural products [2,3]. Recently, with discovery of some new secondary metabolites from them, lichen-associated actinomycetes have attracted extensive attention [4,5,6,7,8].

In our previous work, the diversity of cultivable actinomycetes associated with lichen symbiosis in samples collected from Yunnan Province, P. R. China were investigated. A total of 213 actinomycetes strains were isolated from 35 lichen samples and 16S rRNA gene sequence analysis of the isolates exhibited a high level of diversity among these strains [9]. Antimicrobical activities and biosynthetic potential studies of the isolated actinomycetes were also conducted in the research and the results showed the actinomycetes associated with lichen could be considered as a potential microbial resource for discovering novel bioactive natural products [9]. As a subsequent work, *Streptomyces gramineus* (YIM 130461), occurring in the lichen *Leptogium trichophorum*, collected from an evergreen broad-leaf forest in Benzilan, Diqing (Yunnan Province, China) was chosen to investigated its secondary metabolites. A small scale (100 mL) fermentation broth of *S. gramineus* showed diverse chemical constituents by HPTLC and HPLC-MS analysis. Chromatographic separation of an EtOAc extract of a large scale fermentation broth of *S. gramineus* led to the isolation of six new 2-hydroxy-2-(1-hydroxyethyl)-2,3-dihydro-3(2*H*)-furanones, named actinofuranones D-I (compounds **1**–**6**), as well as three known compounds: JBIR-108 (**7**) [10], E-975 (**8**) [11], and E-492 (**9**) [11].

Actinofuranones are relatively rare polyketide derivatives bearing a 2-hydroxy-2-methyl-2,3-dihydro-3(2*H*)- or 2-hydroxy-2-(1-hydroxyethyl)-2,3-dihydro-3(2*H*)-furanone skeleton, which has a hemiketal at C-2 and an unsaturated alkyl chain with 13–19 carbons at C-5. To date, only a few examples of analogues derived from actinomycetes were reported, including JBIR-108, E-975, E-492, E-837, actinofuranones A and B from *Streptomyces* sp. [10,11,12], actinofuranone C from *Amycolatopsis* sp. [13], and linfuranone A from *Microbispora* sp. [14]. In addition, some structurally related compounds were previously found in other sources, such as siphonarienfuranones isolated from the marine mollusk *Siphonaria* sp. [15,16,17], aglajne-2 from the opisthobranch mollusc *Bulla striata* [18], AS-183 from *Scedosporium* sp. fungi [19], and the aurafurons A and B from the myxobacteria *Stigmatella aurantiaca* and *Archangium gephyra* [20]. The total synthesis of aurafuron A and JBIR-108 have been achieved [10,21]. It is noteworthy that all of these natural products with a 2-hydroxy-2-methyl-2,3-dihydro-3(2*H*)-furanone skeleton mentioned above were obtained as a mixture of C-2 isomers with a 1:1 ratio. However, actinofuranone JBIR-108 with a 2-hydroxy-2-(1-hydroxyethyl)-2,3-dihydro-3(2*H*)-franone scaffold was isolated as a mixture of four diastereomers due to chiral carbons at C-1 and C-2. Till now, only three actinofuranones, including JBIR-108, E-975, E-492 with 2-(1-hydroxyethyl)-substituents have been found.

The structures of compounds **1**–**9** were elucidated using comprehensive NMR spectroscopic data analysis. Meanwhile, as part of our continuous search for the new anti-inflammatory compounds from lichen-associated actinomycetes, we investigated the anti-inflammatory activity of the isolates by examining their ability to inhibit production of nitric oxide (NO), interleukin-6 (IL-6) and tumor necrosis factor α (TNF-α), and the expression of inducible nitric oxide synthase (iNOS) in LPS-stimulated RAW 264.7 macrophage cells. Herein, details of the isolation, structure elucidation, anti-inflammatory effects of these derivatives are reported.

## 2. Results and Discussion

### 2.1. Structural Elucidation of Actinofuranones D-I

**Figure 1:**
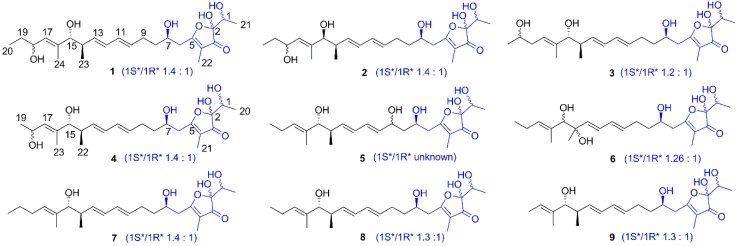
Structures of compounds **1**–**9**.

A large-scale (70 L) fermentation broth of *S. gramineus* was centrifuged and the supernatant was extracted with ethyl acetate. Then, the dried extract was fractionated by sequential chromatography over Sephadex LH-20, silica gel, and octadecylsilyl-bonded silica (ODS) to yield nine actinofuranones **1**–**9** (Figure 1), including the six new actinofuranone analogues **1**–**6**. All of them were isolated as a mixture of C-1 and C-2 diastereomers. Here the major components were employed for the structural establishment.

**Figure 2:**
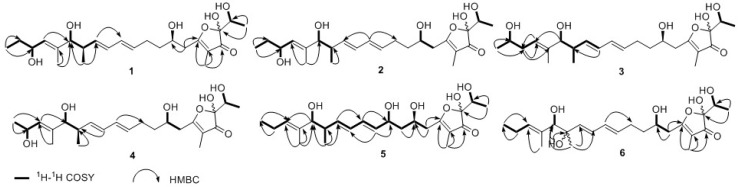
^1^H-^1^H COSY and key HMBC correlations for compounds **1**–**6**.

Compound **1** was isolated as a colorless amorphous substance. Its molecular formula of C_24_H_38_O_7_ was confirmed by HRESIMS (*m/z* 461.2573 [M + Na]^+^), which indicated six degrees of unsaturation. The presence of hydroxyl (3357 cm^−1^), carbonyl (1693 cm^−1^) and double bonds (1645, 1613 cm^−1^) functionalities could be obtained from its IR spectrum. The ^1^H-NMR (DMSO-*d*_6_) spectrum of **1** (Table 1 and Supplementary Material) presented four sets of signals of four isomers. The signals of major isomer revealed that the presence of five olefinic protons (*δ*_H_ 5.97, 5.94, 5.61, 5.55, 5.17), four oxygenated methine protons (*δ*_H_ 4.10, 3.81, 3.69, 3.50), a methine proton (*δ*_H_ 2.19), four methylenes (*δ*_H_ 2.58; 2.17, 2.07; 1.60, 1.46; 1.42, 1.30), five methyl (*δ*_H_ 1.53, 1.52, 1.13, 0.79, 0.78), in addition to five active protons (*δ*_H_ 7.25, 4.70, 4.67, 4.61, 4.45). The ^13^C NMR and HSQC spectra interpreted the carbons corresponding with ^1^H NMR spectrum, as well as five quaternary carbons (*δ*_C_ 202.9, 185.4, 136.7, 109.8, 104.2). The ^1^H NMR and ^13^C NMR data of **1** showed typical characteristic of actinofuranone analogues and were very similar to those of 1S*-JBIR-108 (**7**) [10], which was also obtained from the same strain *S. gramineus* as an isomer mixture (major 1S*-isomer). Herein, we identified the structure of **1** through comparing NMR data in DMSO-*d*_6_ and methanol-*d*_4_ with those in the literature (methanol-*d*_4_). Compared with NMR data between major isomers of **1** and 1S*-**7**, the only difference was an extra hydroxyl located at the unsaturated alkyl chain in **1**. COSY correlations from H-18 (*δ*_H_ 4.10) to H-17 (*δ*_H_ 5.17), H-19 (*δ*_H_ 1.42, 1.30), and 18-OH (*δ*_H_ 4.45) confirmed the position of the additional hydroxy group at C-18 (Figure 2). The 10*E* and 12*E* configuration were deduced from the coupling constants of H-10 (*δ*_H_ 5.55, dd, *J* = 14.2, 6.8 Hz) and H-13 (*δ*_H_ 5.61, dd, *J* = 14.5, 7.3 Hz). NOE (in MeOH-*d*_4_) correlations from H-15 (*δ*_H_ 3.65) to H-17 (*δ*_H_ 5.30), from H-18 (*δ*_H_ 4.27) to H-24 (*δ*_H_ 1.65) elucidated the 16*E* configuration. The identical ^13^C NMR data (both in DMSO-*d*_6_ and MeOH-*d*_4_) of C-1 to C-15 and C-21 to C-24 of the major isomer in **1** with 1S*-**7** revealed that it possessed the same relative configuration at C-1, C-7, C-14, and C-15 as 1S*-7. The coupling constant ^3^*J*_H-14/H-15_ = 8.5 Hz (in MeOH-*d*_4_) further confirmed the configuration of H-14 and H-15. Therefore, the structure of the major isomer in **1** was elucidated as 1S*-actinofuranone D as shown in Figure 1. By the same deduction above, the minor isomer in **1** was identified as 1R*-actinofuranone D.

**Figure 3:**
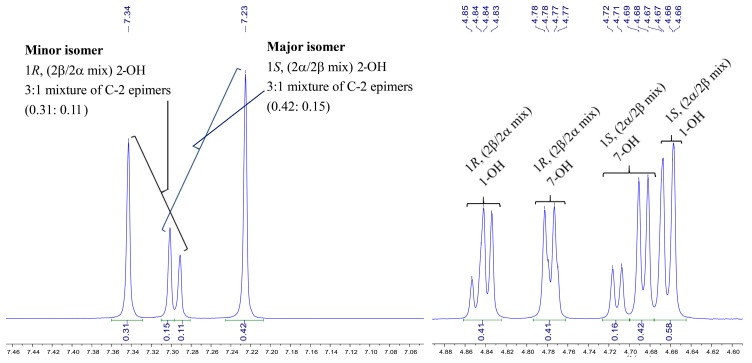
The ratio of the isomers mixture of **7**.

JBIR-108 was firstly isolated from *S. gramineus* IR087Pi-4 as a mixture of two diastereomers at C-1 and its planar structure and absolute configuration were determined by spectral analysis and total synthesis (1S*-/1R*-isomer, 5:2) [10]. The NMR data of JBIR-108 was measured in MeOH-*d*_4_ without presenting any active protons signals in the literature. They could not get the relative content of all isomers from the characteristic protons since most ^1^H-NMR signals of isomers were same. However, hydroxyl groups of isomers, especially the hydroxyl of hemiacetal group, presented individual ^1^H-NMR signals in the DMSO-*d*_6_ (Figure 3). Since the hemiacetal formation was mainly influenced by the steric hindrance of C-1 substitute, the ratio of epimers should be same (1*S*α/1*S*β = 1*R*β/1*R*α). Thus, we could deduce that **7** is a 1.4:1 mixture of diastereomers at C-1, actually. Even if it exhibited a 3:1 mixture of C-2 epimers for each C-1 diastereomer (*R*- or *S*-isomers), we could not give an absolute content for those 1*S*β, 1*S*α, 1*R*α or 1*R*β isomers (Figure 3). Approximately the same ratio of isomer was also deduced from the individual ^1^H-NMR signals of C-1 and C-7 hydroxyls in Figure 1.

The molecular formula of compound **2** was established to be C_24_H_38_O_7_ on the basis of HRESIMS (*m/z* 461.2651 [M + Na]^+^). The UV, IR, ^1^H-NMR, and ^13^C-NMR data indicated that **2** was also an actinofuranone analogue. Further inspection of the HSQC, COSY, and HMBC spectra elucidated **2** possessed the same planar structure as **1** (Figure 2). Compound **2** had identical ^13^C-NMR data as **1**, except for C-13 (∆*δ*_C_ = +0.9 ppm), C-15 (∆*δ*_C_ = +1.0 ppm), C-17 (∆*δ*_C_ = +1.2 ppm), and C-24 (∆*δ*_C_ = −1.3 ppm) which hinted **2** was a C-15 isomer of **1**. The smaller coupling constants of H-14/H-15 in **2** compared with those of **1** (^3^*J*_H_-_14_/_H-15_ = 7.0 Hz vs. 8.5 Hz in MeOH-*d*_4_; ^3^*J*_H_-_14_/_H-15_ = 5.6 Hz vs. 7.8 Hz in DMSO-*d*_6_) confirmed the different configuration of C-15 between them. The double bonds were further confirmed as 10*E*, 12*E*, 16*E* by analysis the coupling constants and NOE correlations. Therefore, the structure of **2** was identified as that in Figure 1 and named as actinofuranone E.

Compound **3** was obtained as a colorless amorphous powder. Its ^13^C-NMR data and HRESIMS data (*m/z* 461.2654 [M + Na]^+^) suggested that **3** has the same molecular formula as that of **1** and **2** (C_24_H_38_O_7_). The structure of **3** was almost identical to **1** by comparison with ^1^H- and ^13^C-NMR data (Table 2 and Supplementary Material) of **1**. However, ^1^H-^1^H COSY correlation between the terminal methyl protons H_3_-20 (*δ*_H_ 1.00) and oxygenated methine proton H-19 (*δ*_H_ 3.58) indicated that the hydroxyl group was located at C-19 in **3** instead of C-18 in **1** (Figure 2). The assignment of the hydroxyl was further confirmed by the clear HMBC correlations between H_3_-20 (*δ*_H_ 1.00) and C-19 (*δ*_C_ 66.6), C-18 (*δ*_C_ 37.8), between H-18 (*δ*_H_ 1.98) and C-16 (*δ*_C_ 138.2), C-17 (*δ*_C_ 123.1) (Figure 2). The identical ^13^C-NMR data of C-1 to C-15 and C-21 to C-24 in **3** with those of **1** deduced the relative configurations of chiral carbons in **3** except for C-19. Thus, the structure of **3** was elucidated and named as actinofuranone F.

As displayed by ^13^C-NMR data and [M + Na]^+^ ion at *m/z* 447.2363 in HRESIMS, compound **4** had the molecular formula of C_23_H_36_O_7_. The difference in mass compared to **1** owed to a missing CH_2_ group. Furthermore, the ^1^H-NMR and ^13^C-NMR data of **4** (Table 2 and Supplementary Material) were very similar to those of **1** except for the terminal methyl instead of ethyl in the C-5 alkenyl side. Cross-peaks between H-18 (*δ*_H_ 4.35) and H-17 (*δ*_H_ 5.25) and terminal methyl protons H_3_-19 (*δ*_H_ 1.05) observed in ^1^H-^1^H COSY spectra confirmed the absence of methylene at the end of C-5 alkenyl chain (Figure 2). The identical ^13^C-NMR data of C-1 to C-15 in **4** with those of **1** suggested the relative configurations of **4** and the stereochemistry of C-18 was undetermined.

Compound **5** was isolated as a colorless amorphous powder with molecular formula of C_23_H_36_O_7_ assigned from HRESIMS (*m/z* 447.2374 [M + Na]^+^). Obviously, **5** was a geometric isomer of **4**. Compared with **4** the C-18 hydroxyl in C-5 alkenyl chain of **5** migrated on the basis of analysis of NMR data of **4** and **5**. ^1^H-^1^H COSY correlations between H-6/H-7/H-8/H-9/H-10/H-11/H-12/H-13/H-14/H-15 and the HMBC interactions between H-11 (*δ*_H_ 6.10), H-10 (*δ*_H_ 5.54) and C-9 (*δ*_C_ 69.2) indicated the migrated hydroxyl was located at C-9 (Figure 2). The coupling constants of H-10 (*δ*_H_ 5.54, dd, *J* = 15.1, 6.2 Hz), H-13 (*δ*_H_ 5.67, dd, *J* = 15.3, 7.4 Hz) indicated the 10*E* and 12*E* configurations. NOE interactions observed from H-17 to H-15, H-18, H-19, H-22, from H-23 to H-14, H-15, H-18, H-22 determined the 16*E*. The almost identical ^13^C-NMR data with the total synthesis JBIR-108 (**7**) (Table 3 and Supplementary Material) combining with the biosynthesis procedure [11], the configuration of **5** was determined except for C-9.

The UV, IR, ^1^H-NMR, and ^13^C-NMR data indicated **6** was closely related to **4** and has the molecular formula of C_23_H_36_O_7_ based on the ion peak at *m/z* 447.2337 [M+Na]^+^ in its HRESIMS. Comparison of NMR data of **6** (Table 3 and Supplementary Material) with those of **4** showed that the substituted position of C-18 hydroxyl in **4** changed. Moreover, one oxygenated quaternary carbon and a methylene appeared in **6** instead of two methines of **4**. The singlet methyl at C-14 in **6** indicated the migrated hydroxyl located at C-14, which was confirmed by the HMBC correlations from H-22 to C-13, C-14, C-15, from H-13 to C-12, C-14 (Figure 2). Thus, the structure of **6** was determined, and named actinofuranone I. The ratios of the major isomers in **1**–**4**, and **6** could be approximately determined through analysis of ^1^H-NMR data of 2-OH and 7-OH (Figure 1), even though the 1-OH of those isomers did not present an ideally individual ^1^H-NMR signal.

### 2.2. Cell Viability and Effects of Compounds on the Production of NO in LPS-Induced RAW 264.7 Cells

**Figure 4:**
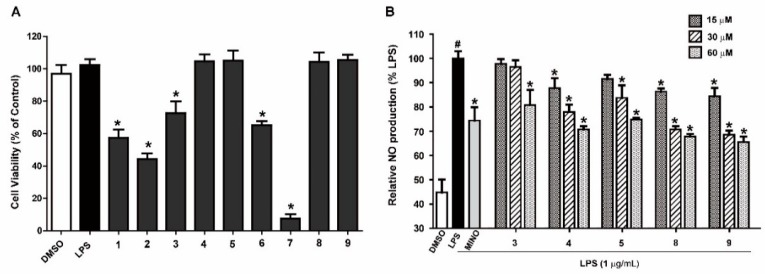
Cell viability of **1**–**9** against RAW 264.7 cells (**A**). Effect of **3**–**5**, **8** and **9** on the inhibition of LPS-induced NO in RAW 264.7 cells (**B**). Cells were incubated with compounds or minocycline (MINO, 25 μM) for 2 h then stimulated with or without LPS (1 μg/mL) for 24 h. Cell viability was evaluated by MTT assay. The NO production in the medium was measured using Griess agent. All conditions were run in triplicate, and data show mean ± SD values. ^#^, *p* < 0.05 was compared to control. *, *p* < 0.05 compared to LPS.

Nitric oxide (NO) produced by activated RAW 264.7 macrophages plays an important role in inflammation diseases [22]. Excess production of NO have been reported to be involved in inflammatory disorders [23]. In the present study, the anti-inflammatory activity of isolated compounds were investigated by evaluating their effects on production of NO in LPS-induced RAW 264.7 cells. In order to exclude influence of the cytotoxic of compounds **1**–**9** on the anti-inflammatory evaluation, the viability of RAW 264.7 cells with test compounds treatment was carried out by an MTT assay. The result showed that cell growth inhibitory rate of compounds **1**, **2**, **6**, and **7** were more than 40% at 60 μM and others had no obvious effect on cell viability at the test concentration (Figure 4A). Hence, the compounds **3**–**5**, **8**, and **9** were selected for the anti-inflammatory activity evaluation process. As displayed in Figure 4B, compounds **4**, **5**, **8**, and **9** significantly inhibited the production of LPS-induced NO in a dose-dependent manner at the varying concentration (0, 15, 30, and 60 μM). However, compound **3** have no inhibition effect on NO production in LPS-stimulated RAW 264.7 cells which were treated at the low concentration.

### 2.3. Compounds ***4***, ***5***, ***8***, and ***9*** Attenuated LPS-Induced iNOS Expression in RAW 264.7 Cells

**Figure 5:**
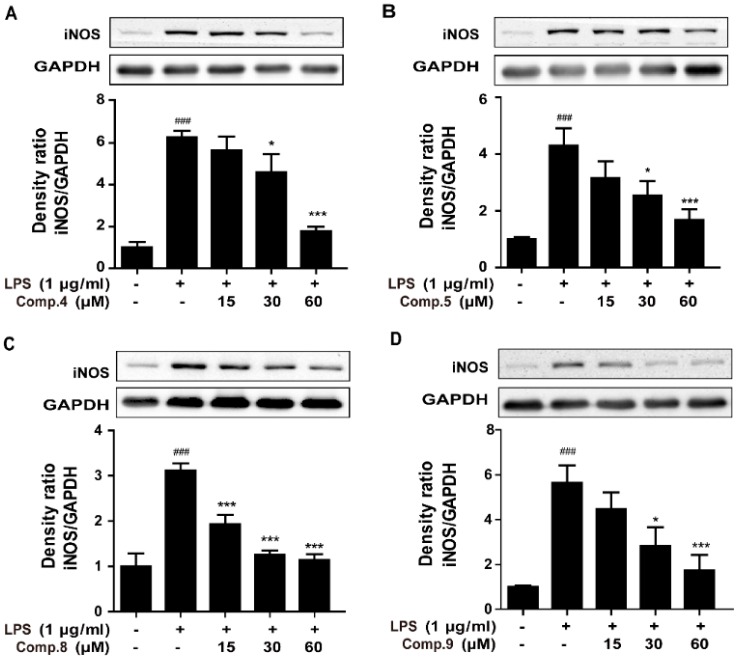
Effect of **4**, **5**, **8**, and **9** on iNOS expression in LPS-induced RAW 264.7 cells. Cells were pretreated with varying concentrations (0–60 μM) of **4**, **5**, **8** and **9** for 2 h and then stimulated with or without LPS (1 μg/mL) for 24 h. Protein levels of iNOS were evaluated by western blot ((**A**–**D**), for compounds **4**, **5**, **8**, and **9** respectively). The data show mean ± SD values. ^###^, *p* < 0.001 was compared to control. *, *p* < 0.05, ***, *p* < 0.001 were versus LPS.

Inhibition of NO overproduction through blocking inducible nitric synthase (iNOS) expression have been proved to be potential target of anti-inflammatory drug [24]. In the following, the inhibitory effect of compounds **4**, **5**, **8**, and **9** on iNOS expression in LPS-stimulated RAW 264.7 cells were investigated. Western blotting analysis revealed that compounds **4**, **5**, **8**, and **9** markedly suppressed the iNOS protein expression in LPS-induced RAW 264.7 cells in a concentration-dependent manner (Figure 5). The downregulation of the expression of iNOS corresponded to the reduction of the production of NO.

### 2.4. Compounds ***4***, ***5***, ***8***, and ***9*** Suppressed Release of IL-6 and TNF-α in LPS-Induced RAW 264.7 Cells

**Figure 6:**
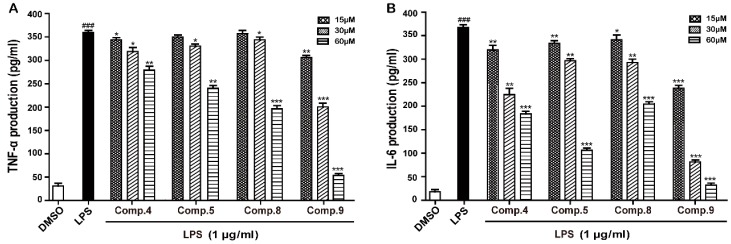
Inhibition of compounds **4**, **5**, **8**, and **9** on TNF-α and IL-6 production in LPS-induced RAW 264.7 cells. The cells were pretreated with various concentrations (0–60 μM) of **4**, **5**, **8** and **9** for 2 h and then incubated with or without LPS (1 μg/mL) for 24 h. Extracellular production of TNF-α (**A**) and IL-6 (**B**) in culture media were measured using commercial ELISA kits. The data show mean ± SD values. ^###^, *p* < 0.001 was compared to control. *, *p* < 0.05, **, *p* < 0.01, ***, *p* < 0.001 were versus LPS.

Proinflammatory cytokines, such as IL-6 and TNF-α produced by activated macrophages contribute to the inflammatory responses in inflammation-related diseases [25,26]. Therefore, they are regarded as targets for inhibiting the inflammatory process. To evaluate the effects of compounds **4**, **5**, **8**, and **9** on the production of proinflammatory mediators, the levels of IL-6 and TNF-α in the culture medium were measured by ELISA. In accordance with the NO results, compounds **4**, **5**, **8**, and **9** also significantly inhibited LPS-induced IL-6 and TNF-α released by RAW 264.7 cells in a concentration-dependent manner (Figure 6).

As illustrated in Figure 4B, anti-inflammatory effect of actinofuranones was dramatically influenced by the locality of hydroxyl substitute of C-5 unsaturated alkyl chains (**3** vs. **4**, **5**). Meanwhile, the more hydroxyl located at alkyl chain, the weaker anti-inflammation effect exhibited (**3**–**5** vs. **8**, **9**). The additional hydroxyl possibly adjust the physico-chemical properties of drugs and change their biological activities.

## 3. Materials and Methods

### 3.1. General Information

Optical rotations were determined using an MCP200 automatic polarimeter (Anton Paar, Graz, Austria). Ultraviolet spectra were recorded on a DU 730 nucleic acid/protein analyzer (Beckman Coulter, Brea, CA, USA). IR spectra (film) were measured with a Tensor 27 FT-IR spectrometer (Bruker, Ettlingen, Germany). 1D and 2D NMR spectra were recorded on a Bruker AV-600 spectrometer, *δ* in ppm rel. to TMS, J in Hz. ESIMS were recorded using an 1290-6420 Triple Quadrupole LC-MS spectrometer (Agilent, Santa Clara, CA, USA). HRESIMS were recorded with an Agilent G6230 TOF mass spectrometer. Silica gel (100–200 mesh, 300–400 mesh, Qingdao Marine Chemical Ltd., Qingdao, China), Sephadex LH-20 (GE Healthcare Bio-sciences AB, Uppsala, Sweden), YMC*GEL ODS-A (S-50 μm, 12 nm) (YMC Co., Ltd., Kyoto, Japan), and reversed-phase HPLC (Rohm and Hass Shanghai Chemical Industry Co., Ltd., Shanghai, China) were used for column chromatography. Biological assays were analyzed using a microplate reader (BioTek Synergy H1, BioTek Instruments, Winooski, VT, USA). 

### 3.2. Microbial Material

The strain (No. YIM 130461) was isolated from *Leptogium trichophorum* collected from an evergreen broad-leaf forest at an elevation of 2500 m in Benzilan, Diqing (Yunnan Province, China). On the basis of NCBI BLAST analysis of 16S rRNA gene sequences, this strain was identified as *Streptomyces gramineus* because it had 99.90% sequence identity with previously reported *S*. *gramineus* (GenBank accession no. HM748598). The strain (No. YIM 130461) was deposited at the Yunnan Institute of Microbiology, Yunnan University, China.

### 3.3. Fermentation, Extraction and Isolation

The strain, grown on agar plate, was prepared to inoculate 500 mL Erlenmeyer flasks each containing 100 mL of sterile seed medium composed of glucose 0.4%, yeast extract 0.4%, malt extract 0.5%, multiple vitamin (thiamine 0.5 mg, riboflavin 0.5 mg, niacin 0.5 mg, pyridoxine 0.5 mg, inositol 0.5 mg, calcium pentothenate 0.5 mg, *p*-aminobenzoic acid 0.5 mg, biotin 0.25 mg) 3.75 mg per liter, and trace element solution (2g L^−1^ FeSO_4_·7H_2_O, 1 g L^−1^ MnCl_2_·4H_2_O, 1 g L^−1^ ZnSO_4_·7H_2_O) 1.0 mL per liter at a pH of 7.2 with no adjustment. These flasks cultures were incubated at 28 °C for 2 days on a rotary shaker set at 180 rpm. For large-scale fermentation, 20 mL of seed medium was used to transferred into 1-L Erlenmeyer flasks containing 200 mL of sterile fermentation medium composed of 10 g L^−1^ soybean meal, 2 g L^−1^ peptone, 20 g L^−1^ glucose, 5 g L^−1^ soluble starch, 2 g L^−1^ yeast extract, 4 g L^−1^ NaCl, 0.5 g L^−1^ K_2_HPO_4_, 0.5 g L^−1^ MgSO_4_·7H_2_O, and 2 g L^−1^ CaCO_3_ at a pH of 7.8 with no adjustment. The fermentation batches were cultured at 28 °C for 7 days on a rotary shaker set at 180 rpm. The mycelium and broth filtrate (70 L) were separated by centrifugation (4000 rpm, 5 min). The resultant aqueous phase filtrate was extracted with EtOAc, then the organic partition layer was collected. Meanwhile, the mycelium cake was steeped in MeOH for 24 h to produce cell extracts. The methanol solution was centrifuged, concentrated, diluted with water and extracted with EtOAc. The combined extracts were evaporated *in vacuo* to yield 13 g of dried crude extract and further separated by chromatography on a silica gel column with a gradient of CH_2_Cl_2_-MeOH (*v*/*v* 50:1–1:1) to obtain nine fractions. Fraction 4 was subjected to Sephadex LH-20 chromatography (MeOH) to produce five subfractions. Fraction 4.2 was further separated by ODS column chromatography, eluting with MeOH-H_2_O (*v*/*v* 70:30) to yield **7** (21.0 mg), **8** (25.0 mg), **9** (5.0 mg). Fraction 6 was subjected to Sephadex LH-20 chromatography (MeOH) to afford six subfractions. Fraction 6.3 was purified using semi-preparative reversed-phase HPLC (CH_3_CN-H_2_O, *v*/*v* 38:62, 10 mL/min) to yield **5** (13.4 mg) and **6** (6.0 mg). Fraction 7 was separated by silica gel column chromatography, eluting with 15:1 CH_2_Cl_2_-MeOH to afford six subfractions. Subfraction 7.4 was applied to ODS column chromatography, eluting with MeOH-H_2_O (*v*/*v* 65:35) to yield **3** (5.0 mg). Subfraction 7.5 was purified by the ODS column chromatography (MeOH-H_2_O, *v*/*v* 60:40) to produce **2** (25.0 mg). Subfraction 7.6 was separated by semi-preparative reversed-phase HPLC on a C_18_ column (MeOH-H_2_O, *v*/*v* 60:40, 10 mL/min) to yield **4** (11.0 mg) and **1** (4.0 mg). 

*Actinofuranone D* (**1**): colorless amorphous powder; UV (MeOH) *λ*_max_ (log ε) 230 (4.3) nm, 286 (3.9) nm. IR (film) *ν*_max_ 3357, 2919, 2848, 1693, 1645, 1613, 1418, 1113,1020 cm^−1^. HR-ESI-MS *m/z* 461.2573 [M + Na]^+^ (calcd. for C_24_H_38_O_7_Na, 461.2515). For ^1^H- and ^13^C--NMR data see Table 1.

*Actinofuranone E* (**2**): colorless amorphous powder; UV (MeOH) *λ*_max_ (log ε) 230 (4.6) nm, 286 (4.2) nm. IR (film) *ν*_max_ 3356, 2920, 2850, 1693, 1613, 1409, 1104, 988cm^−1^. HR-ESI-MS *m/z* 461.2651 [M + Na]^+^ (calcd. for C_24_H_38_O_7_ Na, 461.2515). For ^1^H- and ^13^C-NMR data see Table 1.

*Actinofuranone F* (**3**): colorless amorphous powder; UV (MeOH) *λ*_max_ (log ε) 230 (4.6) nm, 286 (4.4) nm. IR (film) *ν*_max_ 3360, 2920, 2848, 1697, 1644, 1374, 1105, 990cm^−1^. HR-ESI-MS *m/z* 461.2654 [M + Na]^+^ (calcd. for C_24_H_38_O_7_Na, 461.2515). For ^1^H- and ^13^C-NMR data see Table 2. 

*Actinofuranone G* (**4**): colorless amorphous powder; UV (MeOH) *λ*_max_ (log ε) 230 (4.4) nm, 286 (4.3) nm. IR (film) *ν*_max_ 3359, 2920, 2850, 1696, 1615, 1410, 1106, 990 cm^−1^. HR-ESI-MS *m/z* 447.2363 [M + Na]^+^ (calcd. for C_23_H_36_O_7_Na, 447.2359). For ^1^H- and ^13^C-NMR data see Table 2.

*Actinofuranone H* (**5**): colorless amorphous powder; UV (MeOH) *λ*_max_ (log ε) 230 (4.5) nm, 286 (4.2) nm. IR (film) *ν*_max_ 3359, 2920, 2850, 1696, 1615, 1409, 1105, 990cm^−1^. HR-ESI-MS *m/z* 447.2374 [M + Na]^+^ (calcd. for C_23_H_36_O_7_Na, 447.2359). For ^1^H- and ^13^C-NMR data see Table 3.

*Actinofuranone I* (**6**): colorless amorphous powder; UV (MeOH) *λ*_max_ (log ε) 232 (4.5) nm, 286 (4.4) nm. IR (film) *ν*_max_ 3352, 2920, 2839, 1646, 1450, 1111, 1015 cm^−1^. HR-ESI-MS *m/z* 447.2337 [M + Na]^+^ (calcd. for C_23_H_36_O_7_Na, 447.2359). For ^1^H- and ^13^C-NMR data see Table 3.

### 3.4. Cell Culture and Vability Assay

RAW 264.7 cells were cultured in DMEM containing 10% fetal bovine serum (FBS), 2 mM *L*-glutamine, 100 U/mL penicillin, and 100 μg/mL streptomycin at 37 °C under 5% CO_2_. RAW 264.7 cells were plated at 2 × 10^5^ cells per well in 96-well-plates and incubated for 12 h, then treated with different treatment of compounds **1**–**9** (0–60 μM) and stimulated with or without lipopolysaccharides (LPS, 1 μg/mL) for 24 h. At the end of the treatment period, 3-(4,5-dimethylthiazol-2-yl)-2,5-diphenyl-tetrazolium bromide (MTT, 20 μL, 5 mg/mL) was added and incubated for additional 4 h. The supernatant was removed and dimethylsulfoxide (DMSO, 150 μL) was added for solubilization. The absorbance was read at 570 nm using a microplate reader.

### 3.5. Nitric Oxide (NO) Production Assay

RAW 264.7 cells were seeded onto 6-well-plate at a density of 1 × 10^6^ cells per well and cultured for 24 h. The cells were then pretreated with the varying concentrations of compounds **3**, **4**, **5**, **8** and **9** (0, 15, 30, and 60 μM) for 2 h before stimulation with LPS (1 μg/mL) for 24 h. After that, 50 μL of supernatant was mixed with equal volumes of Griess reagents (1% sulfanilamide, 0.1% *N*-(1-naphthyl) ethylenediamine dihydrochloride and 5% phosphoric acid) and incubated for 5 min at room temperature, the absorbance was measured at 540 nm using a microplate reader. The accumulation of nitrite was calculated using a standard curve of NaNO_2_.

### 3.6. Western Blot

RAW 264.7 cells were pretreated with compounds **4**, **5**, **8** and **9** (0, 15, 30, or 60 μM) for 2 h, then LPS (1 µg/mL) was added to stimulate for 24 h. Total cell protein was isolated with RIPA buffer (Beyotime, Beijing, China). After cell debris was discarded following centrifugation at 15,000 × *g* for 30 min, and the protein concentration measured by BCA protein assay (Beyotime, Beijing, China) according to the manufacturer’s protocols. Samples (30 µg) were separated on 10% SDS-PAGE and transferred onto PVDF (Millipore, Billerica, MA, USA) membrane, which was blocked with 5% non-fat milk in TBST (10% tris-buffered saline (tris base 24.2 g, NaCl 80 g, H_2_O 1 L), 0.1% polysorbate 20) for 1 h at room temperature followed by incubated with primary antibody overnight at 4 °C. The membrane was then washed three times with TBST and incubated with horseradish peroxidase conjugated secondary antibody for 1 h at room temperature, washed for three times with TBST and then developed by an enhanced chemiluminescence (ECL) detection system (Millipore, Billerica, MA, USA). Band pattern was analyzed with Bio-Rad ChemiDocTM XRS + System (Bio-Rad, Hercules, CA, USA).

### 3.7. Measurement of IL-6 and TNF-α by an Enzyme-Linked Immunosorbent Assay (ELISA)

RAW 264.7 cells were plated at a density of 1 × 10^6^ cells per well in 6-well plates and cultured for 24 h. The cells were then treated with various concentrations of compounds **4**, **5**, **8** and **9** (0, 15, 30, and 60 μM) and stimulated with or without of LPS (1 μg/mL) for 24 h. The levels of IL-6 and TNF-α in the cultured medium (supernatant) were determined using a commercially available ELISA kit (R&D Systems, Inc. Minneapolis, MN, USA) according to the manufacture’s protocols. The absorbance was measured at 450 nm with a microplate reader. IL-6 and TNF-α were determined from a standard curve. The concentrations were expressed as pg/mL.

### 3.8. Statistical Analysis

The measured data were expressed as mean ± the standard deviation. Student’s *t* test was performed using SPSS 10.0. A *p* value of <0.05 was considered as statistically significant.

## 4. Conclusions

Six new actinofuranones (compounds **1**–**6**) were isolated together with three known compounds from the ethyl acetate extract a fermentation broth of *S. gramineus* associated a with lichen. Compounds **4**, **5**, **8** and **9** significantly inhibited the production of NO through down-regulation of iNOS protein expression in LPS-stimulated RAW 264.7 cells. In addition, compounds **4**, **5**, **8** and **9** were also highly effective at inhibiting IL-6 and TNF-α production in the LPS-induced RAW 264.7 cells. These bioactive actinofuranones have potential usefulness in development of anti-inflammatory drugs. 

## Figures and Tables

**Table 1:** ^1^H-NMR (600 MHz) and ^13^C-NMR (150 MHz) data of compounds **1** and **2**.

No.	1				2			
	*δ*_C_*^a^*, Type	*δ*_H_*^a^,* [*J* in Hz]	*δ*_C_*^b^*, Type	*δ*_H_*^b^*, [*J* in Hz]	*δ*_C_*^a^*, Type	*δ*_H_*^a^*, [*J* in Hz]	*δ*_C_*^b^*, Type	*δ*_H_*^b^*, [*J* in Hz]
1	68.2 (69.3), CH	3.69 (3.74), m	70.4 (71.0), CH	3.90, m	68.2 (69.3), CH	3.69 (3.74), m	70.3 (70.9), CH	3.90, m
2	104.2 (104.6), C		105.6 (105.5), C		104.1 (104.6), C		105.5 (105.6), C	
3	202.9 (201.6), C		205.9 (204.4), C		202.9 (201.6), C		205.7 (204.2), C	
4	109.8 (110.4), C		112.2 (112.6), C		109.8 (110.4), C		112.0 (112.5), C	
5	185.4 (185.1), C		188.6 (188.2), C		185.4 (185.1), C		188.4 (187.9), C	
6	37.7 (37.4), CH_2_	2.58 (2.63), d [6.8] 2.58 (2.56), d [6.8]	38.4 (38.2), CH_2_	2.71 (2.76), m 2.71 (2.70), m	37.6 (37.4), CH_2_	2.58 (2.64), d [7.0] 2.58 (2.55), d [7.0]	38.1 (38.3), CH_2_	2.70 (2.75), d [7.7] 2.70 (2.69), d [7.7]
7	67.9 (67.7), CH	3.81 (3.86), m	67.9 (67.7), CH	3.98 (4.05), m	67.9 (67.7), CH	3.81 (3.86), m	69.6 (69.4), CH	3.98 (4.05), m
8	36.5 (36.7), CH_2_	1.60, m; 1.46, m	38.2 (38.1), CH_2_	1.64, m	36.6 (36.7), CH_2_	1.56, m; 1.46, m	38.1 (38.0), CH_2_	1.70, m; 1.62, m
9	28.6, CH_2_	2.17, m; 2.07, m	29.9, CH_2_	2.26, m; 2.18, m	28.6, CH_2_	2.16, m; 2.07, m	29.7, CH_2_	2.25, m; 2.17, m
10	132.0, CH	5.55, dt [14.2, 6.8]	132.2, CH	5.61, m	132.1, CH	5.55, dt [14.6, 7.0]	132.5, CH	5.59, dt [14.5, 7.0]
11	131.1, CH	5.97, m	132.7, CH	6.08, m	131.1, CH	5.93, m	132.5, CH	6.05, m
12	129.7, CH	5.94, m	132.2, CH	6.06, m	129.7, CH	5.90, m	131.8, CH	6.03, m
13	136.3, CH	5.61, dd [14.5, 7.3]	136.3, CH	5.58, m	135.4, CH	5.55, dd [14.6, 7.6]	135.6, CH	5.55, dd [14.5, 8.0]
14	40.4, CH	2.19, sextet [7.0]	41.7, CH	2.31, m	40.0, CH	2.25, sextet [7.0]	41.5, CH	2.35, sextet [7.0]
15	81.0, CH	3.50, dd, [7.8, 4.7]	83.3, CH	3.65, d, [8.5]	80.0, CH	3.56, brt, [5.6]	82.3, CH	3.70, d, [7.0]
16	136.7, C		139.2, C		136.9, C		139.2, C	
17	131.4, CH	5.17, d [8.7]	132.0, CH	5.30, d [8.7]	130.2, CH	5.22, d [8.6]	130.9, CH	5.33, d [8.7]
18	68.3, CH	4.10, m	69.8 (69.5), CH	4.27, m	68.3, CH	4.08, m	69.9, CH	4.26, m
19	31.0, CH_2_	1.42, m; 1.30, m	31.6, CH_2_	1.57, m; 1.42, m	31.0, CH_2_	1.43, m; 1.28, m	31.5, CH_2_	1.57, m; 1.43, m
20	10.1, CH_3_	0.79, t [7.0]	10.2, CH_3_	0.88, t [7.2]	10.1, CH_3_	0.78, t [7.3]	10.1, CH_3_	0.88, t [7.2]
21	17.1 (16.5), CH_3_	1.13 (1.04), d [6.4]	16.8 (16.4), CH_3_	1.29 (1.17), d [6.5]	17.1 (16.5), CH_3_	1.13 (1.04), d [6.4]	16.7 (16.3), CH_3_	1.29 (1.17), d [6.4]
22	6.0, CH_3_	1.53 (1.54), s	5.8, CH_3_	1.65 (1.64), s	6.0, CH_3_	1.53, s	5.7, CH_3_	1.65 (1.64), s
23	17.4, CH_3_	0.78, d [7.0]	18.0, CH_3_	0.85, d [7.2]	17.9, CH_3_	0.88, d [6.6]	18.1, CH_3_	0.95, d [7.2]
24	11.7, CH_3_	1.52, s	11.7, CH_3_	1.65, s	13.0, CH_3_	1.50, s	12.7, CH_3_	1.63, s
1-OH		4.67 (4.86), d, [6.4]				4.66 (4.85), d [6.4]		
2-OH		7.25 (7.32), s				7.23 (7.35), s		
7-OH		4.70 (4.80), d [5.1]				4.68 (4.78), d [5.3]		
15-OH		4.61, d [4.6]				4.59, d [4.7]		
18-OH		4.45, d [4.8]				4.37, d [4.5]		

*^a^* Recorded at 150 MHz (^13^C) and 600 MHz (^1^H) in DMSO-*d*_6_. *^b^* Recorded at 150 MHz (^13^C) and 600 MHz (^1^H) in methanol-*d*_4_. The chemical shifts in parentheses belong to the C-1 epimer.

**Table 2:** ^1^H-NMR (600 MHz) and ^13^C-NMR (150 MHz) data of compounds **3** and **4** in DMSO-*d*_6_.

No.	3		4	
	*δ*_C_, Type	*δ*_H_, [*J* in Hz]	*δ*_C_, Type	*δ*_H_, [*J* in Hz]
1	68.3 (69.3), CH	3.70 (3.75), m	68.2 (69.3), CH	3.69 (3.74), m
2	104.1 (104.5), C		104.1 (104.6), C	
3	202.8 (201.6), C		202.9 (201.6), C	
4	109.8 (110.3), C		109.8 (110.4), C	
5	185.1 (185.4), C		185.4 (185.1), C	
6	37.8 (37.4), CH_2_	2.58 (2.62), m 2.58 (2.58), m	37.6 (37.4), CH_2_	2.58 (2.63), d [6.5] 2.58 (2.56), d [6.5]
7	67.9 (67.7), CH	3.81 (3.86), m	67.9 (67.7), CH	3.81 (3.86), m
8	36.6 (36.7), CH_2_	1.59, m; 1.45, m	36.6 (36.7), CH_2_	1.57, m; 1.45, m
9	28.6, CH_2_	2.16, m; 2.08, m	28.6, CH_2_	2.16, m; 2.08, m
10	131.9, CH	5.54, dt [14.0, 6.6]	132.0, CH	5.54, dt [13.6, 6.7]
11	131.1, CH	5.96, m	131.1, CH	5.96, m
12	129.6, CH	5.92, m	129.7, CH	5.92, m
13	136.1, CH	5.58, dd [14.8, 7.5]	135.8, CH	5.56, dd [15.1, 7.5]
14	40.4, CH	2.21, sextet [7.3]	40.4, CH	2.22, sextet [7.0]
15	81.0, CH	3.53, dd [7.7, 3.0]	80.2, CH	3.52, dd, [7.0, 4.8]
16	138.2, C		135.8, C	
17	123.1, CH	5.28, t [7.3]	132.0, CH	5.25, d [8.1]
18	37.8 CH_2_	2.08, m; 1.98, m	63.0, CH	4.35, m
19	66.6, CH	3.58, q [6.0]	24.4, CH_3_	1.05, d [6.2]
20	23.4, CH_3_	1.00, d [6.2]	17.1 (16.5), CH_3_	1.13 (1.04), d [6.4]
21	17.1 (16.5), CH_3_	1.14 (1.04), d [6.4]	6.0, CH_3_	1.53, s
22	6.0, CH_3_	1.53, s	17.7, CH_3_	0.84, d [6.8]
23	17.6, CH_3_	0.79, d [6.9]	12.4 (12.9), CH_3_	1.50, s
24	12.0, CH_3_	1.49, s		
1-OH		4.68 (4.90), brs		4.67 (4.85), d [6.4]
2-OH		7.33, s		7.23 (7.35), s
7-OH		4.72 (4.79), brs		4.69 (4.78), d [5.4]
15-OH		4.52, d [3.4]		
18-OH				4.57, d [4.6]
19-OH		4.40, brs		4.44, d [4.4]

The chemical shifts in parentheses belong to the C-1 epimer.

**Table 3:** ^1^H-NMR (600 MHz) and ^13^C-NMR (150 MHz) data of compounds **5**–**7** in DMSO-*d*_6_.

No.	5		6		7	
	*δ*_C_, Type	*δ*_H_, [*J* in Hz]	*δ*_C_, Type	*δ*_H_, [*J* in Hz]	*δ*_C_, Type	*δ*_H_, [*J* in Hz]
1	68.3 (69.3), CH	3.69 (3.74), m	68.2 (69.3), CH	3.69 (3.74), m	68.4 (69.4), CH	3.70 (3.74), m
2	104.2 (104.6), C		104.2 (104.6), C		104.3 (104.7), C	
3	202.9 (201.6), C		202.9 (201.6), C		203.0 (201.7), C	
4	109.9 (110.5), C		109.8 (110.4), C		109.9 (110.5), C	
5	185.3 (185.1), C		185.4 (185.1), C		185.5 (185.2), C	
6	37.7 (37.4), CH_2_	2.62, (2.62, 2.58), m	37.6 (37.4), CH_2_	2.56 (2.62, 2.56), m	37.7 (37.5), CH_2_	2.57 (2.64, 2.56), d [7.0]
7	66.5 (66.3), CH	3.90 (3.96), m	67.9 (67.7), CH	3.81 (3.86), m	67.8 (68.0), CH	3.81 (3.86), m
8	44.5 (44.6), CH_2_	1.61 (1.59, 1.46), m	36.5 (36.6), CH_2_	1.59, m; 1.46, m	36.7 (36.8), CH_2_	1.57 (1.56), m; 1.45 (1.46), m
9	69.2 (69.1), CH	4.16, m	28.7, CH_2_	2.18, m; 2.09, m	28.7, CH_2_	2.18, m; 2.07, m
10	135.3, CH	5.54, dt [15.1, 6.2]	132.9 (132.8), CH	5.59, dt [15.1, 7.0]	132.1, CH	5.55 (5.53), dt [14.8, 7.0]
11	130.1, CH	6.10, m	130.7, CH	6.08, dd, [15.1, 10.5]	131.3, CH	5.95, m
12	129.2, CH	5.96, dd [15.2, 10.4]	127.3 (127.2), CH	5.99, dd [15.1, 10.5]	129.7, CH	5.95, m
13	137.8, CH	5.67, dd [15.3, 7.4]	138.5 (138.6), CH	5.65, brd [15.1]	136.3, CH	5.59 (5.55), dd [15.0, 7.5]
14	40.4, CH	2.23, sextet [7.0]	74.7, C		40.0, CH	2.21, sextet [7.0]
15	80.8, CH	3.52, dd, [7.5, 3.8]	82.6, CH	3.63, d, [4.4]	81.1, CH	3.53, dd [7.8, 4.4]
16	136.5, C		135.4, C		137.4, C	
17	127.7, CH	5.25, t [7.5]	129.3, CH	5.23, t [7.0]	126.0, CH	5.24, t [7.3]
18	20.6, CH_2_	1.97, quint [7.5]	20.7, CH_2_	1.95, quint [7.3]	29.5, CH_2_	1.94, q [7.3]
19	14.5, CH_3_	0.90, t [7.5]	14.4, CH_3_	0.89, t [7.5]	22.8, CH_2_	1.32 m
20	17.1, CH_3_	1.14 (1.04), d [7.5]	17.1 (16.5), CH_3_	1.13 (1.04), d [6.4]	14.1, CH_3_	0.85, t [7.4]
21	6.0, CH_3_	1.53, s	6.0, CH_3_	1.53, s	17.2 (16.6), CH_3_	1.14 (1.04), d [6.4]
22	17.5, CH_3_	0.80, d [7.0]	25.4 (25.5), CH_3_	1.06, s	6.1, CH_3_	1.54, s
23	11.6, CH_3_	1.49, s	13.8, CH_3_	1.53, s	17.7, CH_3_	0.80, d [6.9]
24					11.9, CH_3_	1.49, s
1-OH		4.67 (4.85), brs		4.66 (4.85), d [6.4]		4.66 (4.84), d [4.5]
2-OH		7.25 (7.35), s		7.24 (7.35), s		7.23 (7.34), s
7-OH		4.72 (4.81), m		4.69 (4.78), d [5.3]		4.68 (4.78), brs
9-OH		4.76 (4.80), brs				
14-OH				4.17, s		
15-OH		4.54, d [3.8]		4.62, d [4.5]		4.52, d [3.6]
18-OH						

The chemical shifts in parentheses belong to the C-1 epimer.

## Supplementary Materials

Supplementary data associated with this article can be found in the online version.
