# Supplementary material for: Actinofuranones D-I from a Lichen-Associated Actinomycetes, Streptomyces gramineus, and Their Anti-Inflammatory Effects
Source: Molecules. 2018 Sep 18;23(9):2393. doi: 10.3390/molecules23092393 (PMC6225470; doi:10.3390/molecules23092393)
Supplement: Supplementary file 1 [file molecules-23-02393-s001.pdf]

Supplementary Materials

Actinofuranones D-I from lichen-associated actinomycetes *Streptomyces gramineus* with anti-inflammatory effect

Jian Ma [1], Bixuan Cao [1], Chengbin Liu [2], Peipei Guan [1], Yu Mu [1], Yi Jiang [2,]*, Li Han [1,]*, Xueshi Huang [1]

[1] Institute of Microbial Pharmaceuticals, College of Life and Health Sciences, Northeastern University, Shenyang 110819, P. R. China ; sherrie525358@126.com (J.M.); caobixuan1995@163.com (B.C.); guanpp@mail.neu.edu.cn (P.G.); muyu@mail.neu.edu.cn (Y.M.); huangxs@mail.neu.edu.cn (X.H.)

[2] Yunnan Institute of Microbiology, School of Life Science, Yunnan University, Kunming 650091, P. R. China; liuchengbin17@163.com (C.B)

* Corresponding authors.

Tel.: 0086-24-83656122; Fax: 0086-24-83656122; E-mail: hanli@mail.neu.edu.cn

Tel.: 0086-871-65034073; Fax: 0086-871-65173878; E-mail: jiangyi@ynu.edu.cn

Figure S1. HRESI-MS spectrum of the new compound **1**

Figure S2. IR spectrum of the new compound **1**

Figure S3. ^{1}H NMR (600 MHz, DMSO-d_6) spectrum of the new compound **1**

Figure S4. ^{1}H NMR (600 MHz, CD_3OD) spectrum of the new compound **1**

Figure S5. ^{13}C NMR (150 MHz, DMSO-d_6) spectrum of the new compound **1**

Figure S6. ^{13}C NMR (150 MHz, CD_3OD) spectrum of the new compound **1**

Figure S7. HSQC spectrum (DMSO-d_6) of the new compound **1**

Figure S8. HSQC spectrum (CD_3OD) of the new compound **1**

Figure S9. COSY spectrum (DMSO-d_6) of the new compound **1**

Figure S10. HMBC spectrum (DMSO-d_6) of the new compound **1**

Figure S11. NOESY spectrum (CD_3OD) of the new compound **1**

Figure S12. HRESI-MS spectrum of the new compound **2**

Figure S13. IR spectrum of the new compound **2**

Figure S14. ^{1}H NMR (600 MHz, DMSO-d_6) spectrum of the new compound **2**

Figure S15. ^{1}H NMR (600 MHz, CD_3OD) spectrum of the new compound **2**

Figure S16. ^{13}C NMR (150 MHz, DMSO-d_6) spectrum of the new compound **2**

Figure S17. ^{13}C NMR (150 MHz, CD_3OD) spectrum of the new compound **2**

Figure S18. HSQC spectrum (DMSO-d_6) of the new compound **2**

Figure S19. HSQC spectrum (CD_3OD) of the new compound **2**

Figure S20. COSY spectrum (DMSO-d_6) of the new compound **2**

Figure S21. COSY spectrum (CD_3OD) of the new compound **2**

Figure S22. HMBC spectrum (DMSO-d_6) of the new compound **2**

Figure S23. NOESY spectrum (CD_3OD) of the new compound **2**

Figure S24. HRESI-MS spectrum of the new compound **3**

Figure S25. IR spectrum of the new compound **3**

Figure S26. ^{1}H NMR (600 MHz, DMSO-d_6) spectrum of the new compound **3**

Figure S27. ^{1}H NMR (600 MHz, CD_3OD) spectrum of the new compound **3**

Figure S28. ^{13}C NMR (150 MHz, DMSO-d_6) spectrum of the new compound **3**

Figure S29. HSQC spectrum of the new compound **3**

Figure S30. COSY spectrum (DMSO-d_6) of the new compound **3**

Figure S31. HMBC spectrum (DMSO-d_6) of the new compound **3**

Figure S32. NOESY spectrum (CD_3OD) of the new compound **3**

Figure S33. HRESI-MS spectrum of the new compound **4**

Figure S34. IR spectrum of the new compound **4**

Figure S35. ^{1}H NMR (600 MHz, DMSO-d_6) spectrum of the new compound **4**

Figure S36. ^{13}C NMR (150 MHz, DMSO-d_6) spectrum of the new compound **4**

Figure S37. HSQC (DMSO-d_6) spectrum of the new compound **4**

Figure S38. COSY (DMSO-d_6) spectrum of the new compound **4**

Figure S39. HMBC (DMSO-d_6) spectrum of the new compound **4**

Figure S40. NOESY (CD_3OD) spectrum of the new compound **4**

Figure S41. HRESI-MS spectrum of the new compound **5**

Figure S42. IR spectrum of the new compound **5**

Figure S43. ^{1}H NMR (600 MHz, DMSO-d_6) spectrum of the new compound **5**

Figure S44. ^{1}H NMR (600 MHz, CD_3OD) spectrum of the new compound **5**

Figure S45. ^{13}C NMR (150 MHz, DMSO-d_6) spectrum of the new compound **5**

Figure S46. HSQC (DMSO-d_6) spectrum of the new compound **5**

Figure S47. COSY (DMSO-d_6) spectrum of the new compound **5**

Figure S48. HMBC (DMSO-d_6) spectrum of the new compound **5**

Figure S49. NOESY spectrum (CD_3OD) of the new compound **5**

Figure S50. HRESI-MS spectrum of the new compound **6**

Figure S51. IR spectrum of the new compound **6**

Figure S52. ^{1}H NMR (600 MHz, DMSO-d_6) spectrum of the new compound **6**

Figure S53. ^{13}C NMR (150 MHz, DMSO-d_6) spectrum of the new compound **6**

Figure S54. HSQC (DMSO-d_6) spectrum of the new compound **6**

Figure S55. COSY (DMSO-d_6) spectrum of the new compound **6**

Figure S56. HMBC (DMSO-d_6) spectrum of the new compound **6**

Figure S57. ^{1}H NMR (600 MHz, DMSO-d_6) spectrum of the compound **7**

Figure S58. ^{13}C NMR (150 MHz, DMSO-d_6) spectrum of the compound **7**

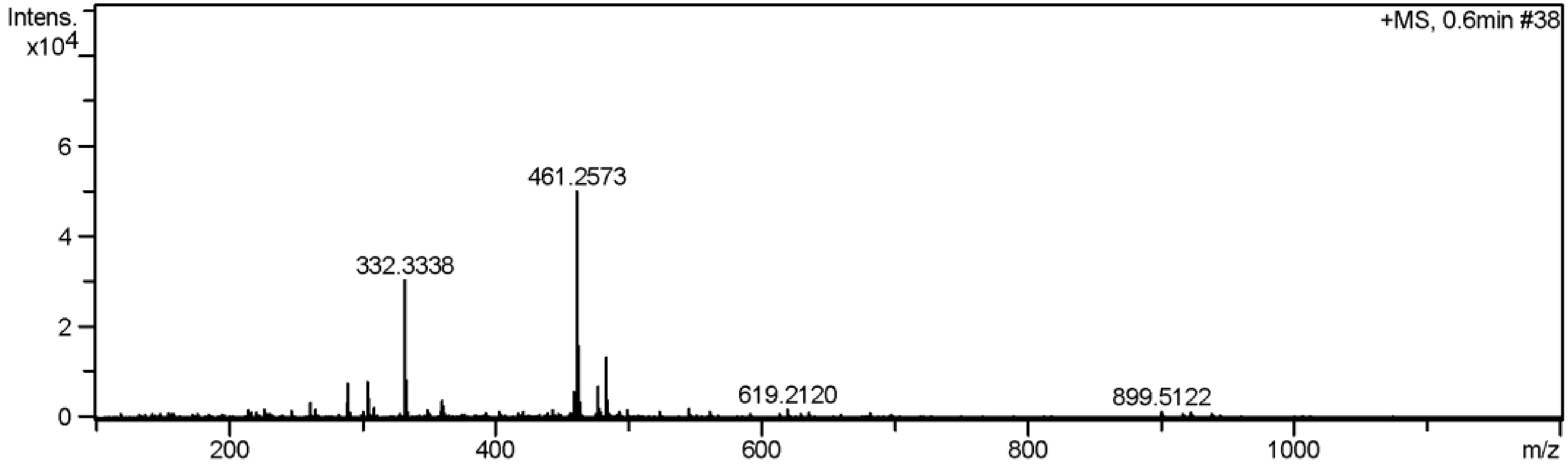

Figure S1. HRESI-MS spectrum of the new compound **1**

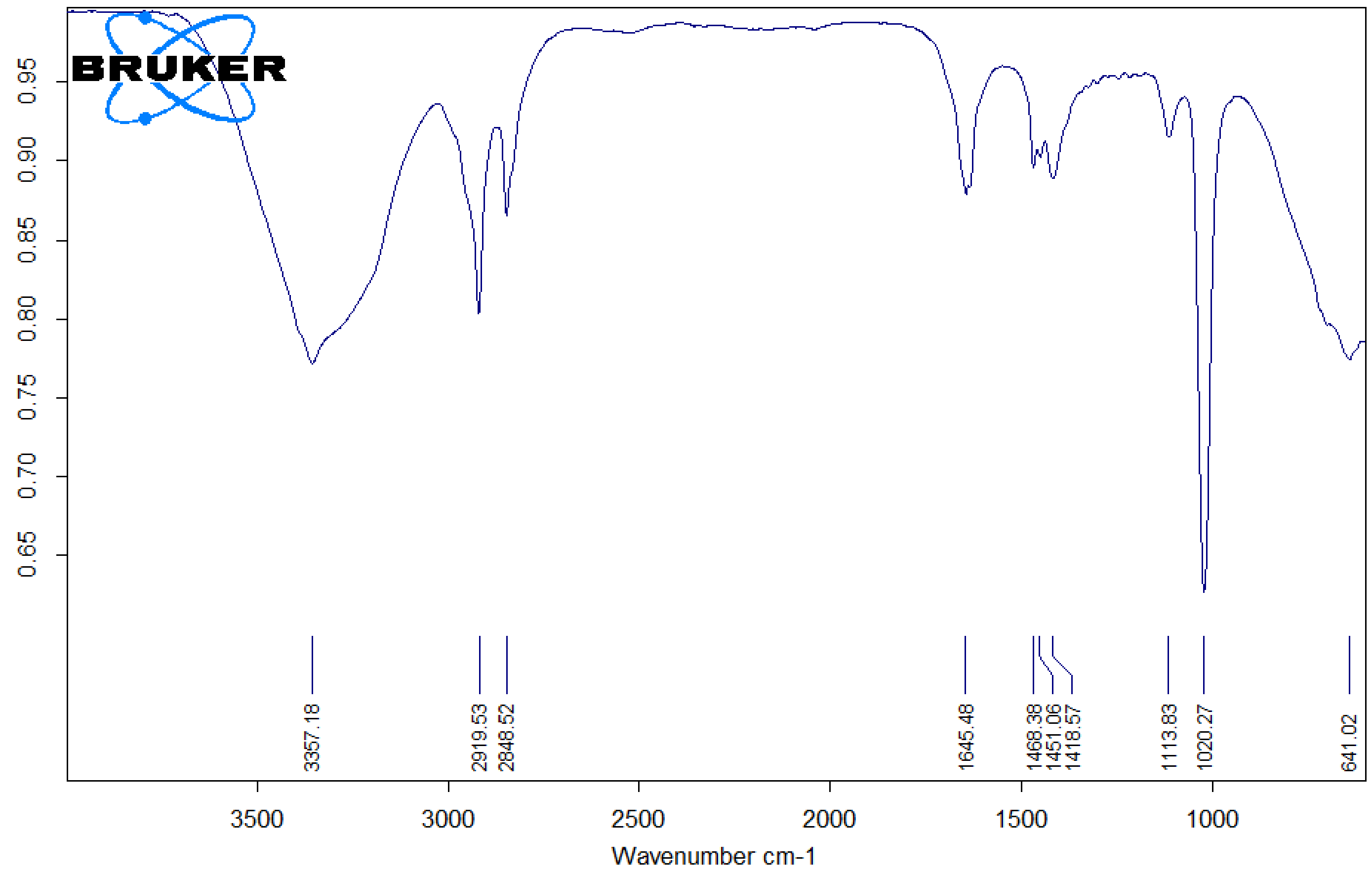

Figure S2. IR spectrum of the new compound **1**

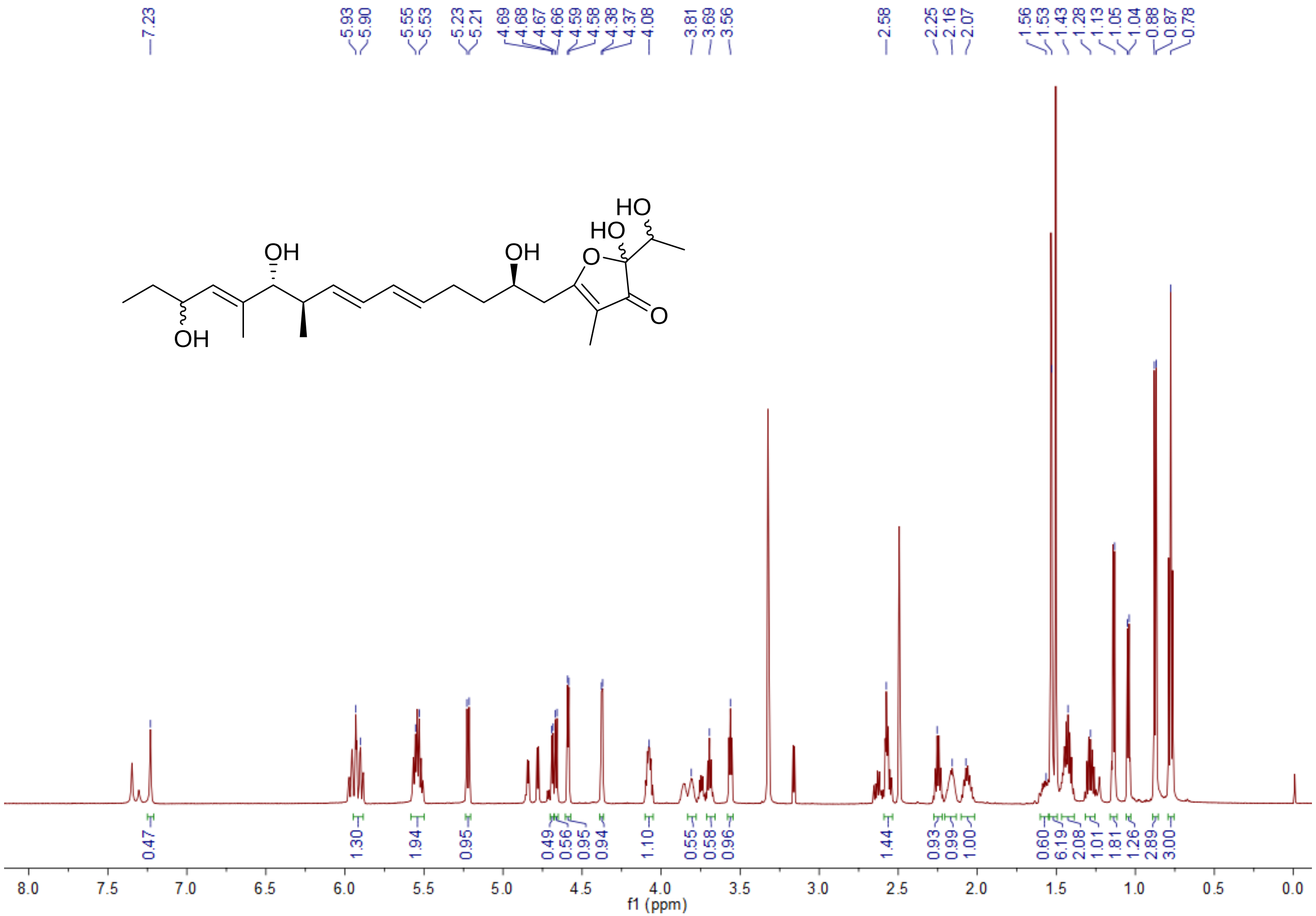

Figure S3. ^{1}H NMR (600 MHz, DMSO-d_6) spectrum of the new compound **1**

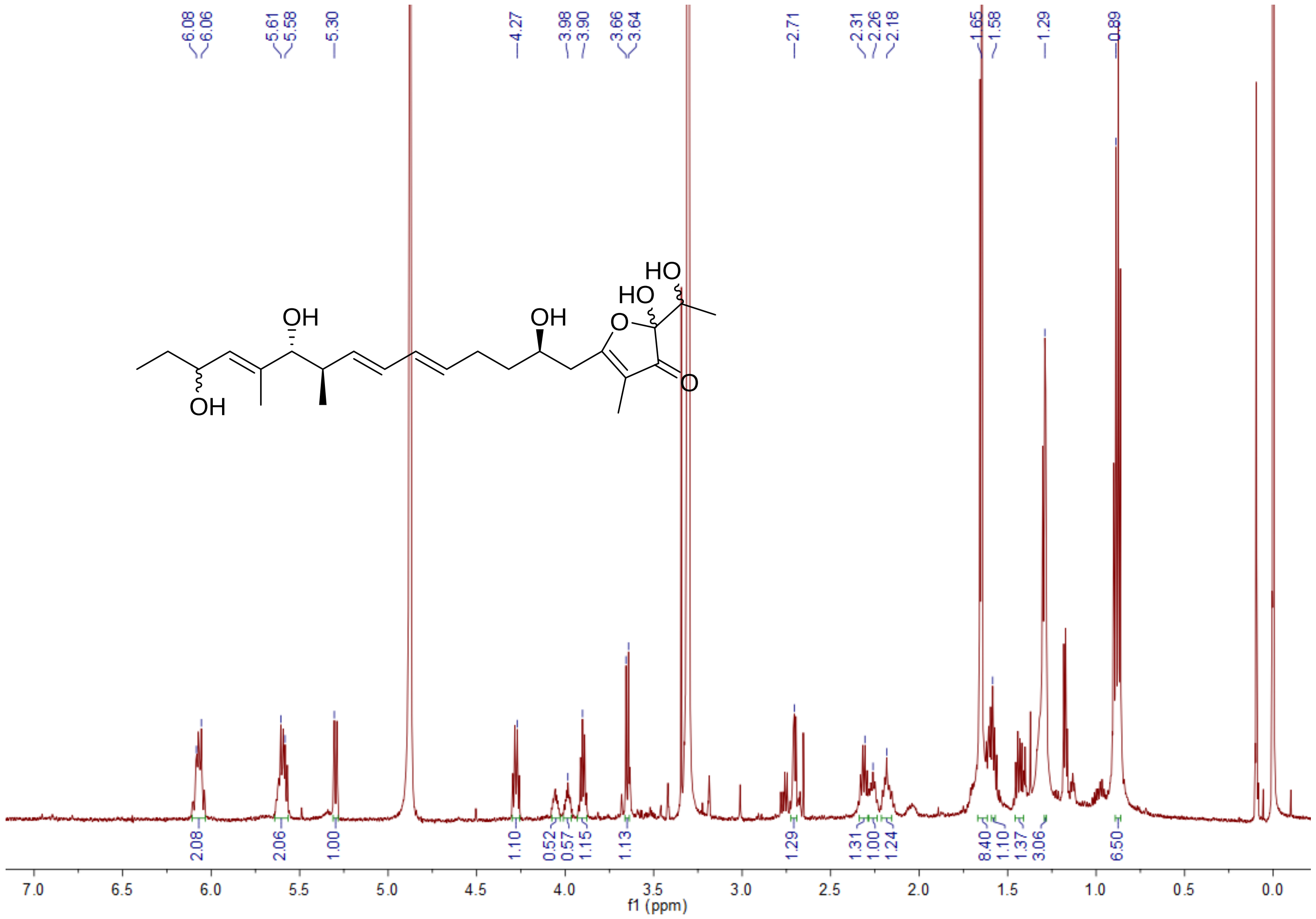

Figure S4. ^{1}H NMR (600 MHz, CD_3OD) spectrum of the new compound **1**

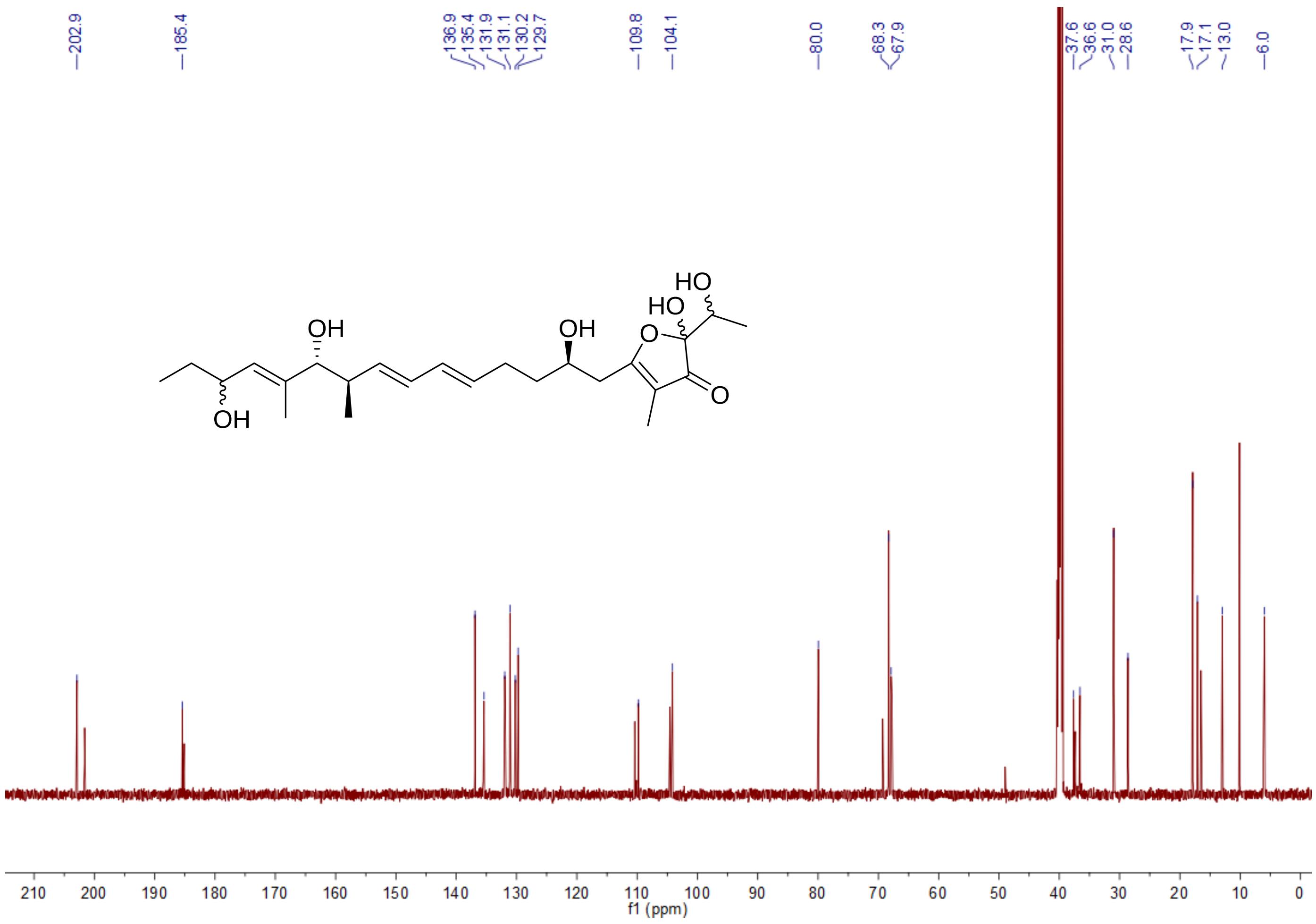

Figure S5. ^{13}C NMR (150 MHz, DMSO-d_6) spectrum of the new compound **1**

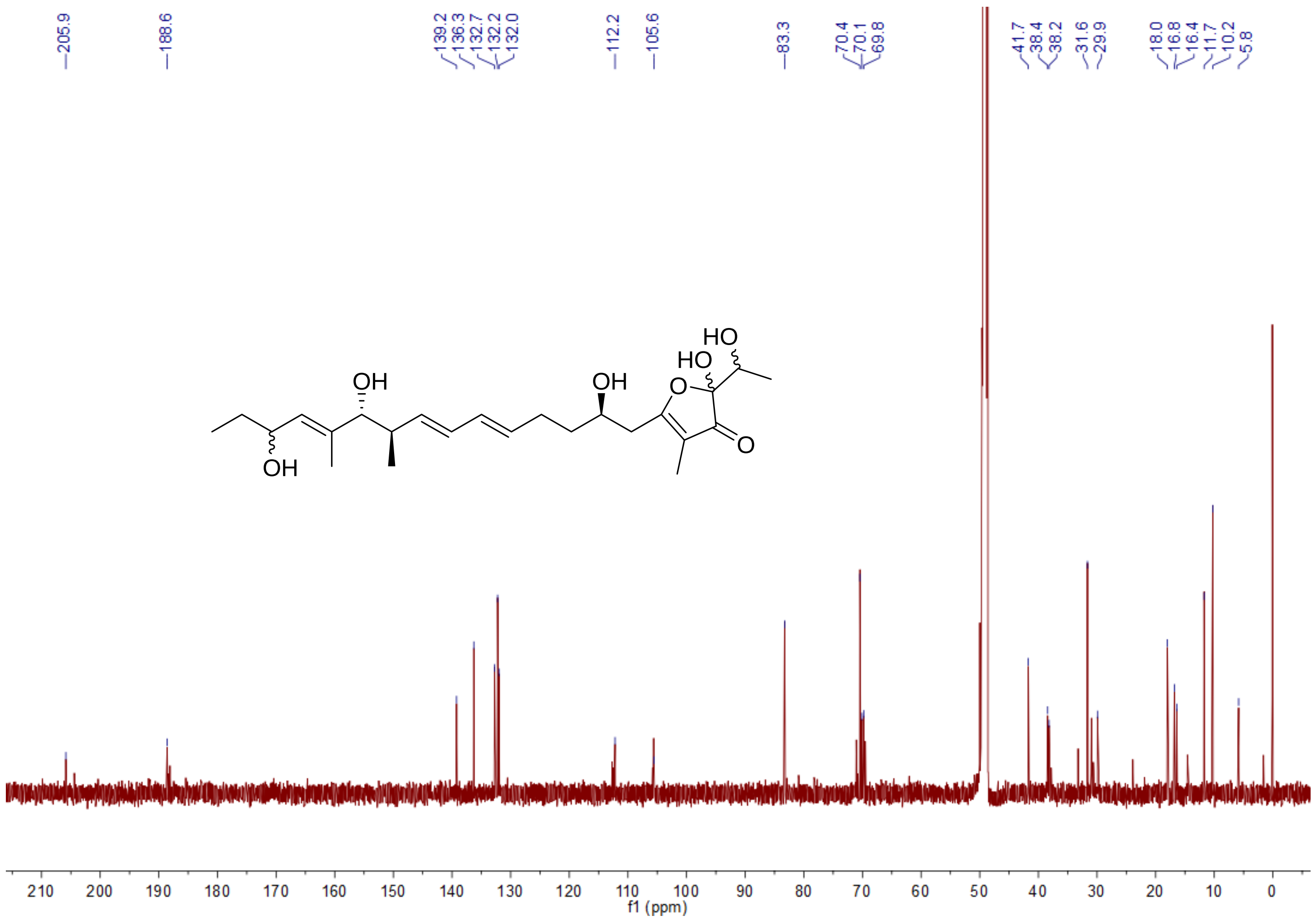

Figure S6. ^{13}C NMR (150 MHz, CD_3OD) spectrum of the new compound **1**

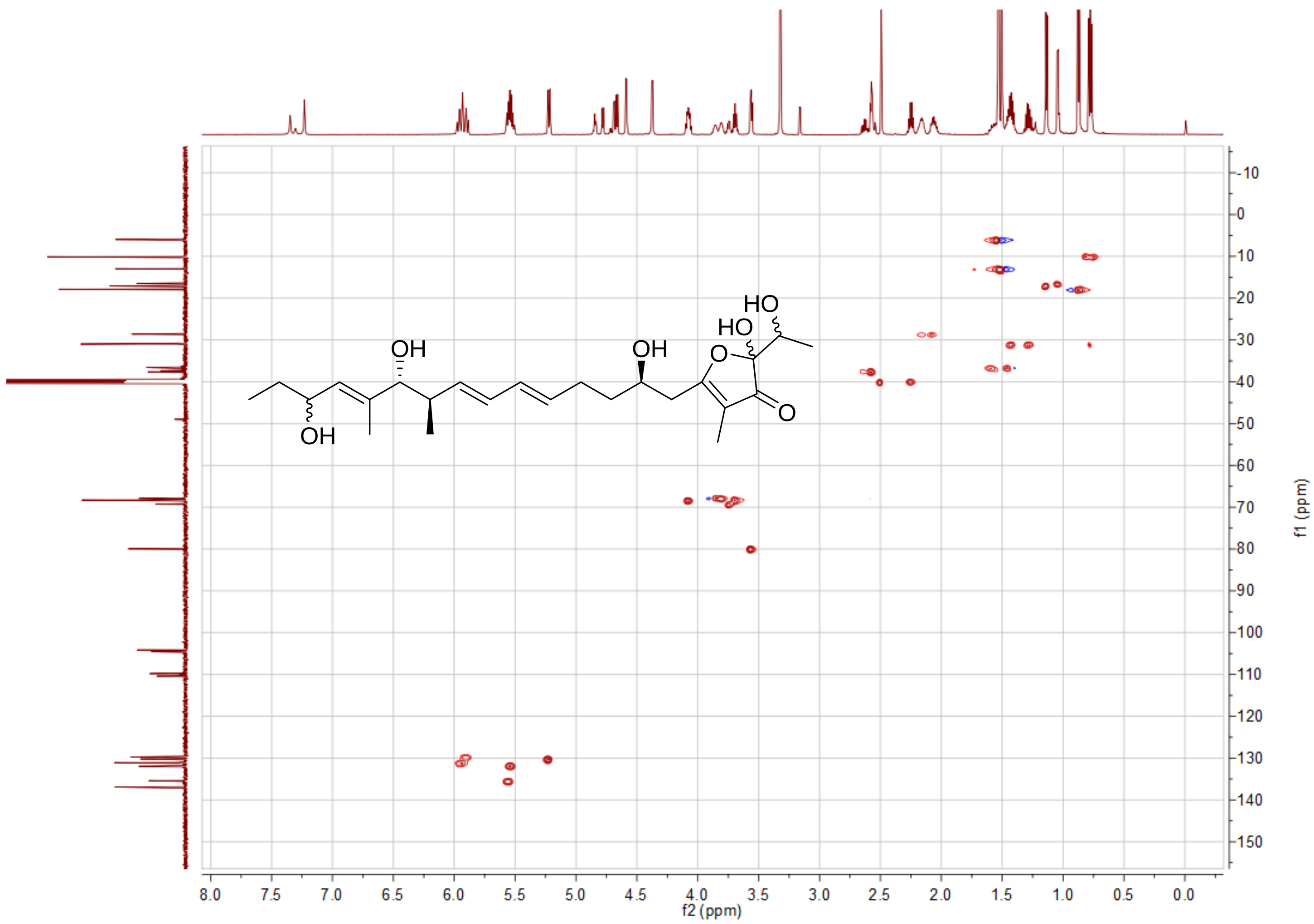

Figure S7. HSQC spectrum (DMSO-d_6) of the new compound **1**

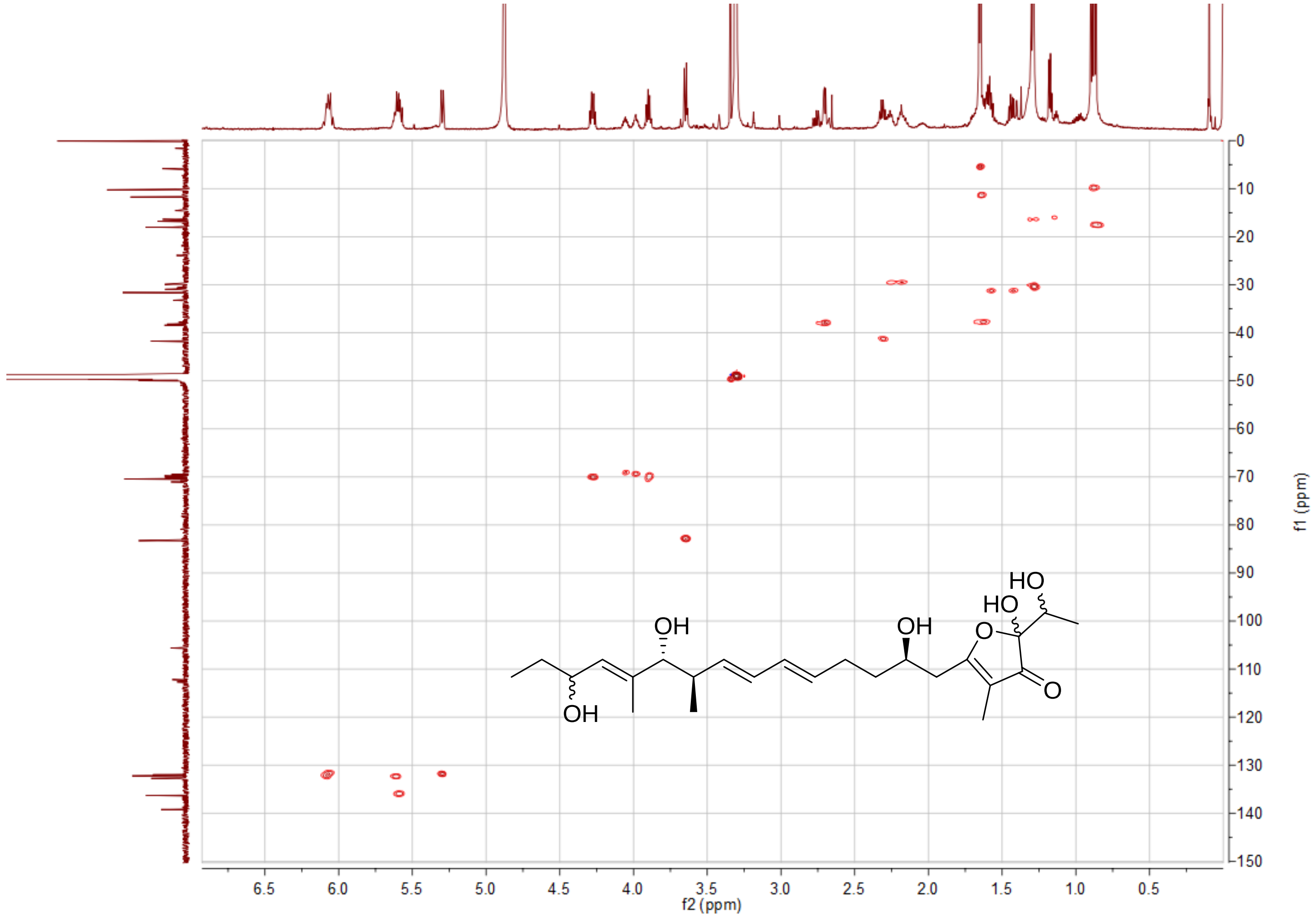

Figure S8. HSQC spectrum (CD_3OD) of the new compound **1**

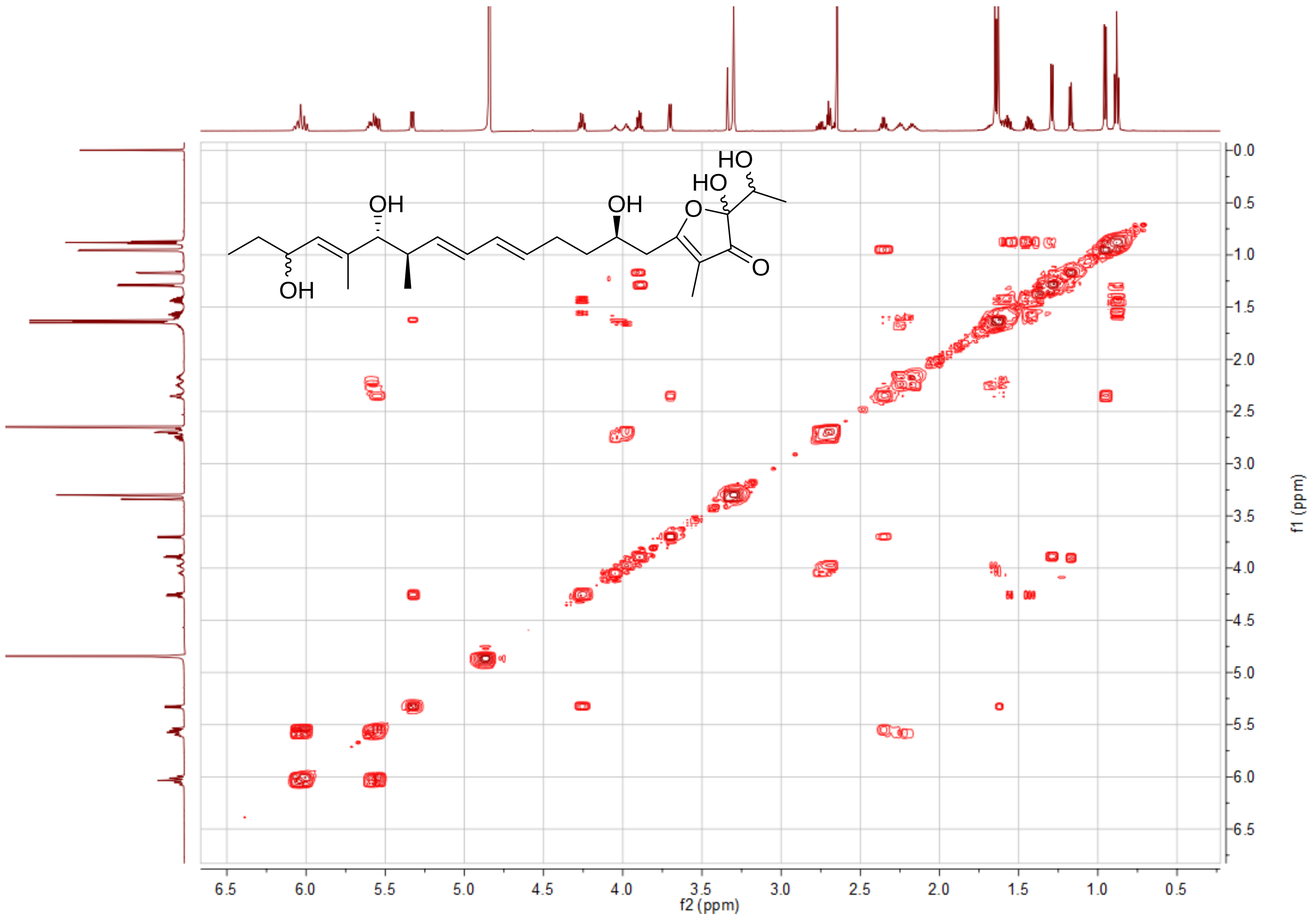

Figure S9. COSY spectrum (DMSO-d_6) of the new compound **1**

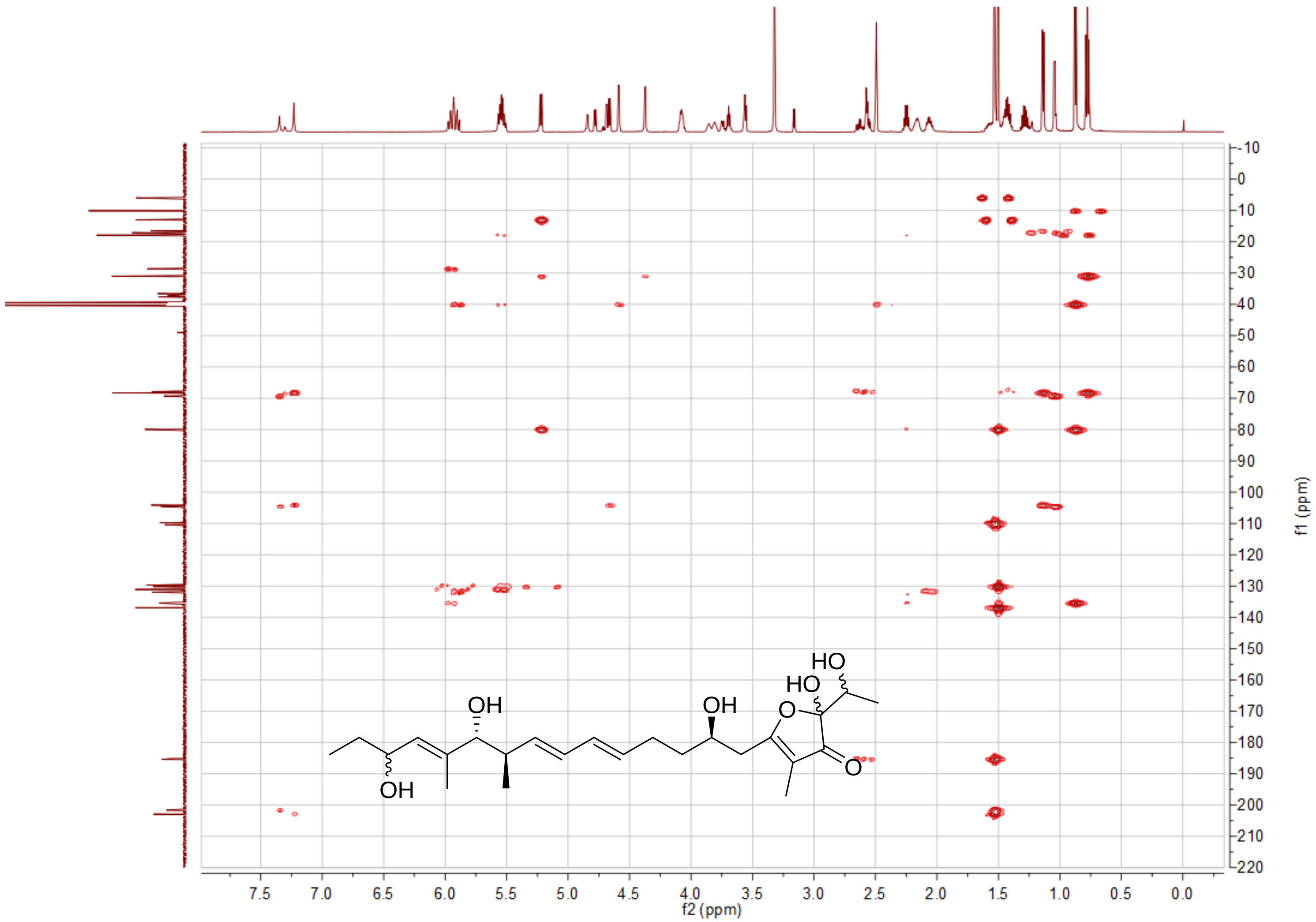

Figure S10. HMBC spectrum (DMSO-d_6) of the new compound **1**

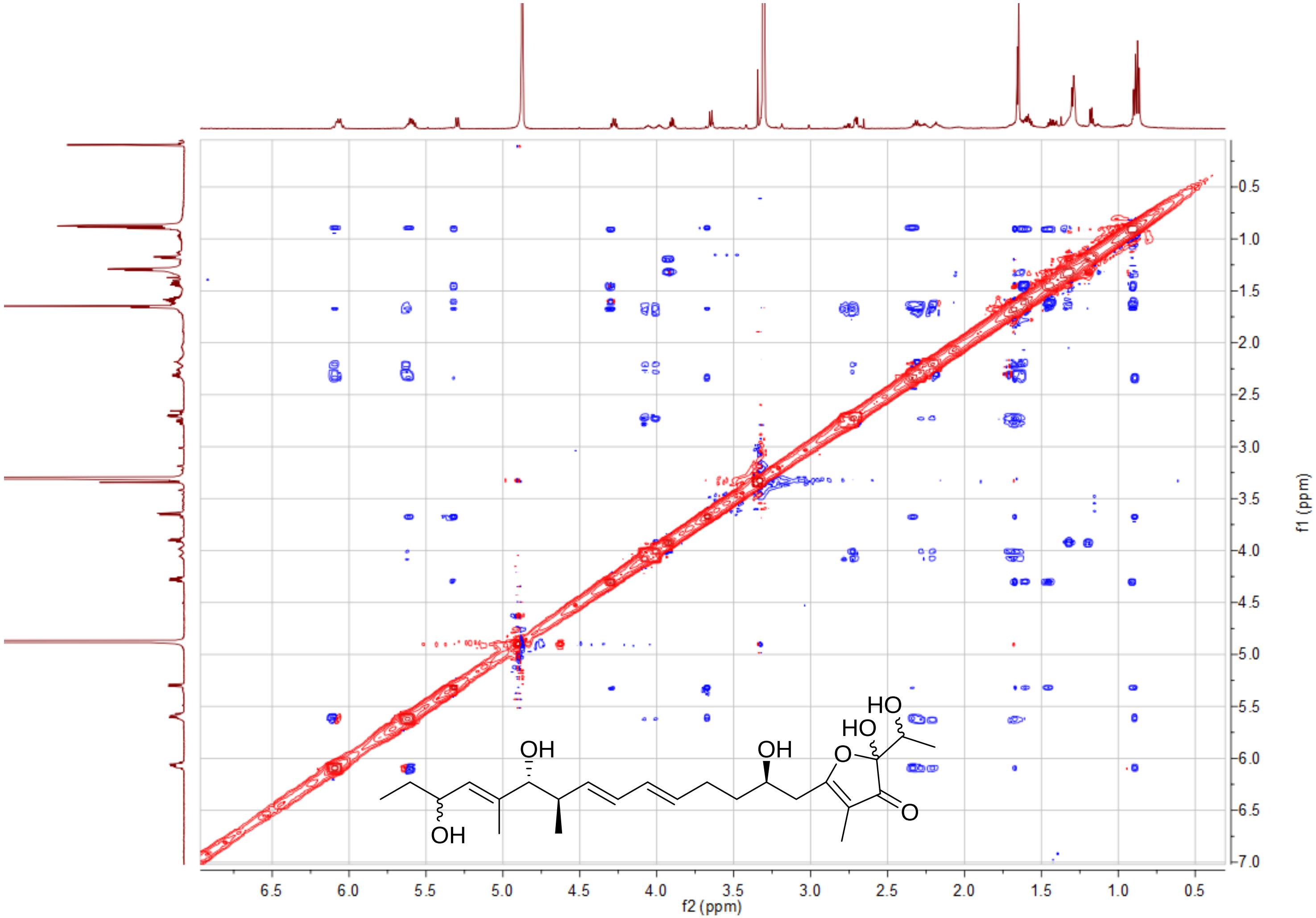

Figure S11. NOESY spectrum (CD_3OD) of the new compound **1**

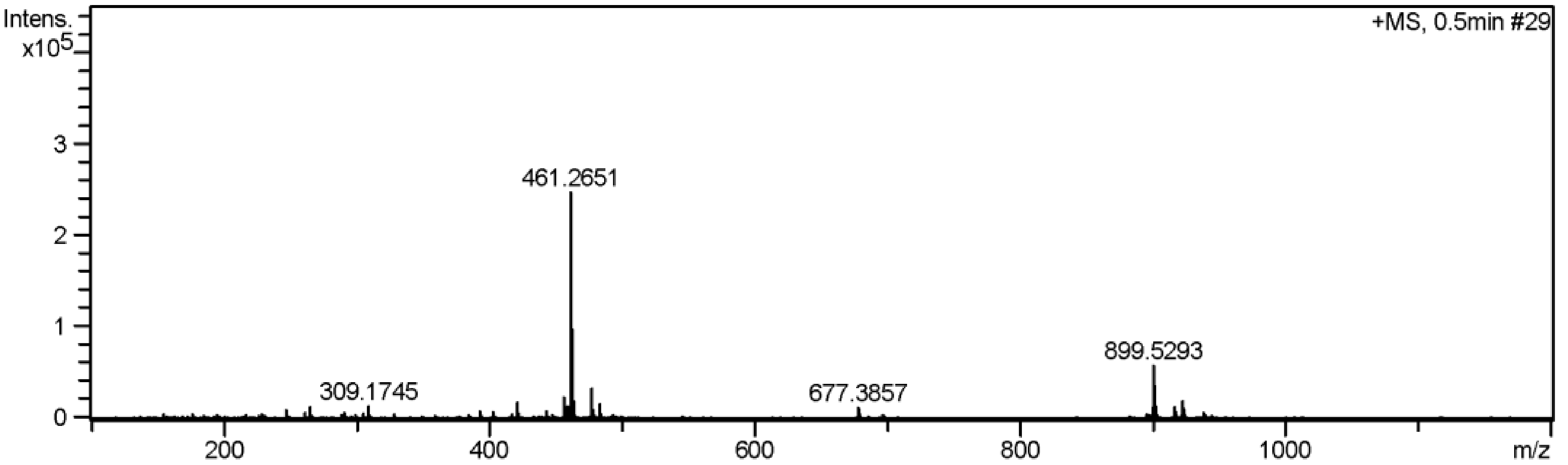

Figure S12. HRESI-MS spectrum of the new compound **2**

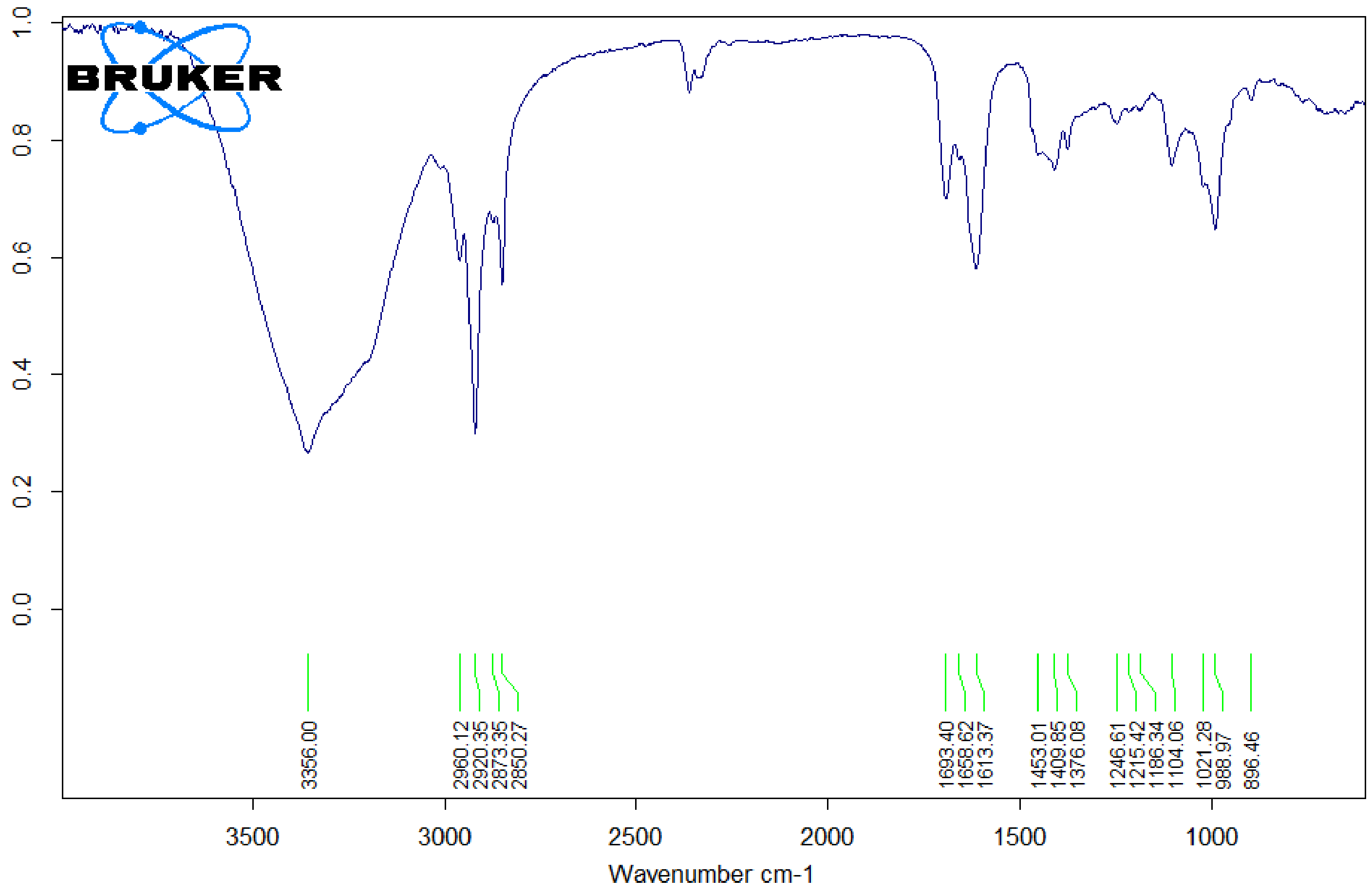

Figure S13. IR spectrum of the new compound **2**

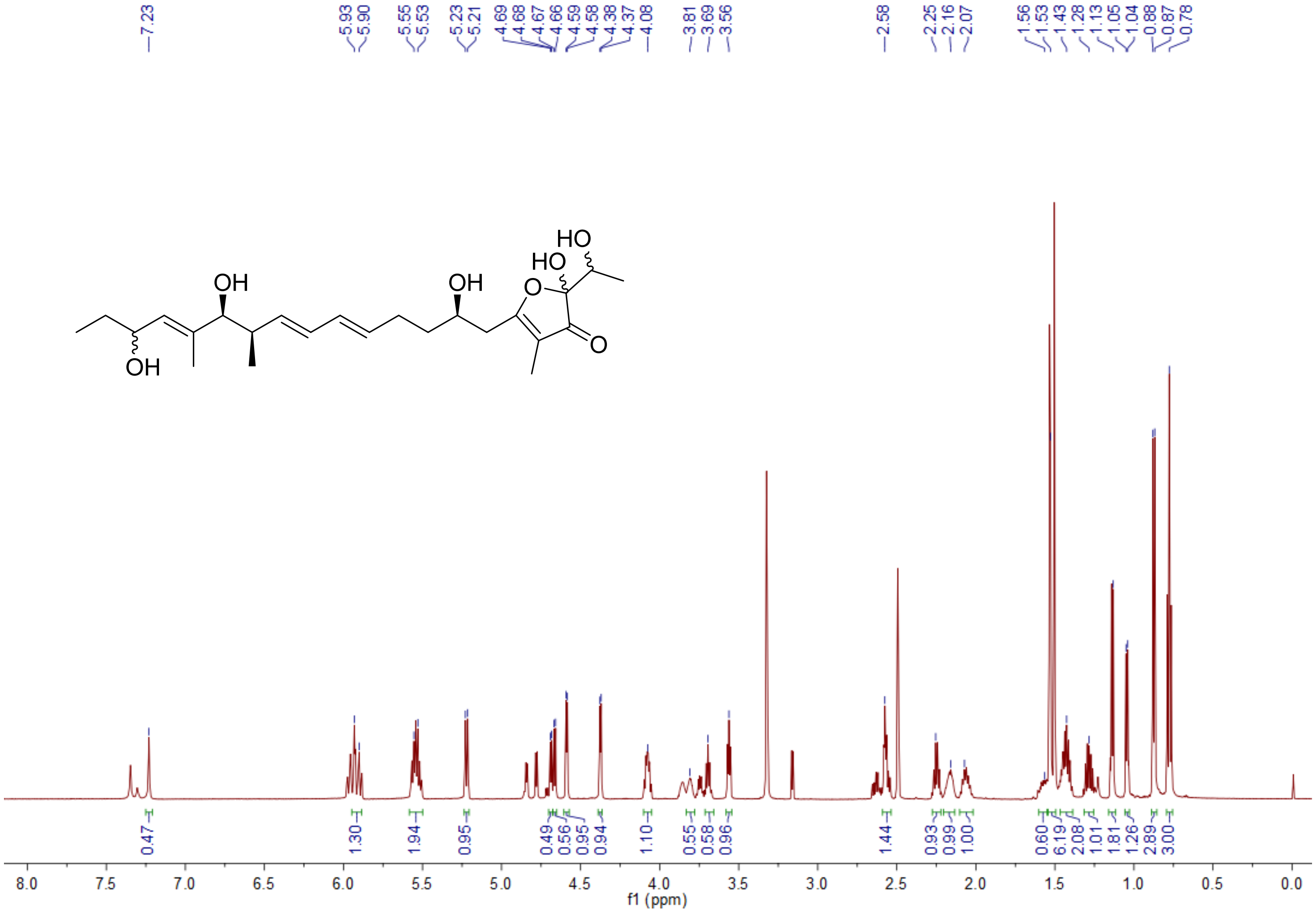

Figure S14. ^{1}H NMR (600 MHz, DMSO-d_6) spectrum of the new compound **2**

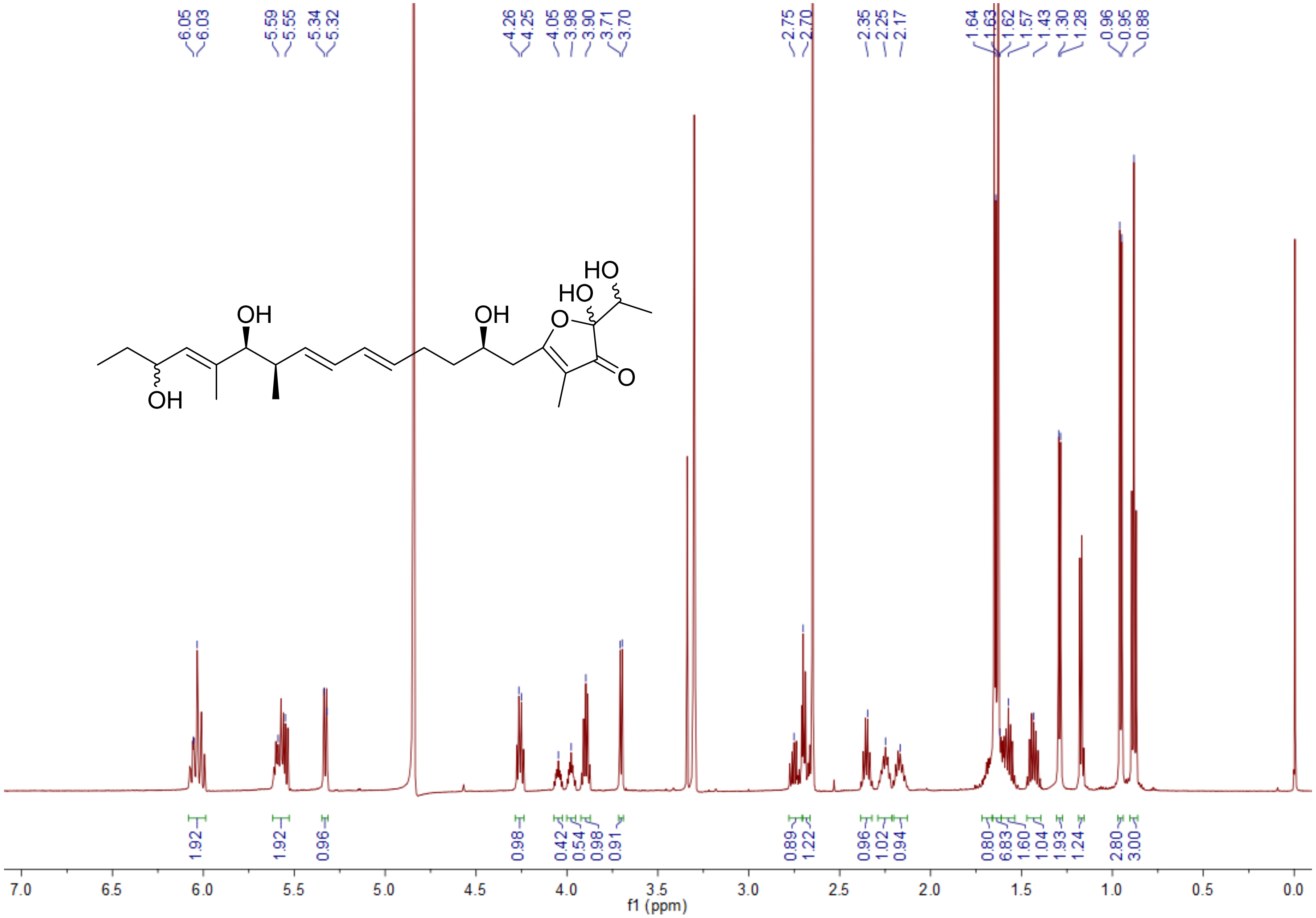

Figure S15. ^{1}H NMR (600 MHz, CD_3OD) spectrum of the new compound **2**

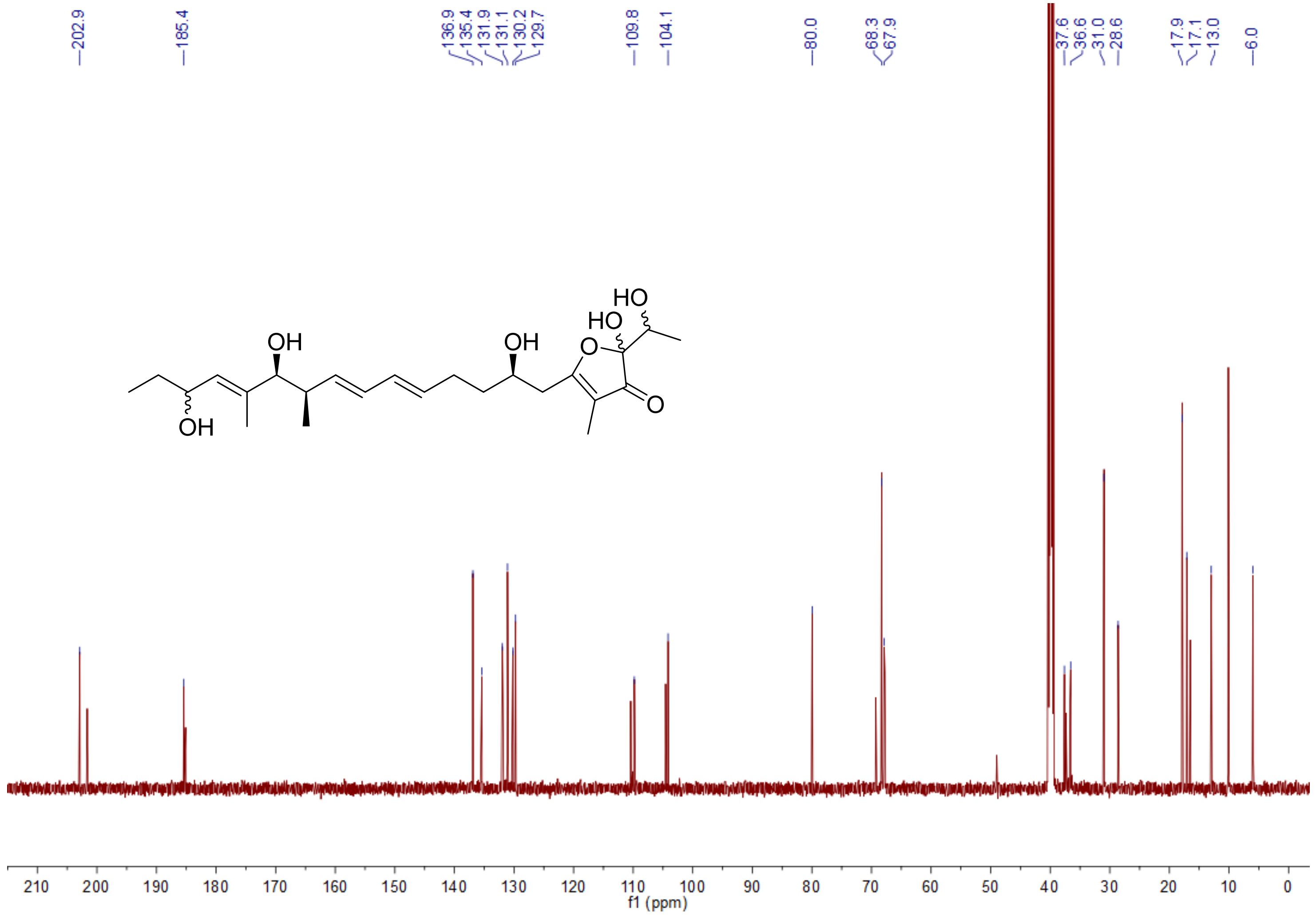

Figure S16. ^{13}C NMR (150 MHz, DMSO-d_6) spectrum of the new compound **2**

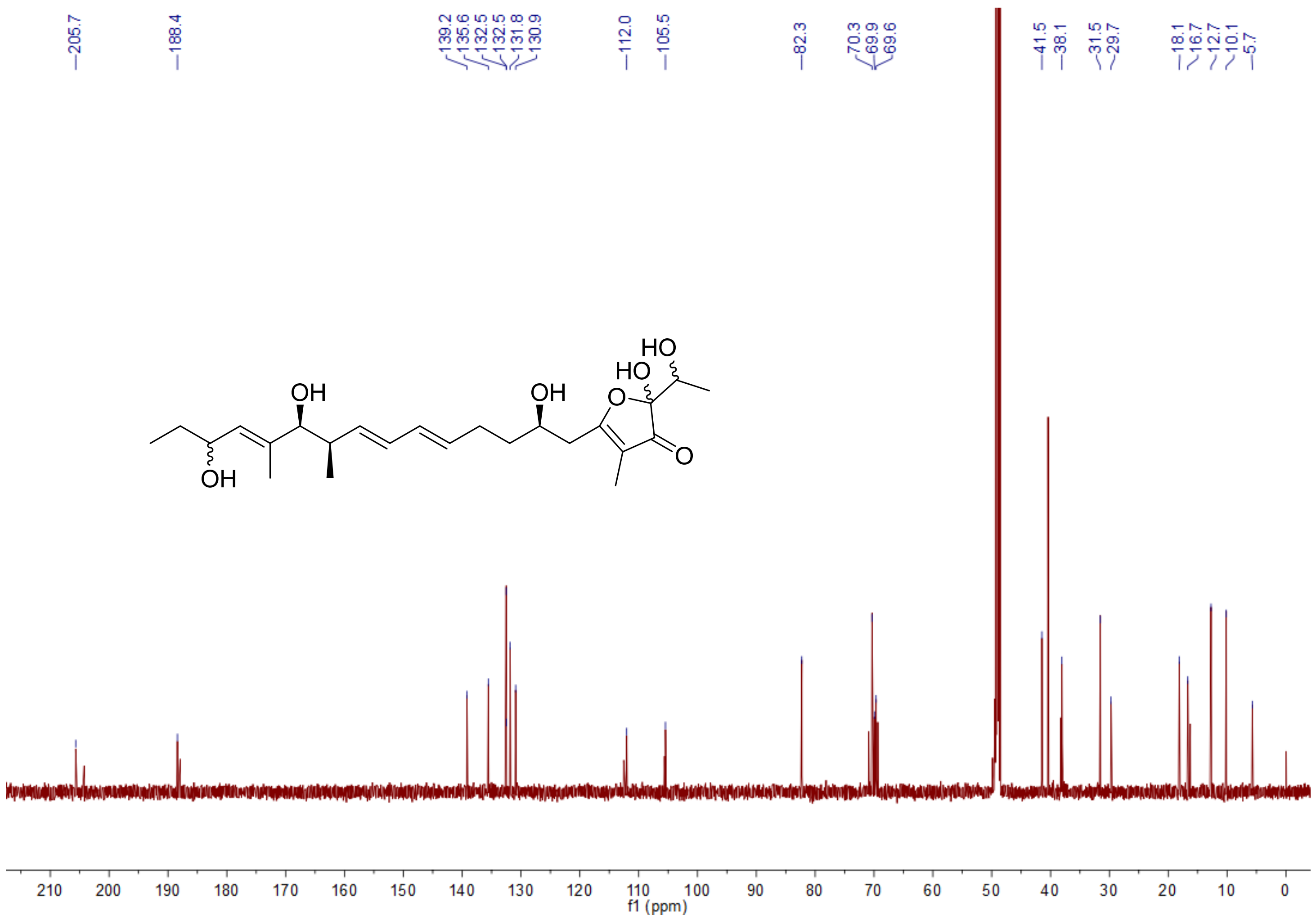

Figure S17. ^{13}C NMR (150 MHz, CD_3OD) spectrum of the new compound **2**

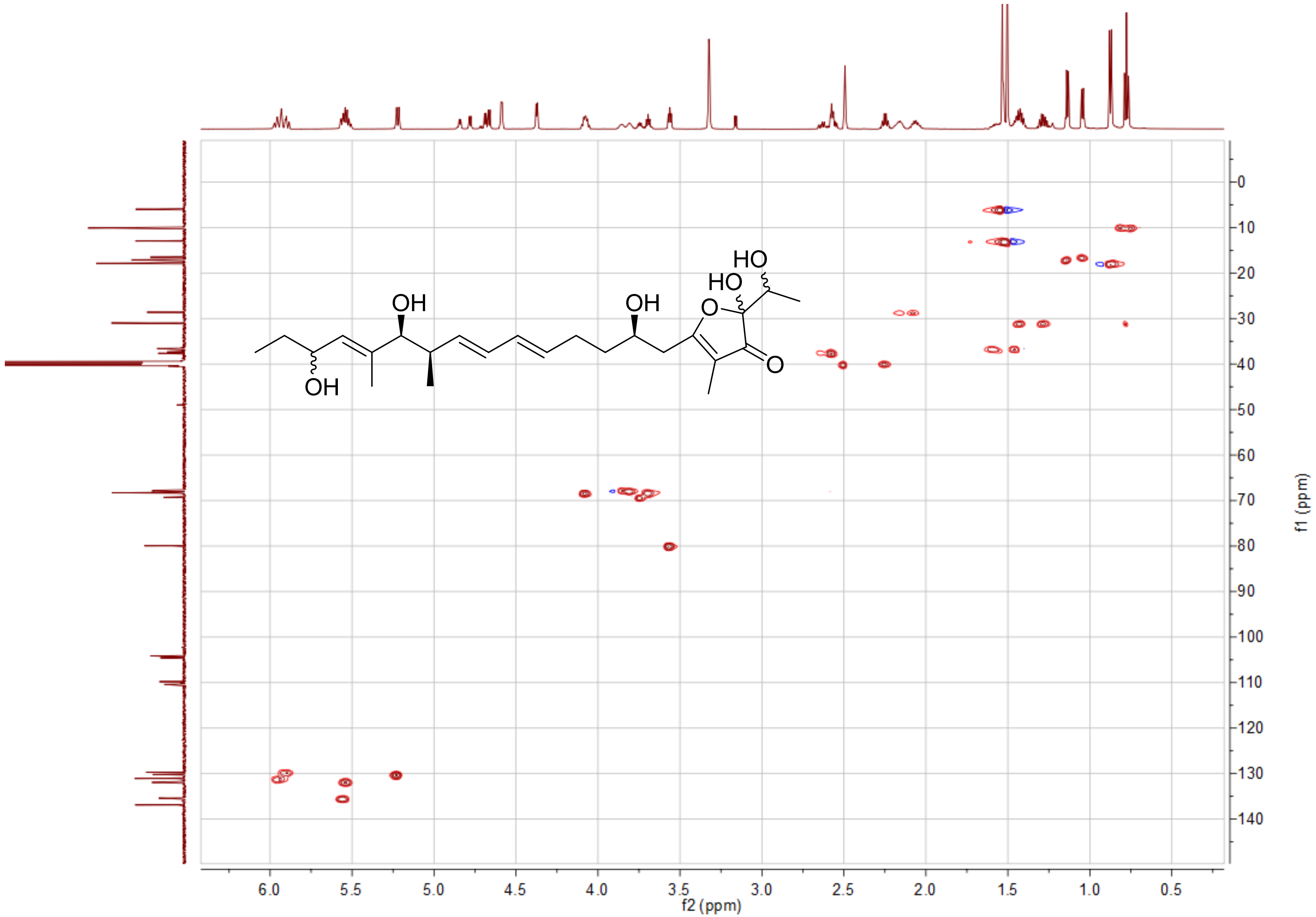

Figure S18. HSQC spectrum (DMSO-d_6) of the new compound **2**

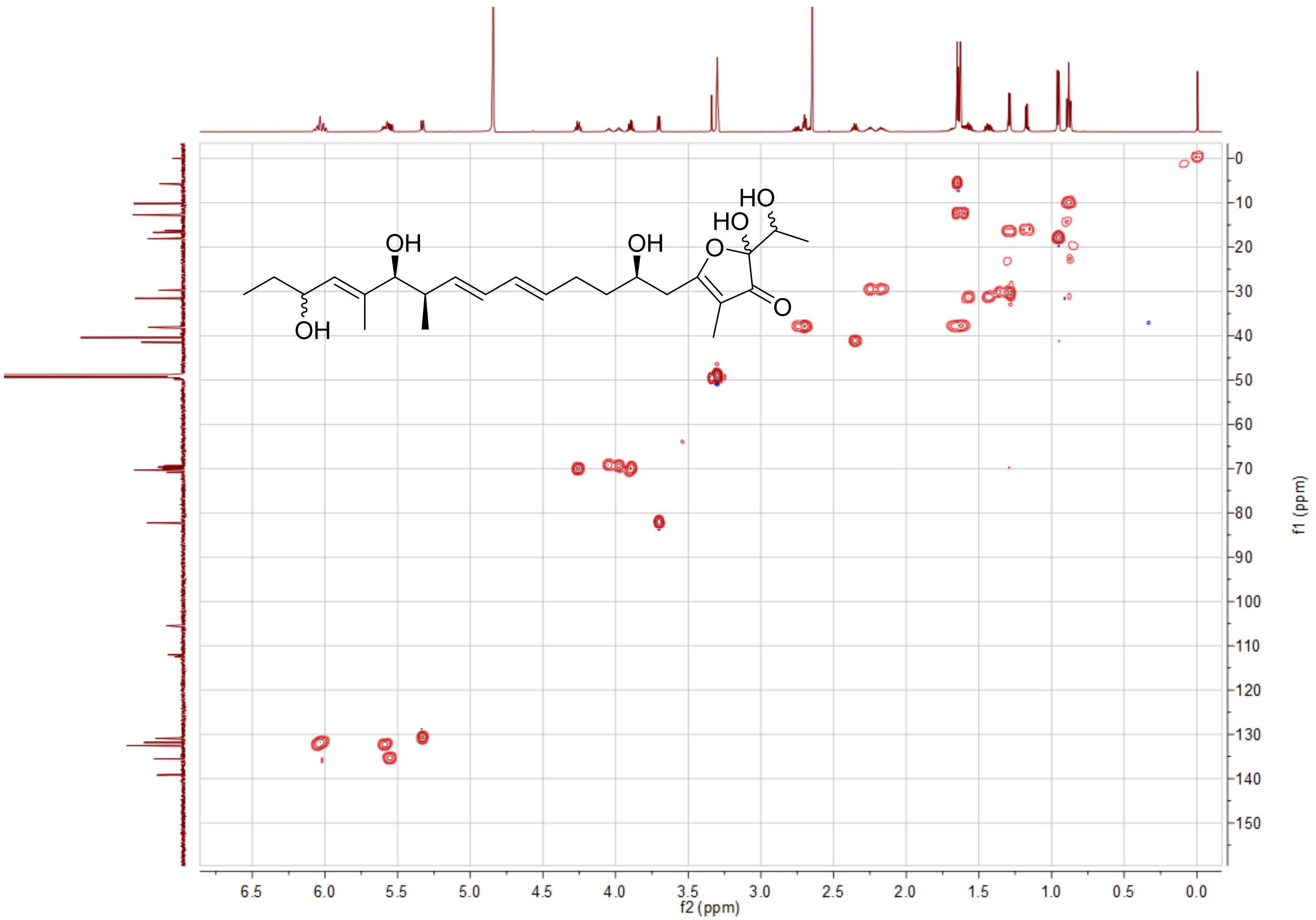

Figure S19. HSQC spectrum (CD_3OD) of the new compound **2**

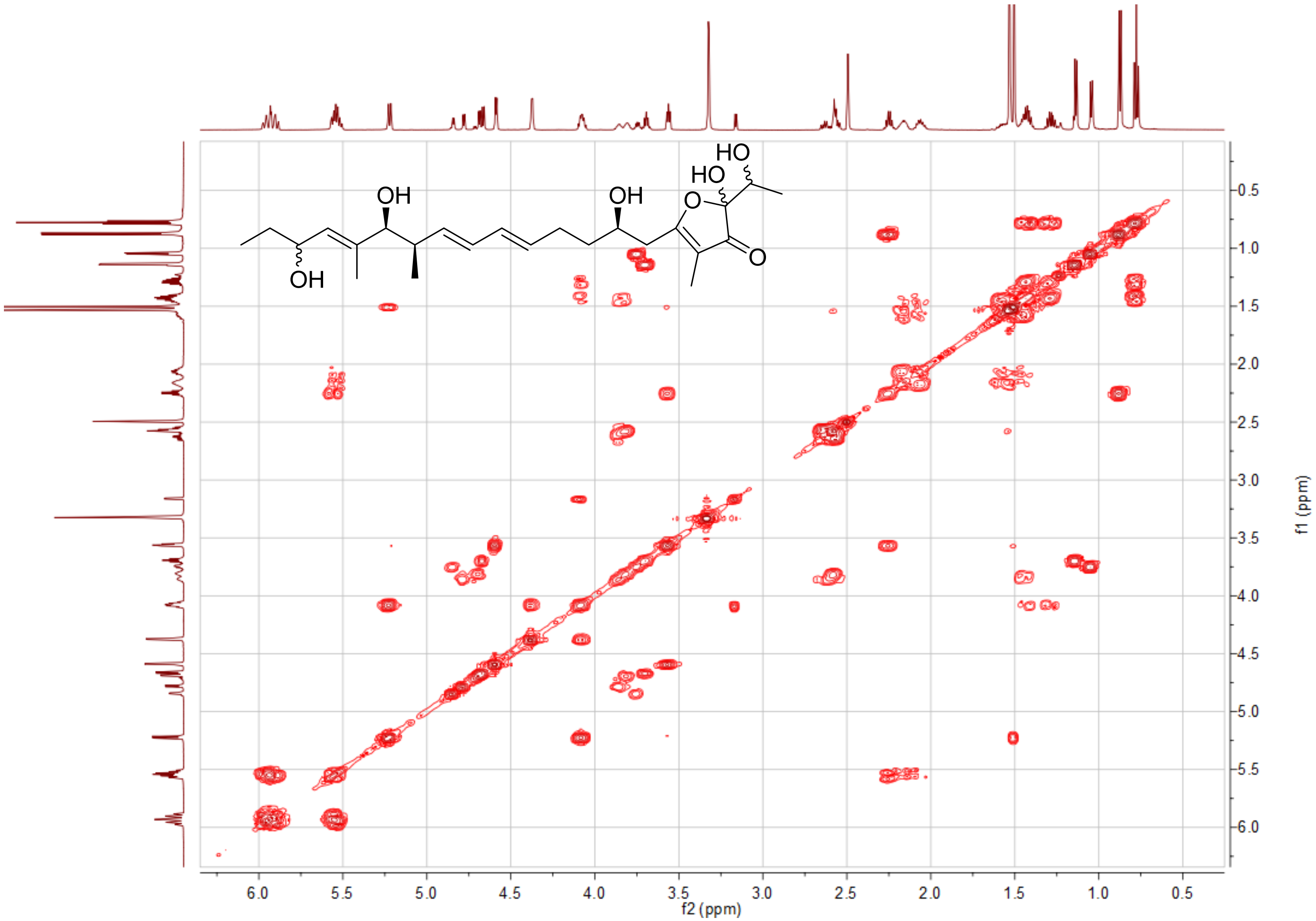

Figure S20. COSY spectrum (DMSO-d_6) of the new compound **2**

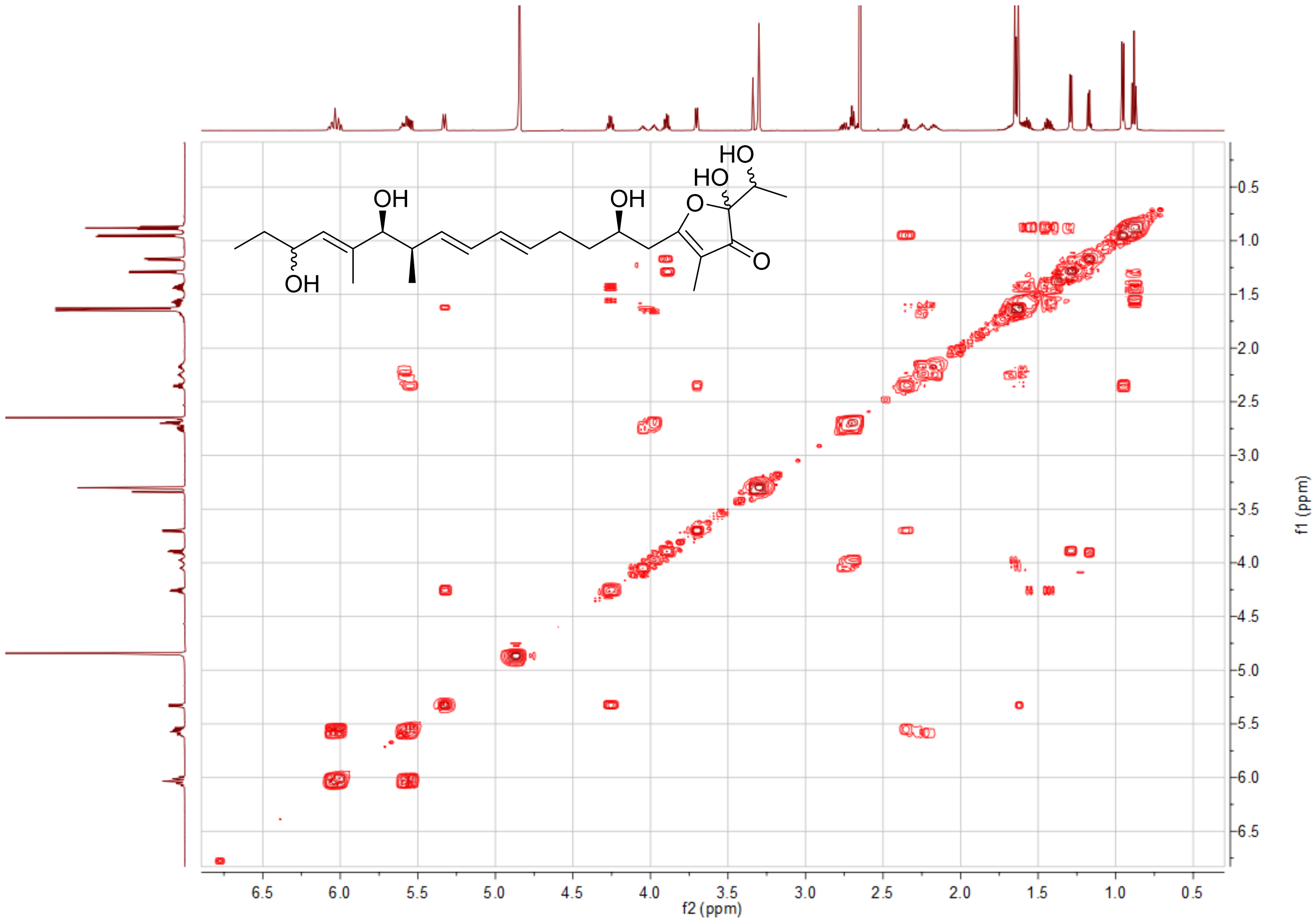

Figure S21. COSY spectrum (CD_3OD) of the new compound **2**

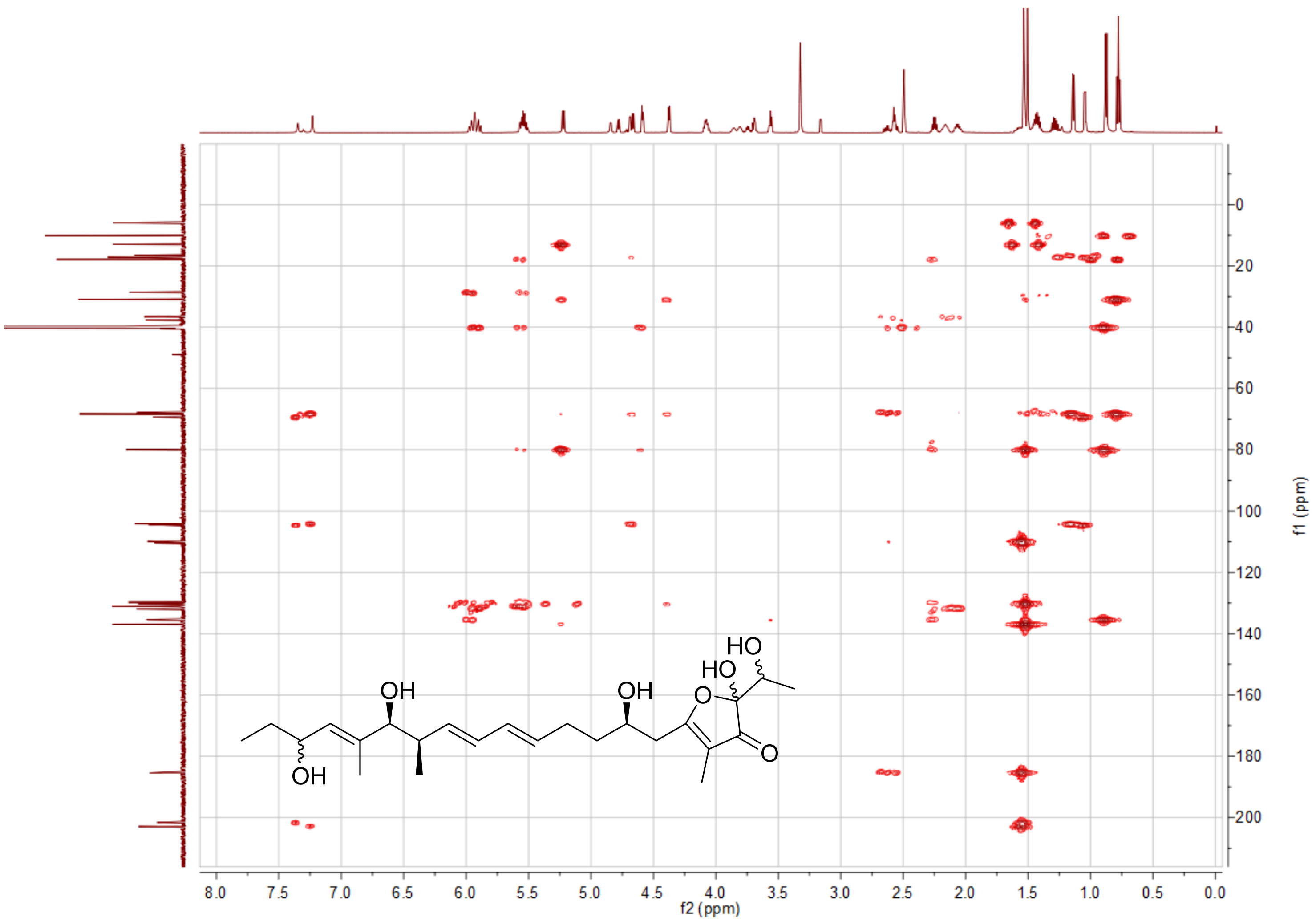

Figure S22. HMBC spectrum (DMSO-d_6) of the new compound **2**

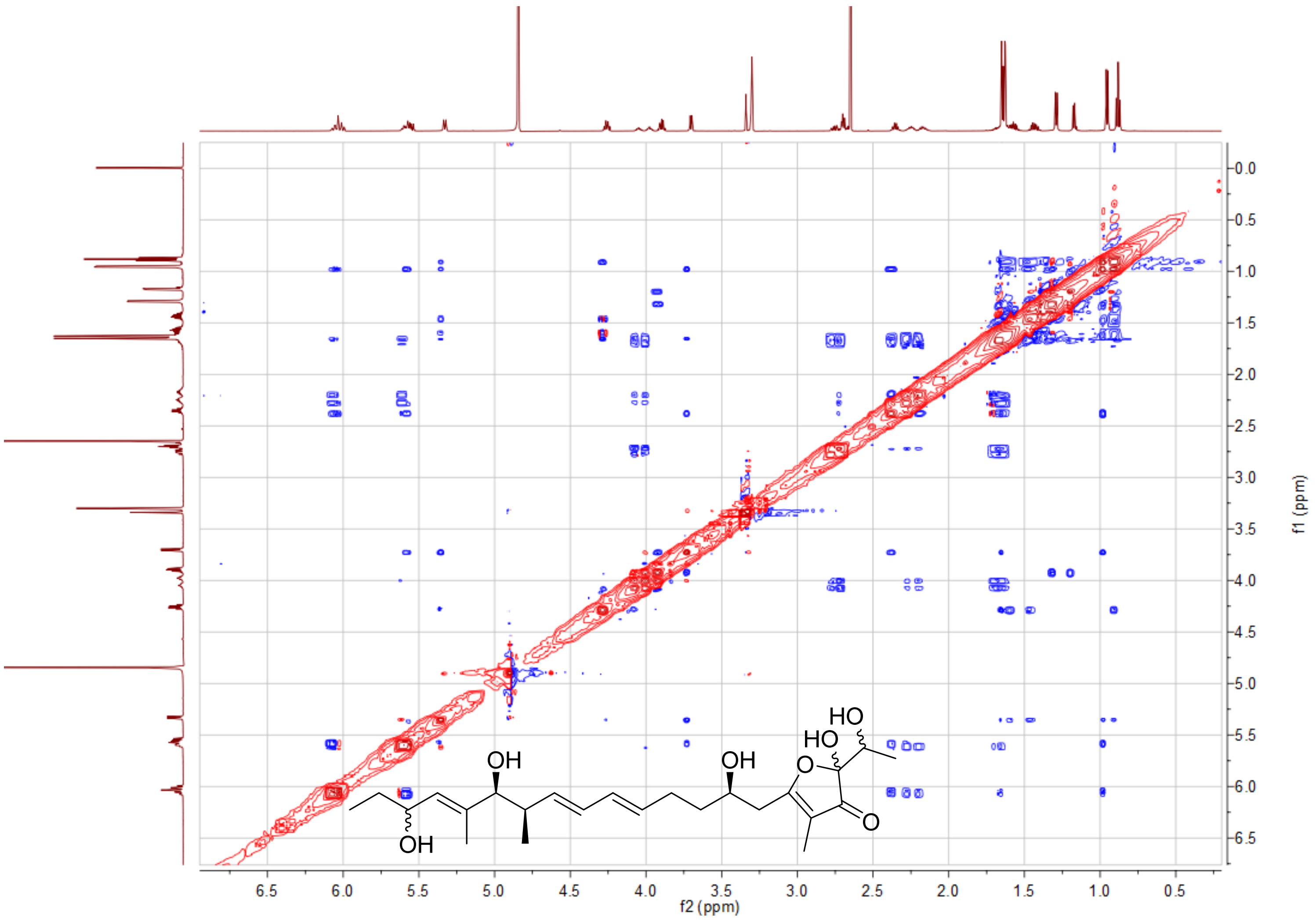

Figure S23. NOESY spectrum (CD_3OD) of the new compound **2**

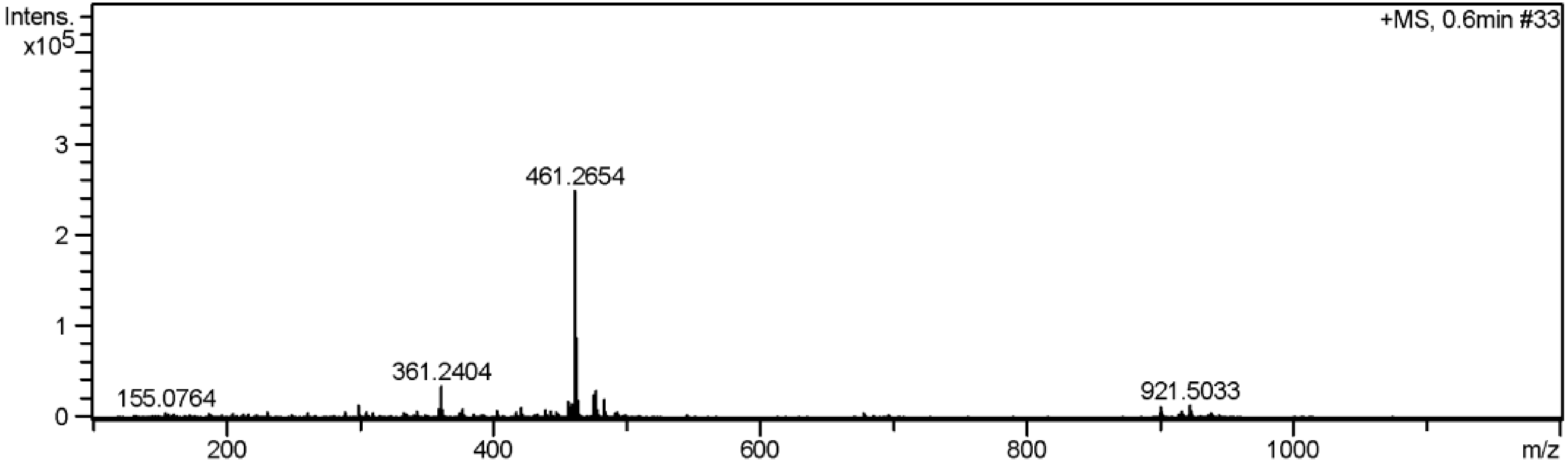

Figure S24. HRESI-MS spectrum of the new compound **3**

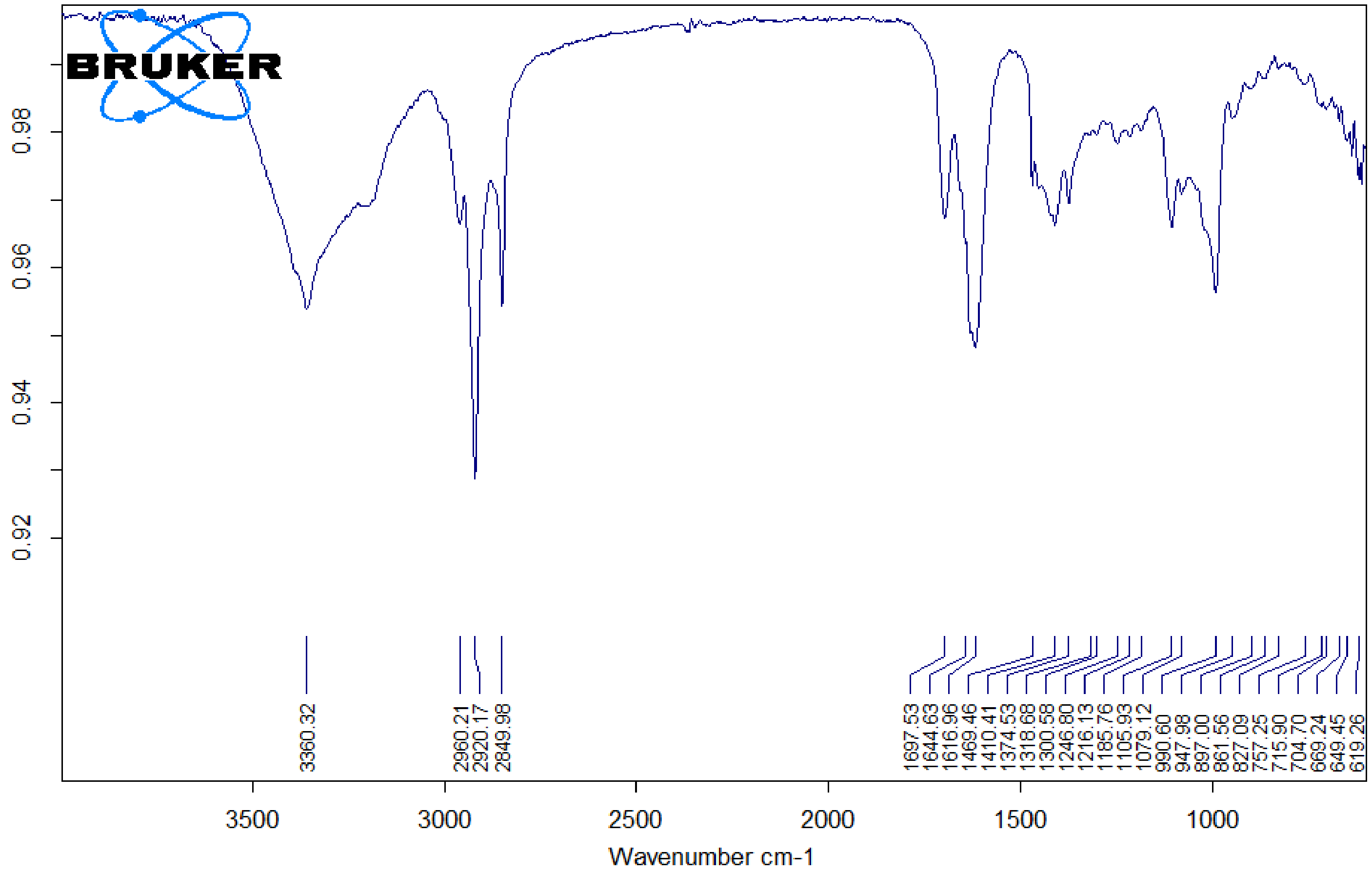

Figure S25. IR spectrum of the new compound **3**

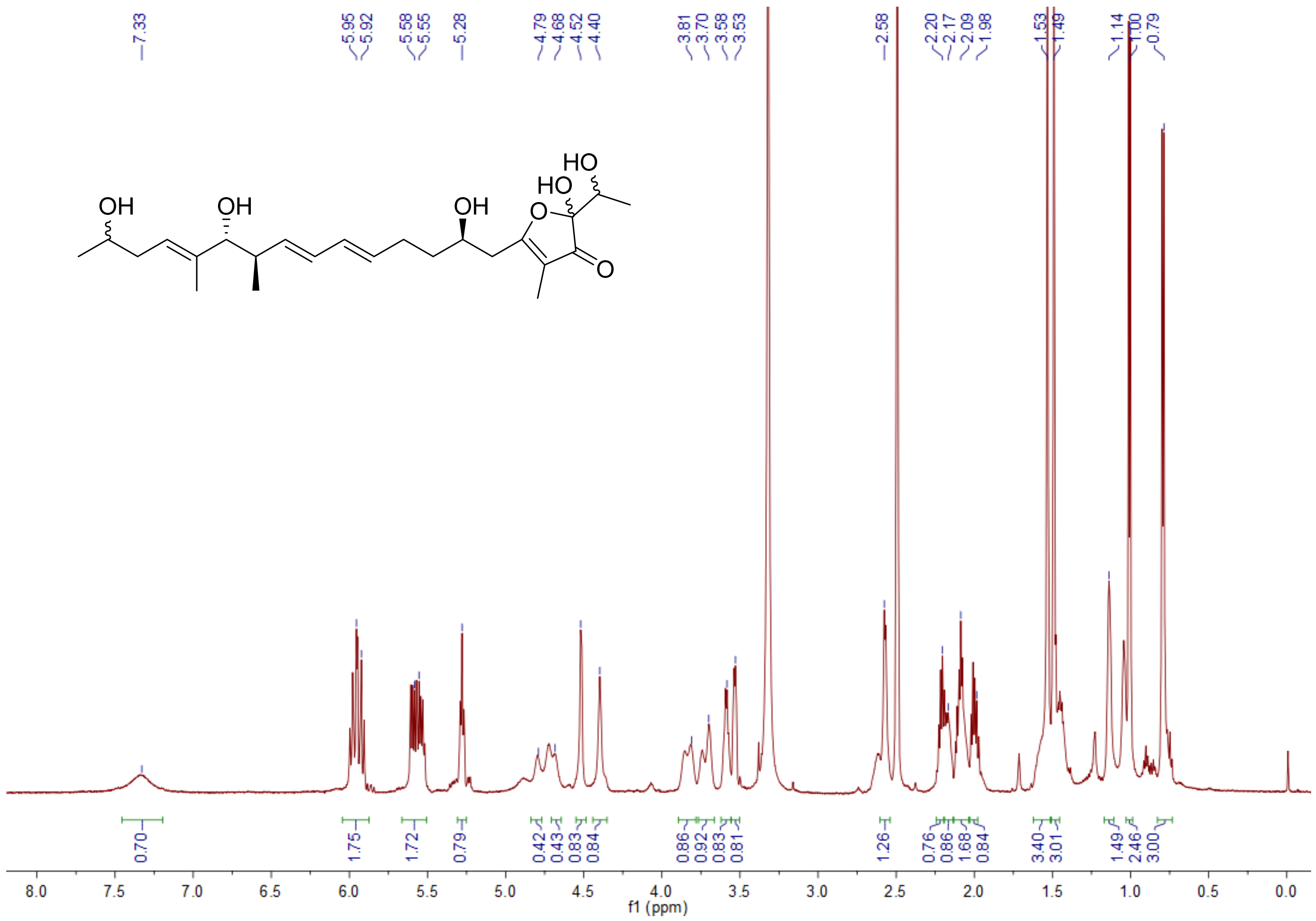

Figure S26. ^{1}H NMR (600 MHz, DMSO-d_6) spectrum of the new compound **3**

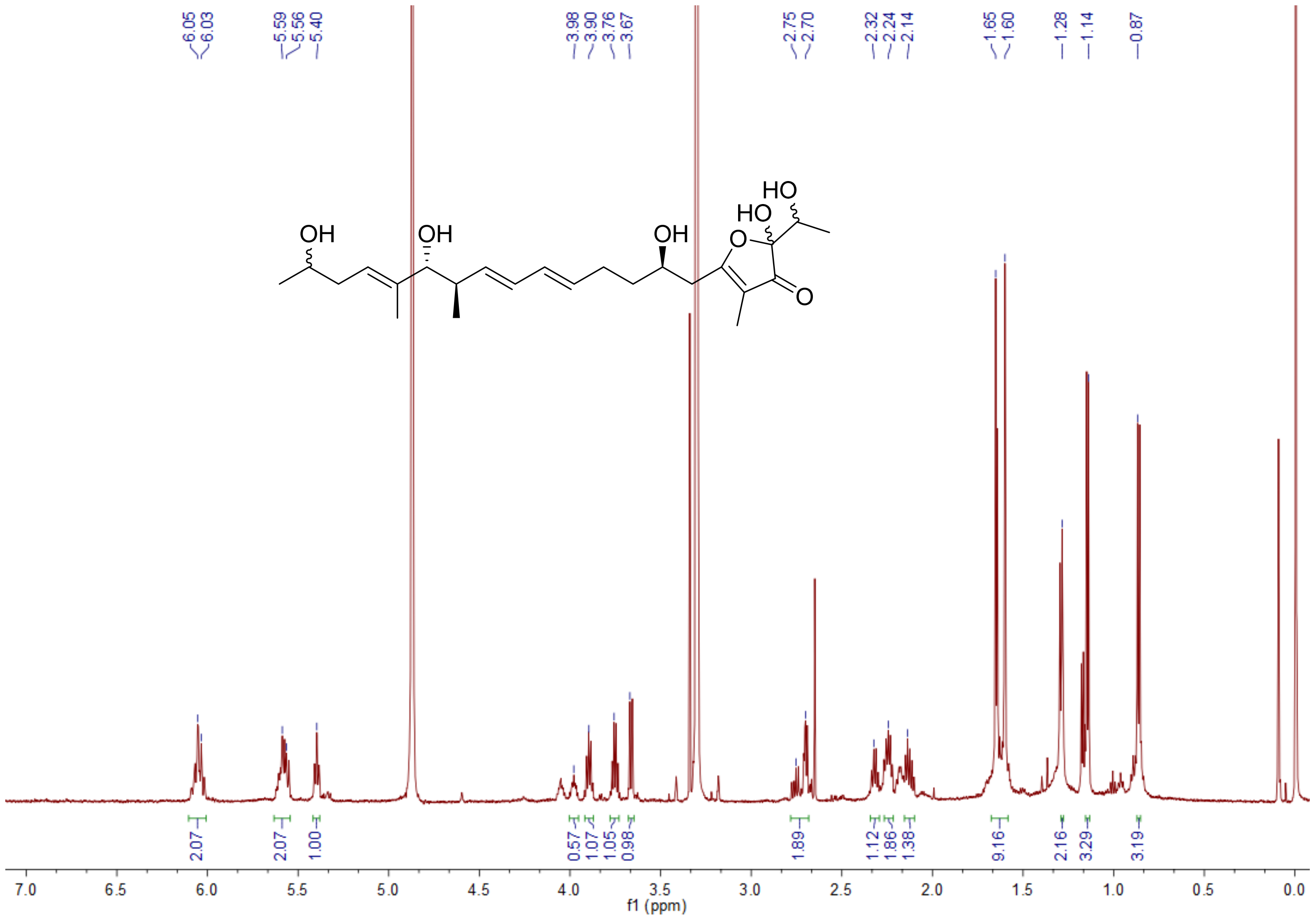

Figure S27. ^1H NMR (600 MHz, CD_3OD) spectrum of the new compound **3**

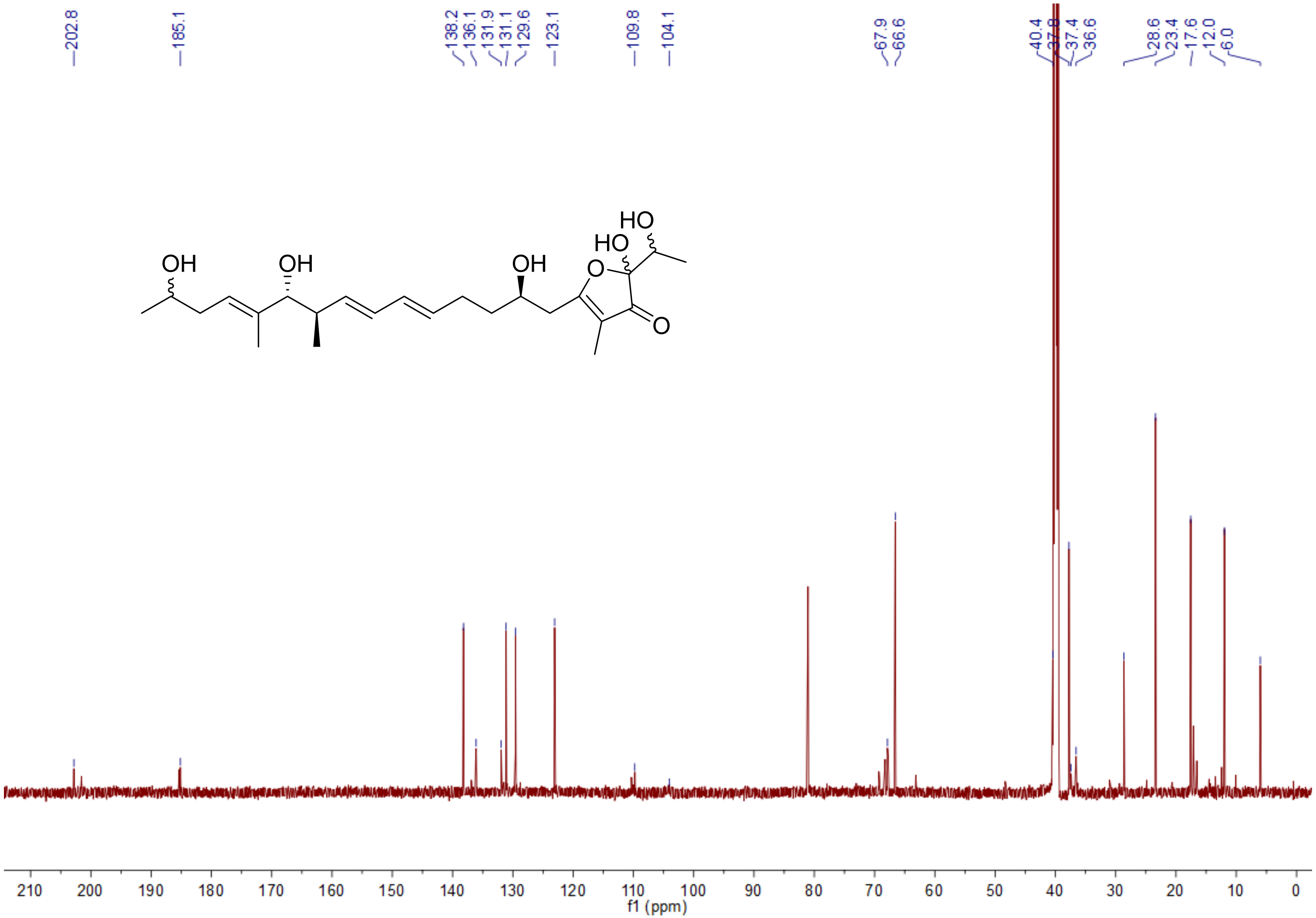

Figure S28. ^{13}C NMR (150 MHz, DMSO-d_6) spectrum of the new compound **3**

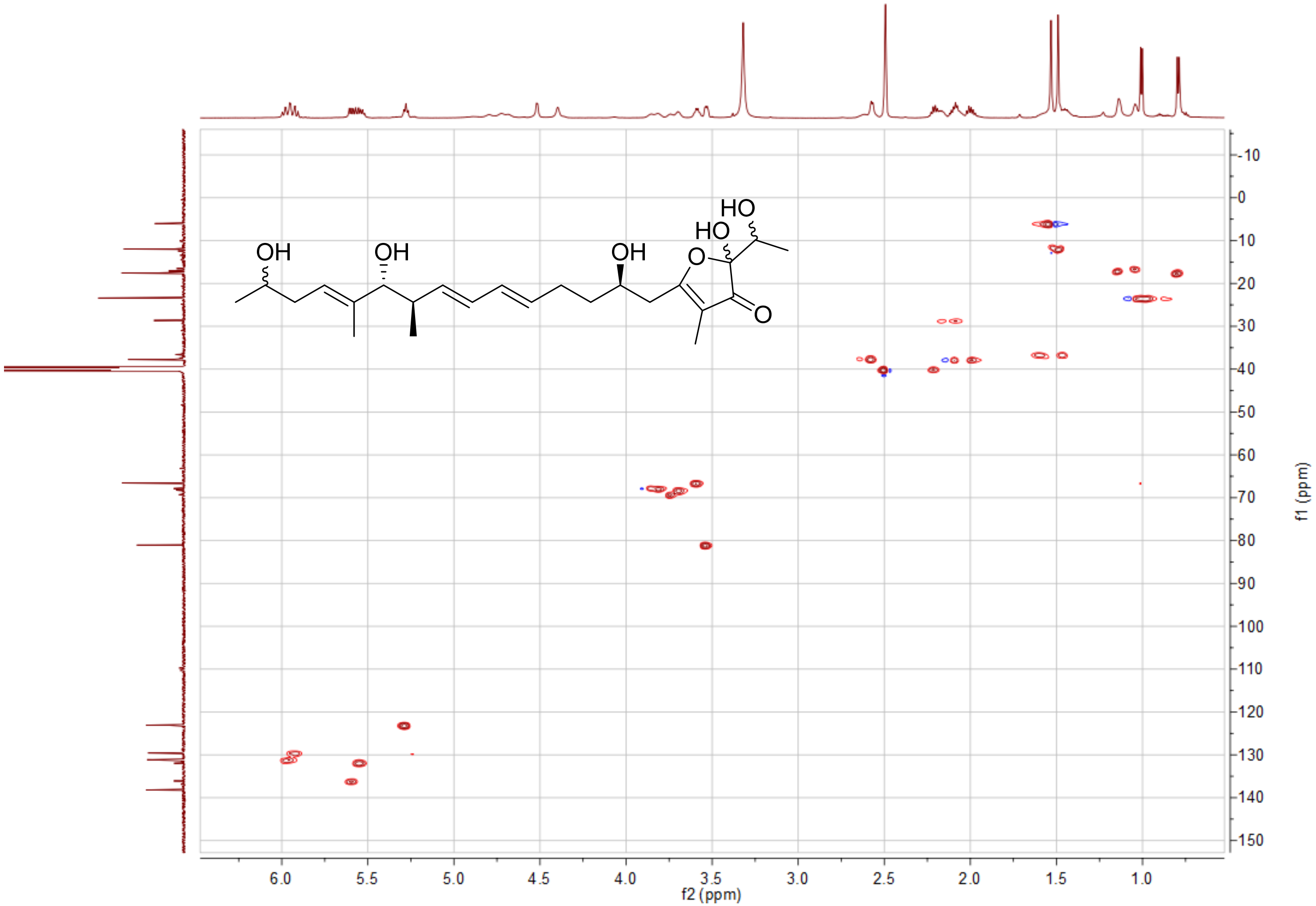

Figure S29. HSQC spectrum of the new compound **3**

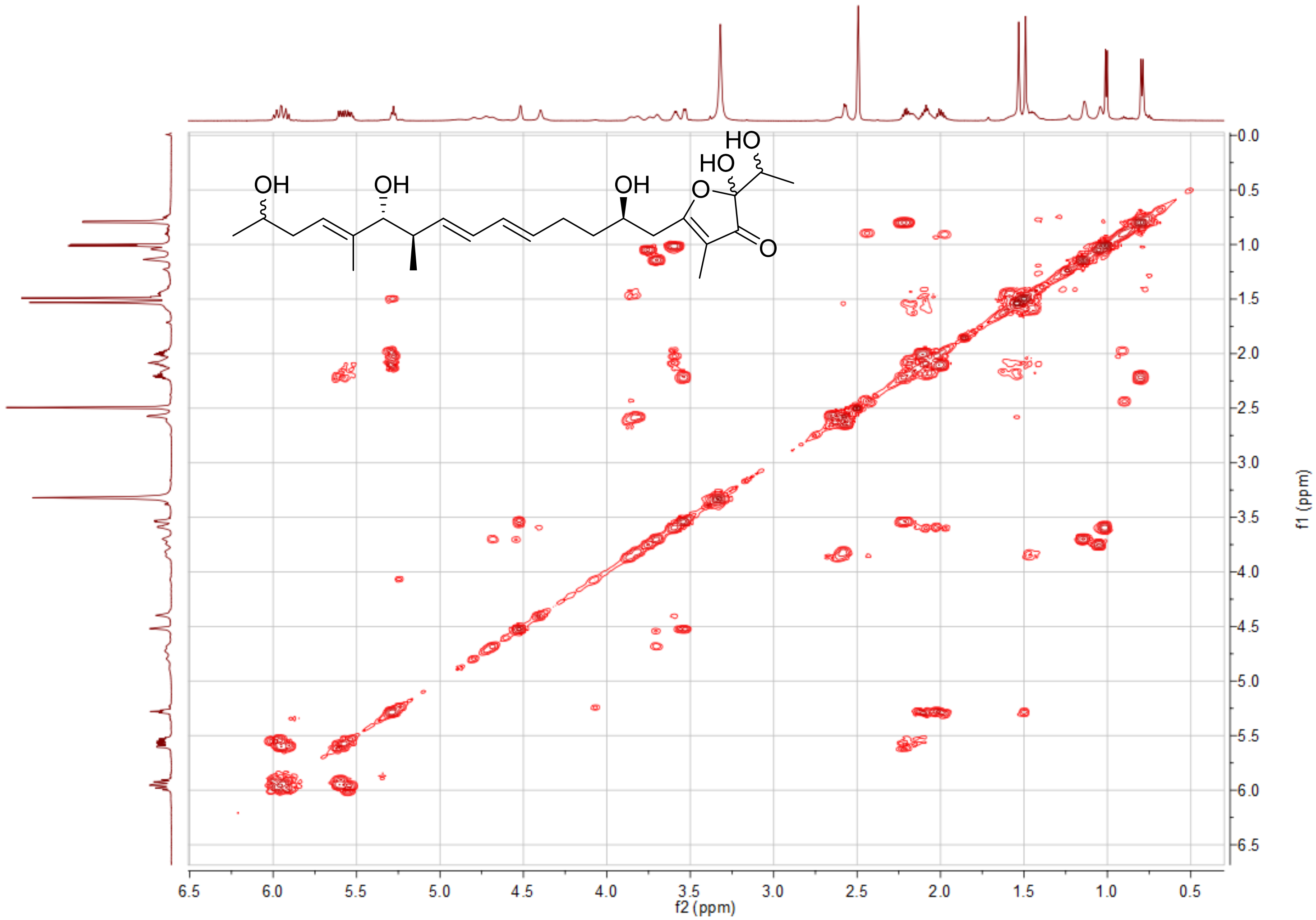

Figure S30. COSY spectrum (DMSO-d_6) of the new compound **3**

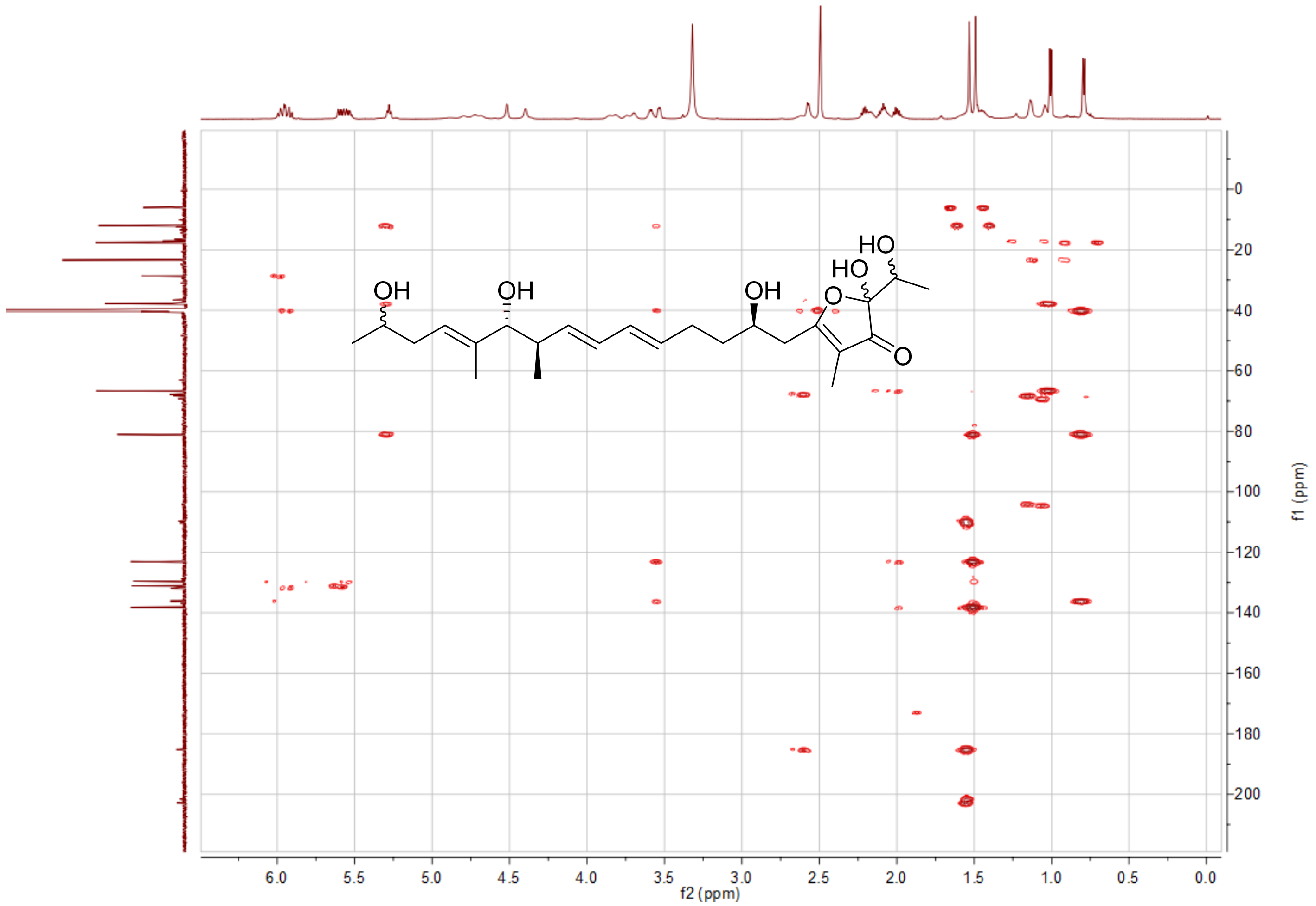

Figure S31. HMBC spectrum (DMSO-d_6) of the new compound **3**

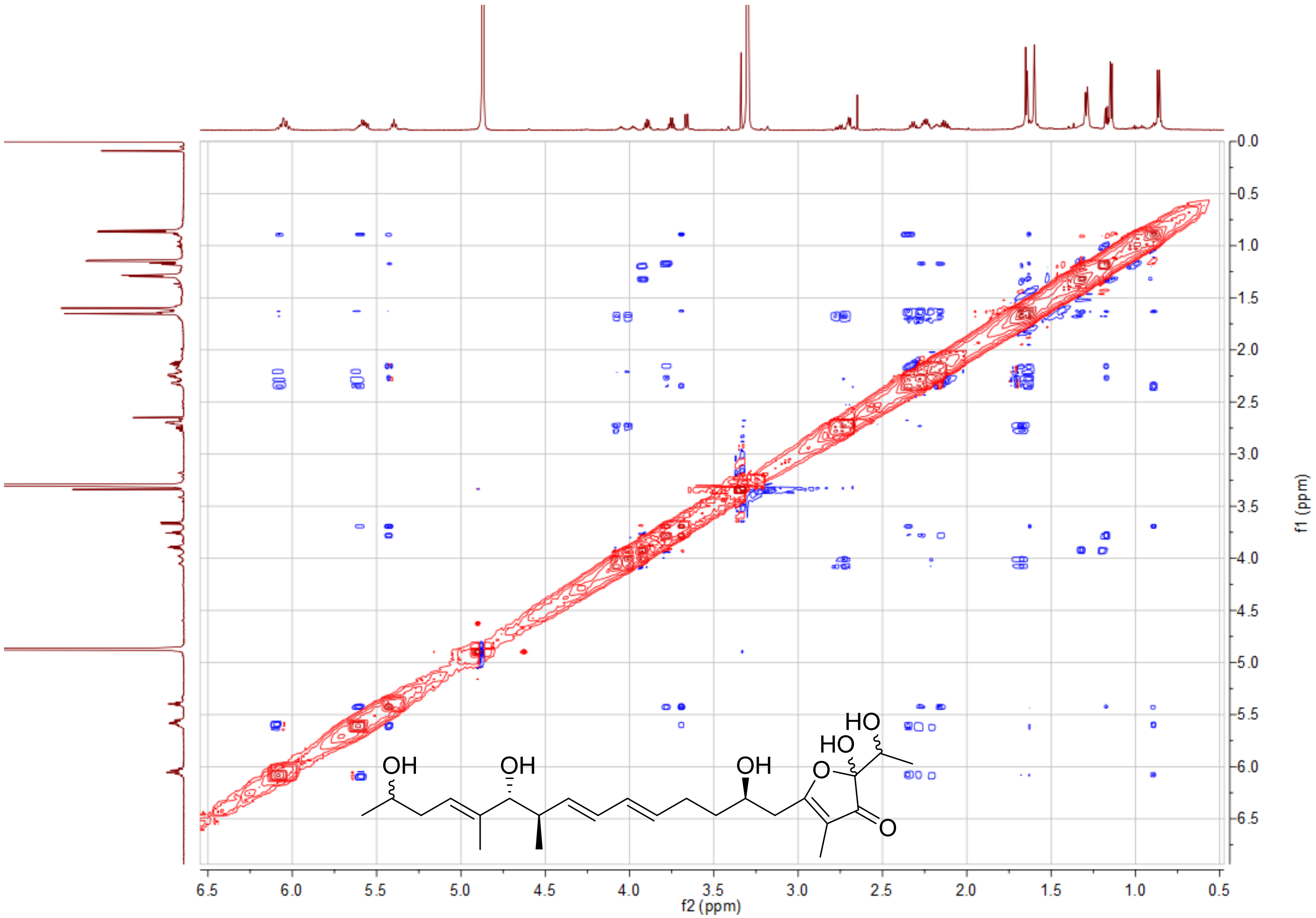

Figure S32. NOESY spectrum (CD_3OD) of the new compound **3**

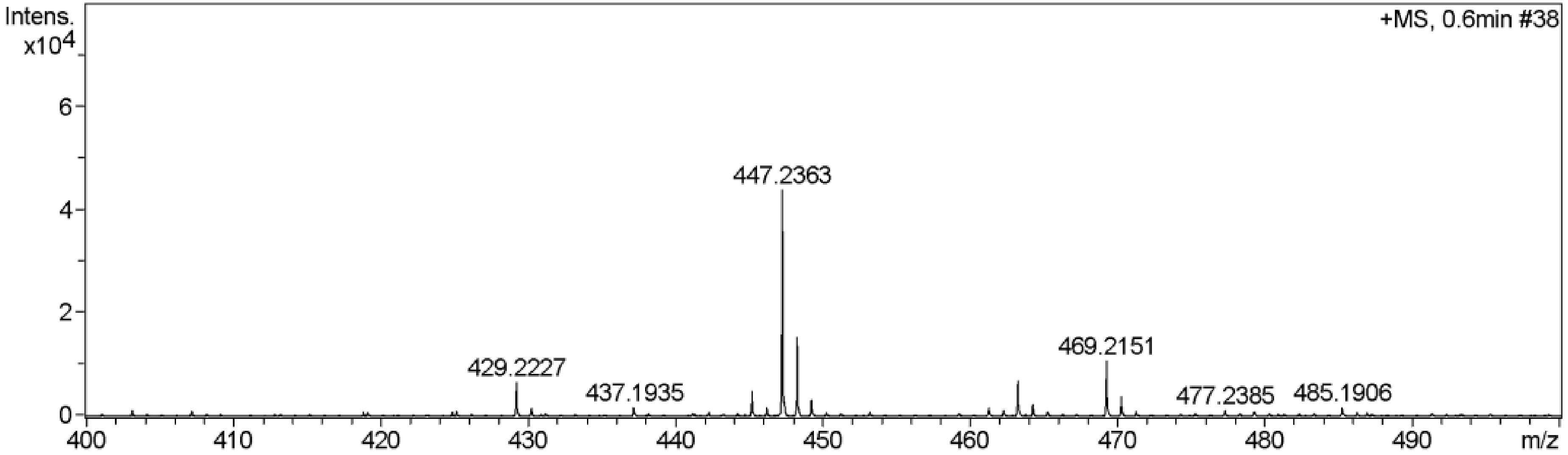

Figure S33. HRESI-MS spectrum of the new compound **4**

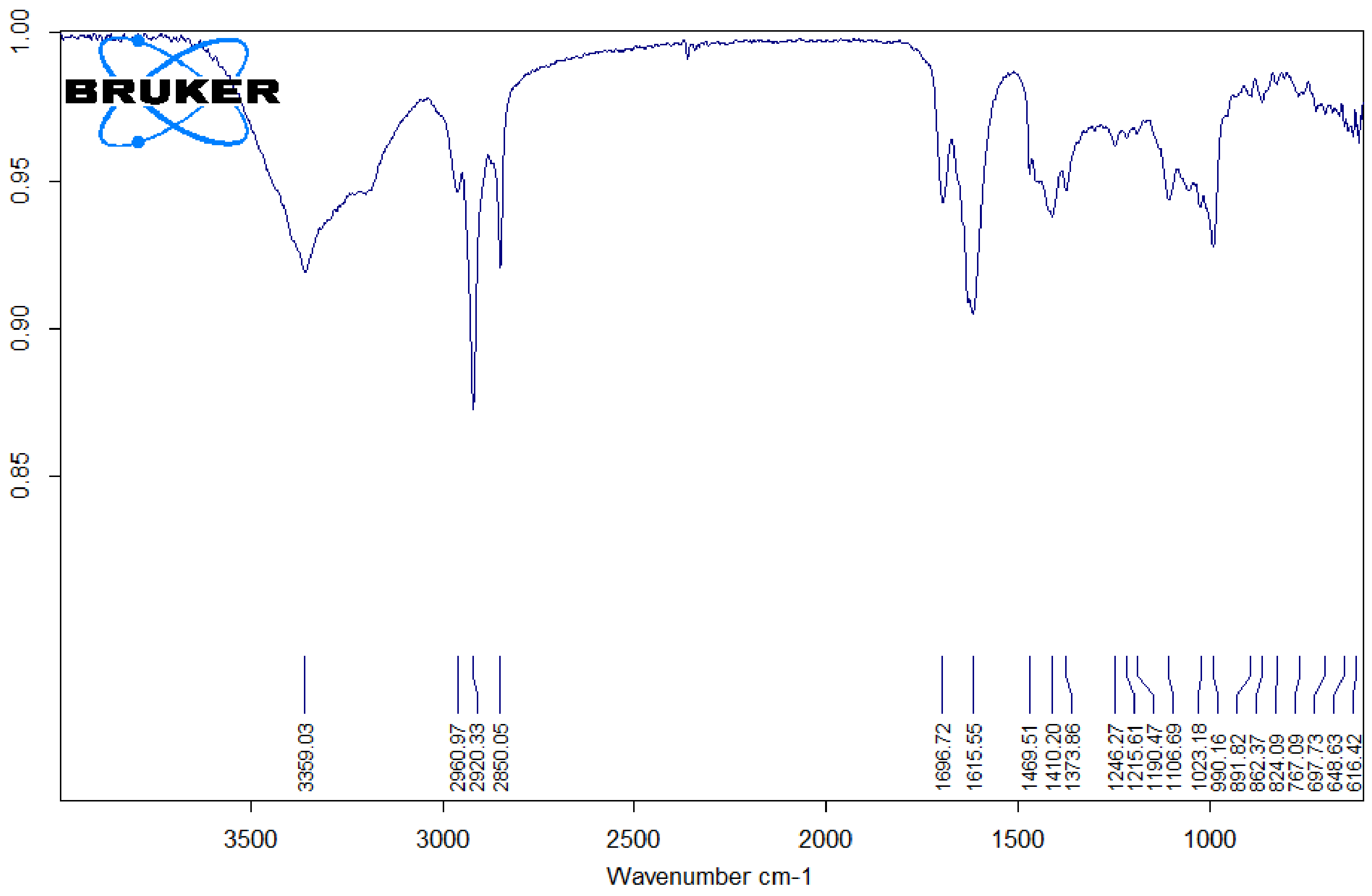

Figure S34. IR spectrum of the new compound **4**

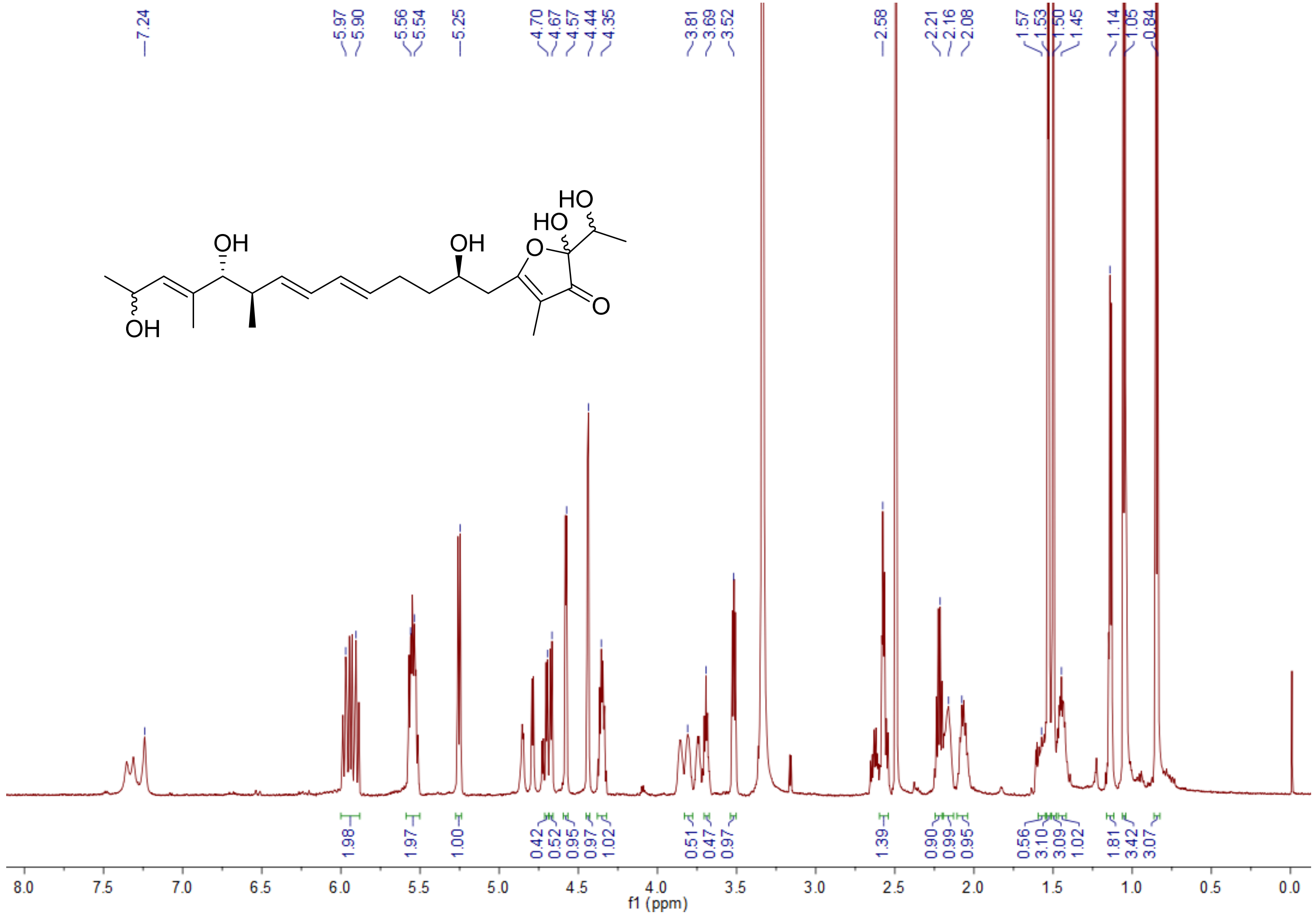

Figure S35. ^{1}H NMR (600 MHz, DMSO-d_6) spectrum of the new compound **4**

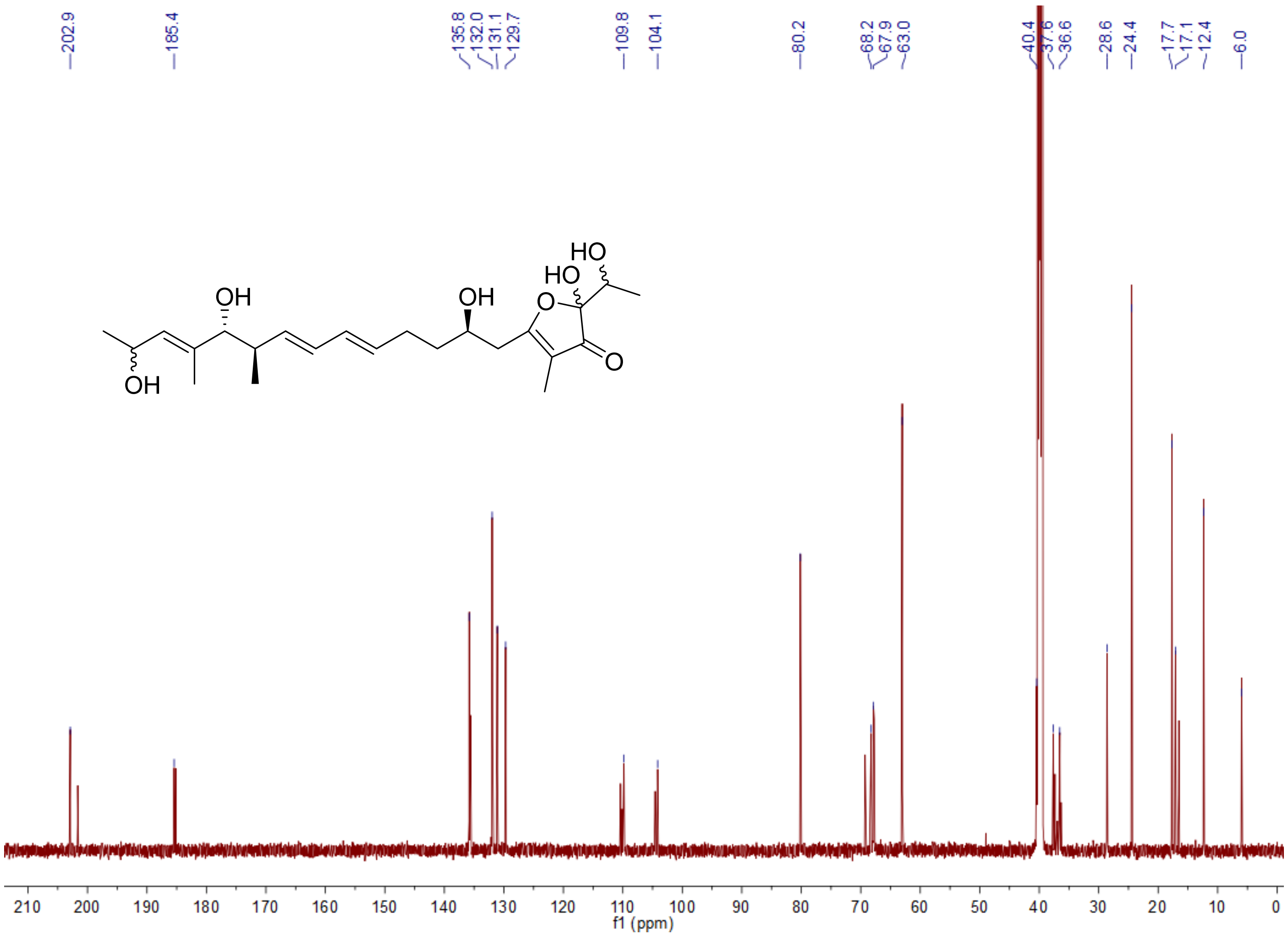

Figure S36. ^{13}C NMR (150 MHz, DMSO-d_6) spectrum of the new compound **4**

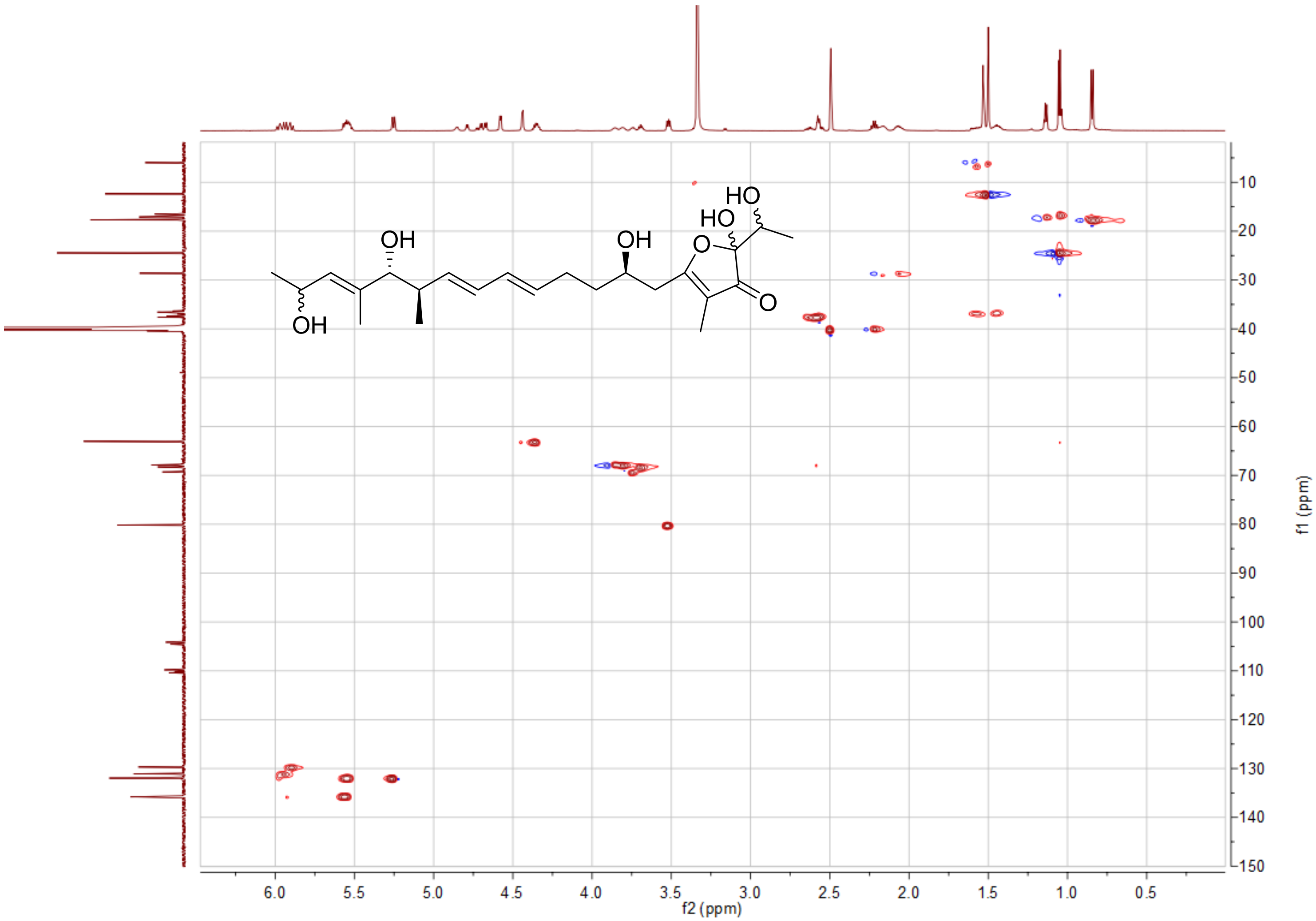

Figure S37. HSQC spectrum of the new compound **4**

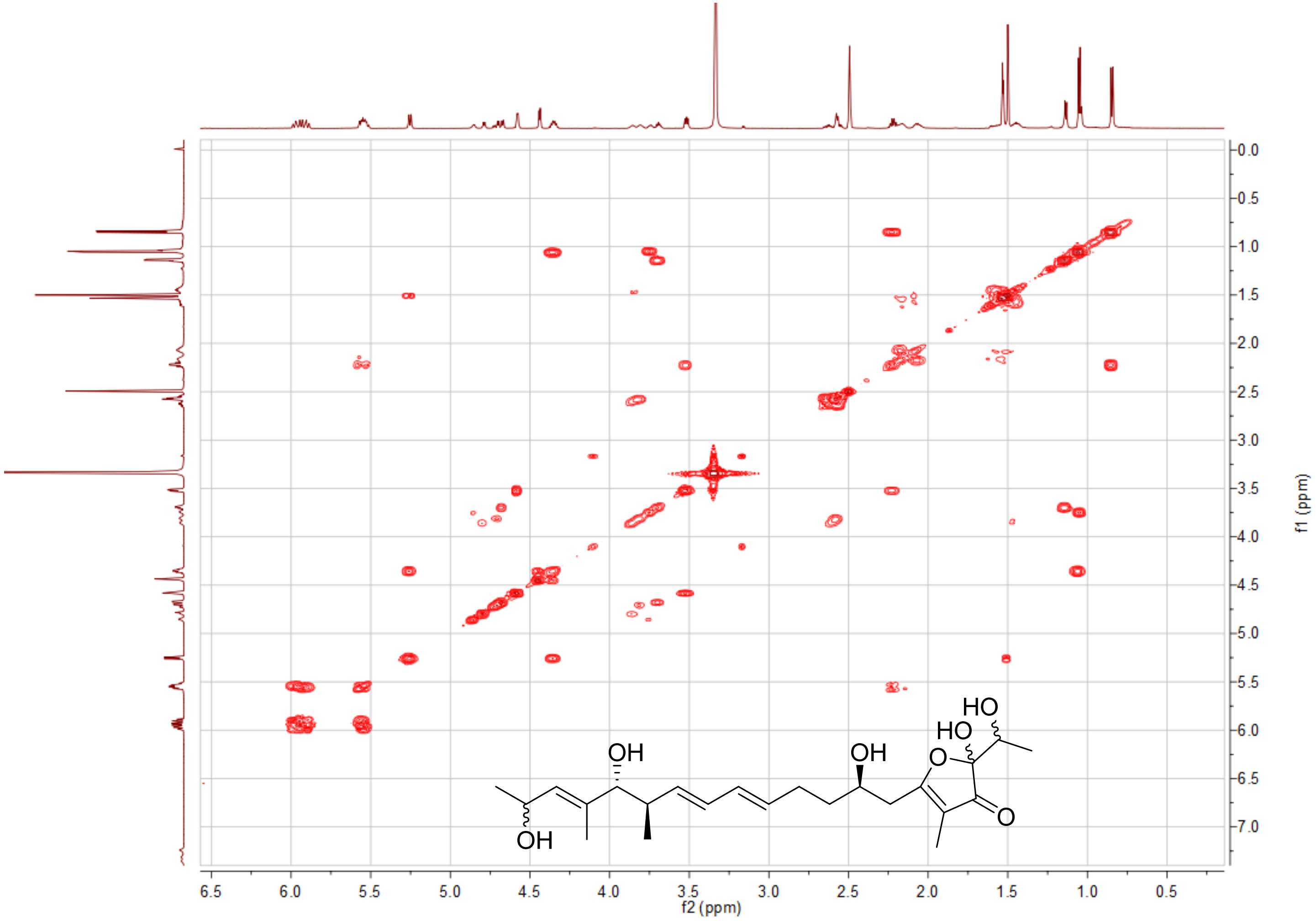

Figure S38. COSY spectrum of the new compound **4**

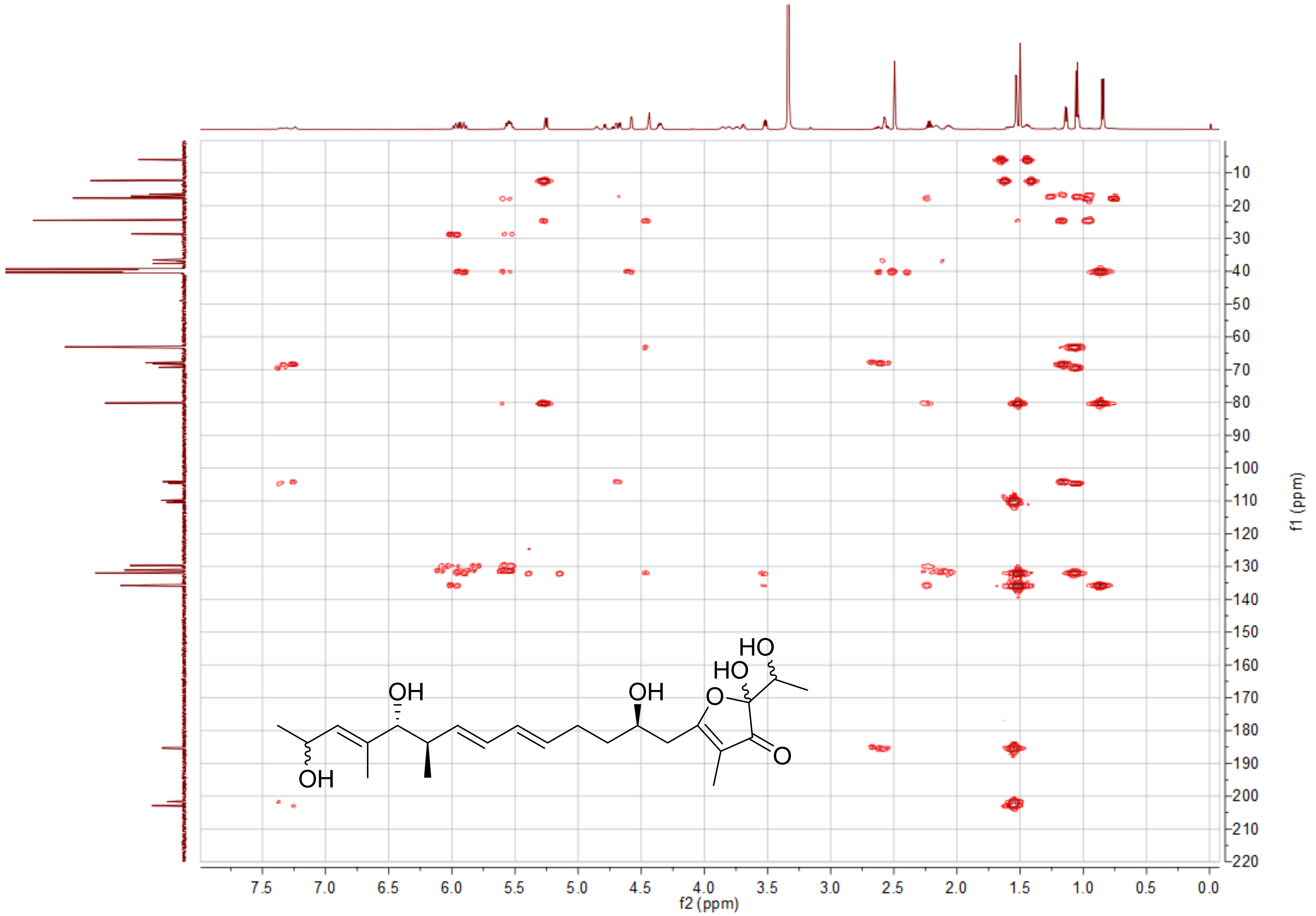

Figure S39. HMBC spectrum of the new compound **4**

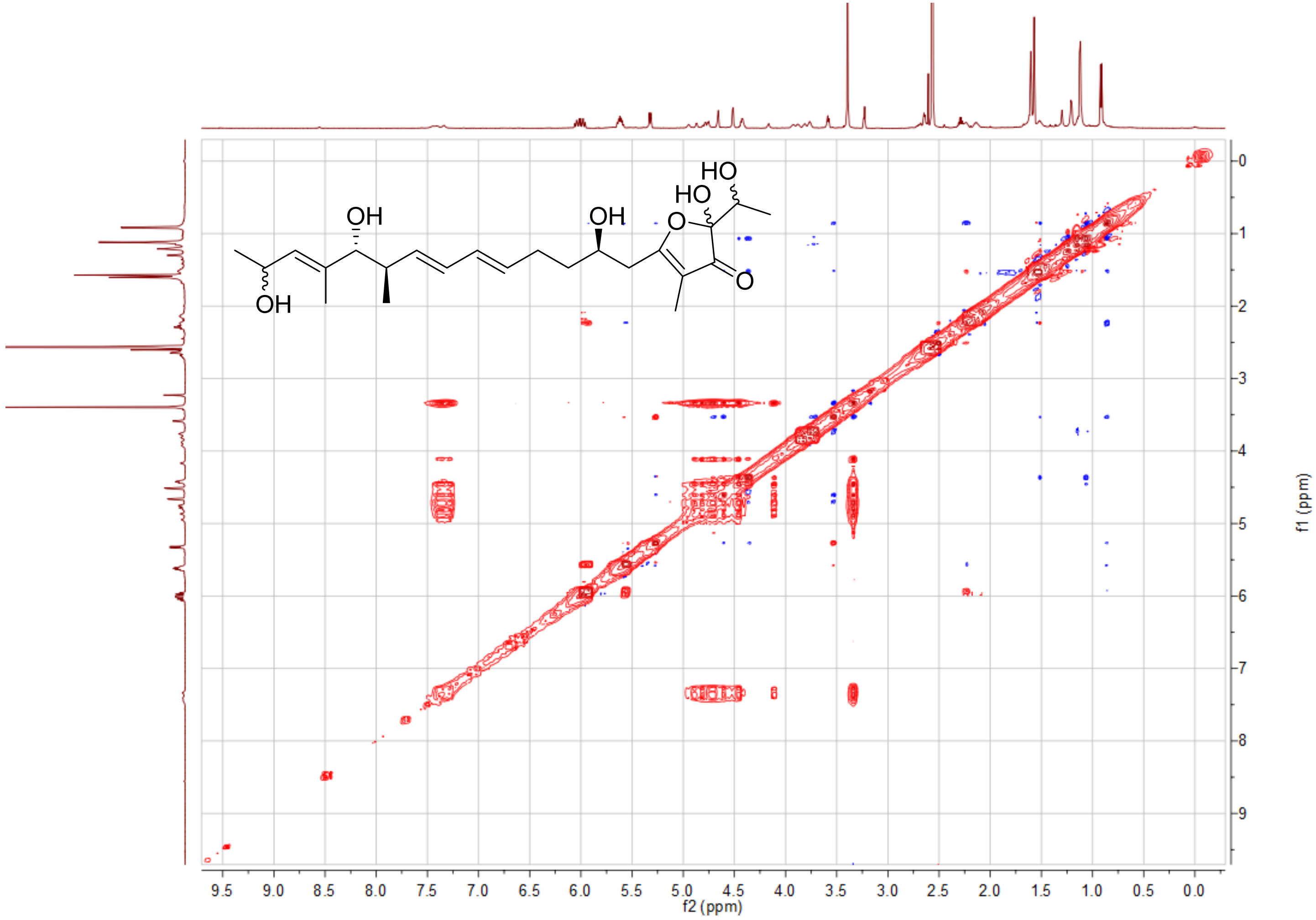

Figure S40. NOESY spectrum of the new compound **4**

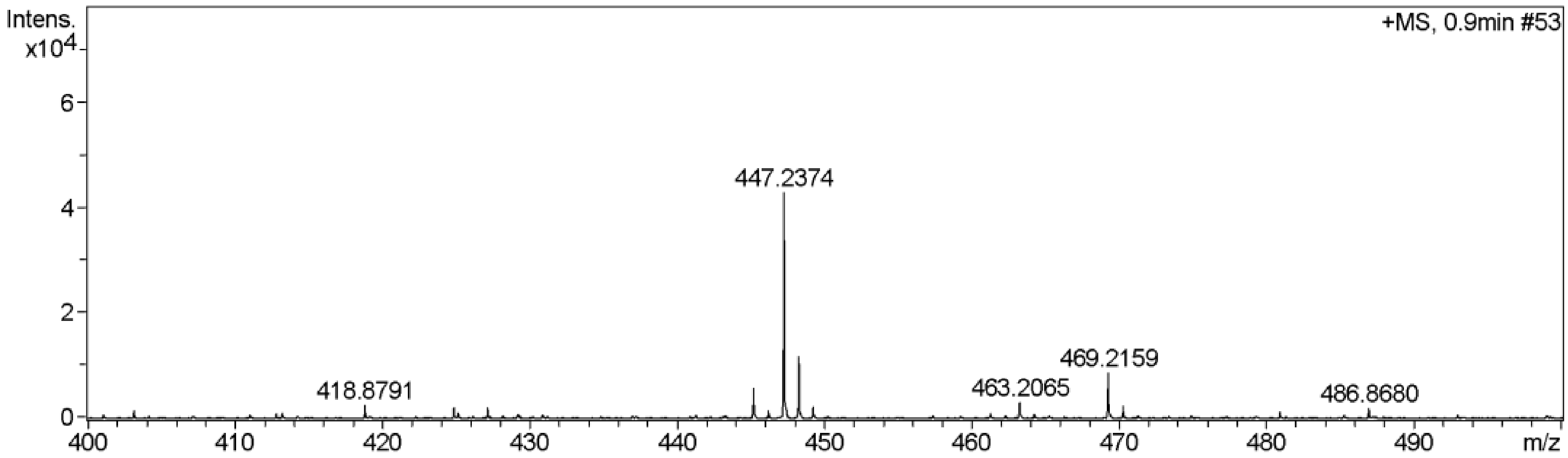

Figure S41. HRESI-MS spectrum of the new compound **5**

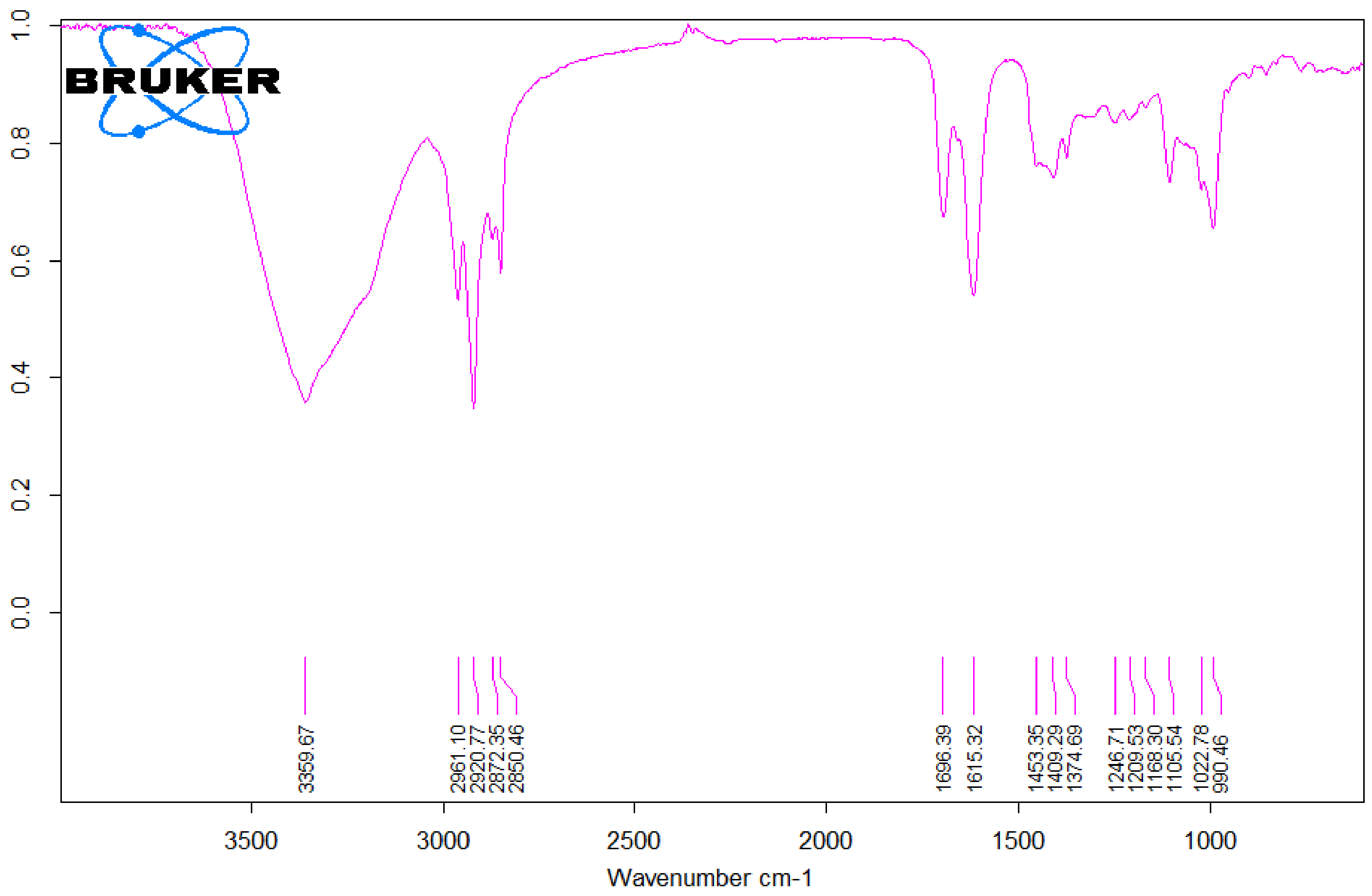

Figure S42. IR spectrum of the new compound **5**

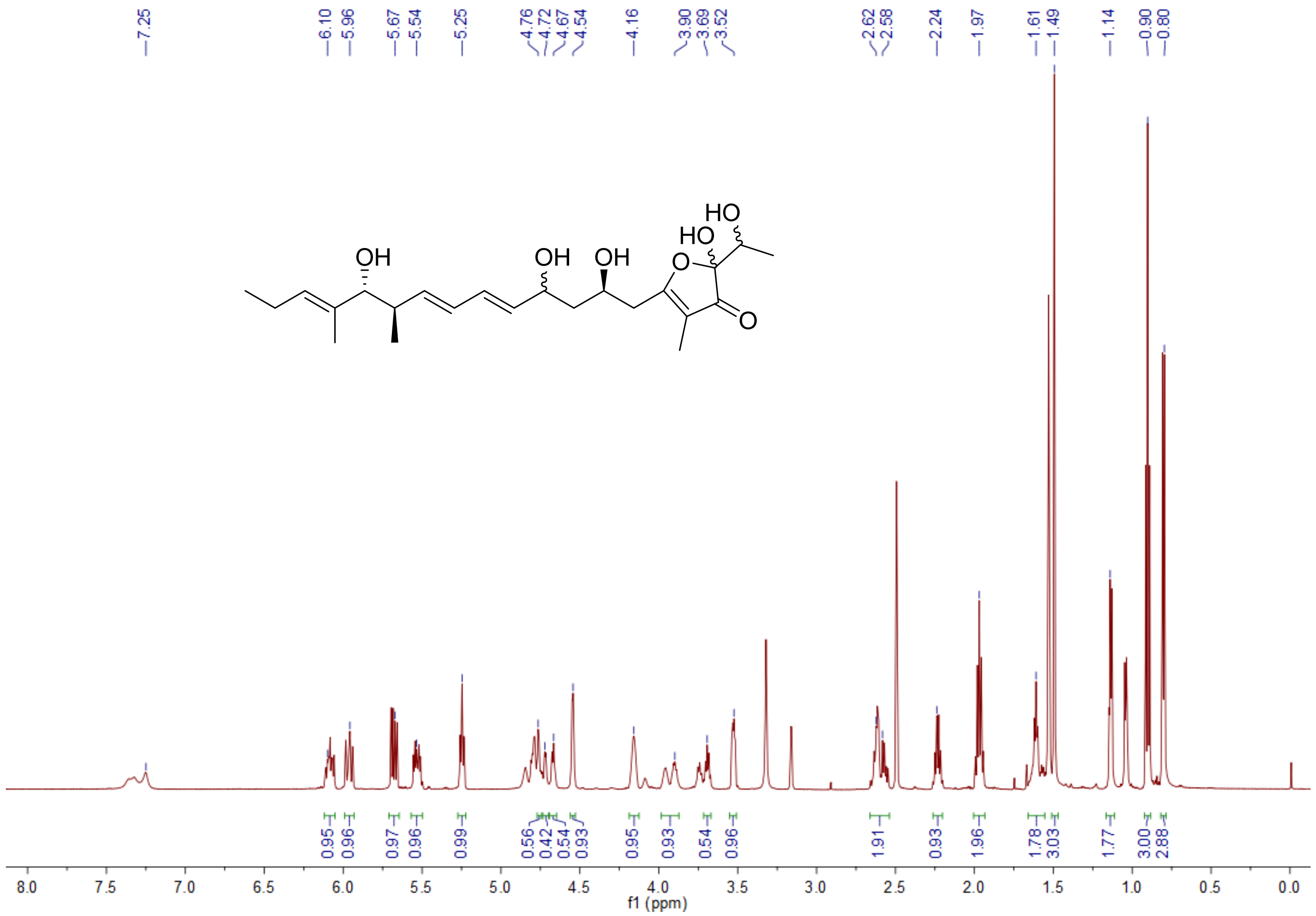

Figure S43. ^{1}H NMR (600 MHz, DMSO-d_6) spectrum of the new compound **5**

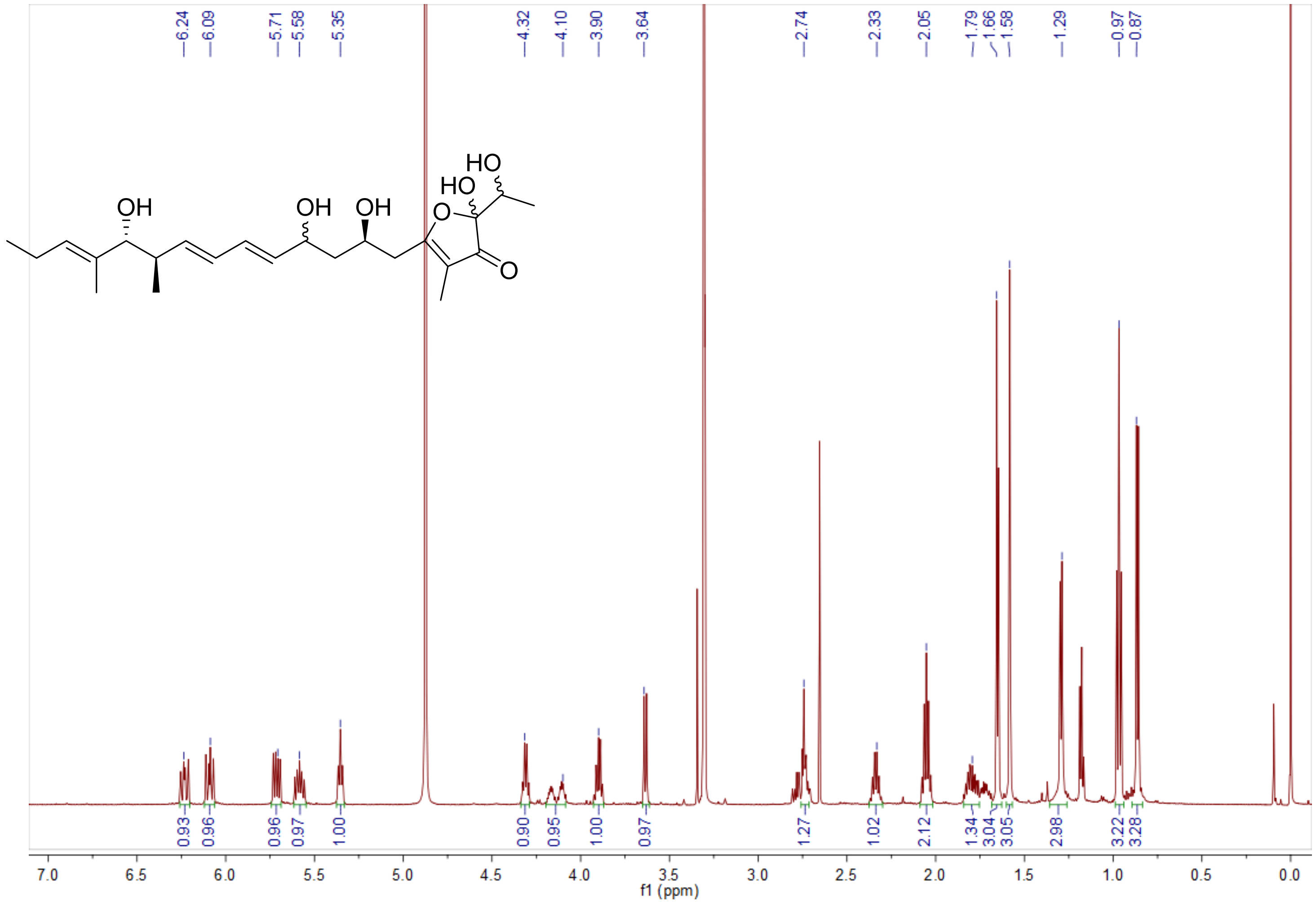

Figure S44. ^{1}H NMR (600 MHz, CD_3OD) spectrum of the new compound **5**

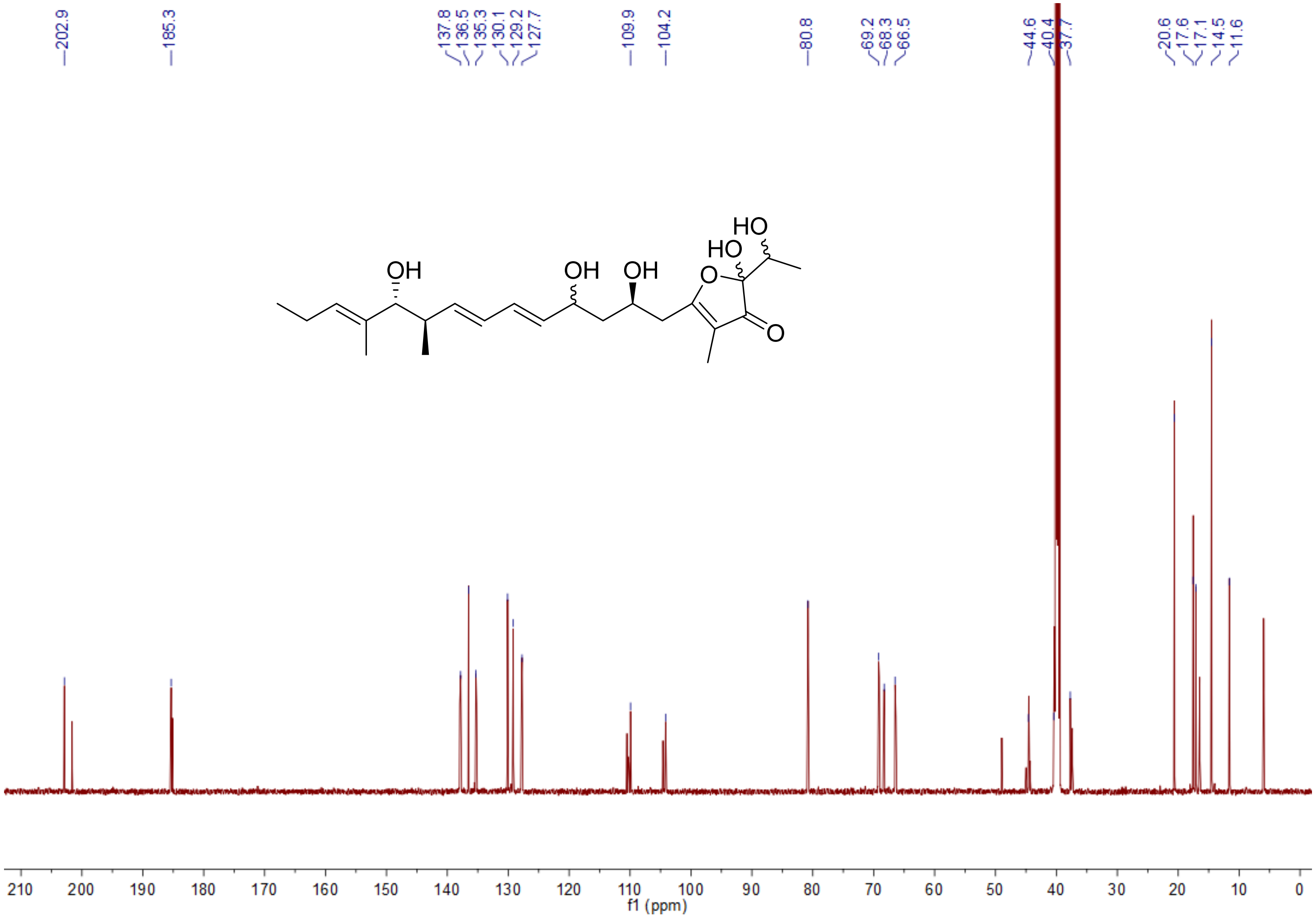

Figure S45. ^{13}C NMR (150 MHz, DMSO-d_6) spectrum of the new compound **5**

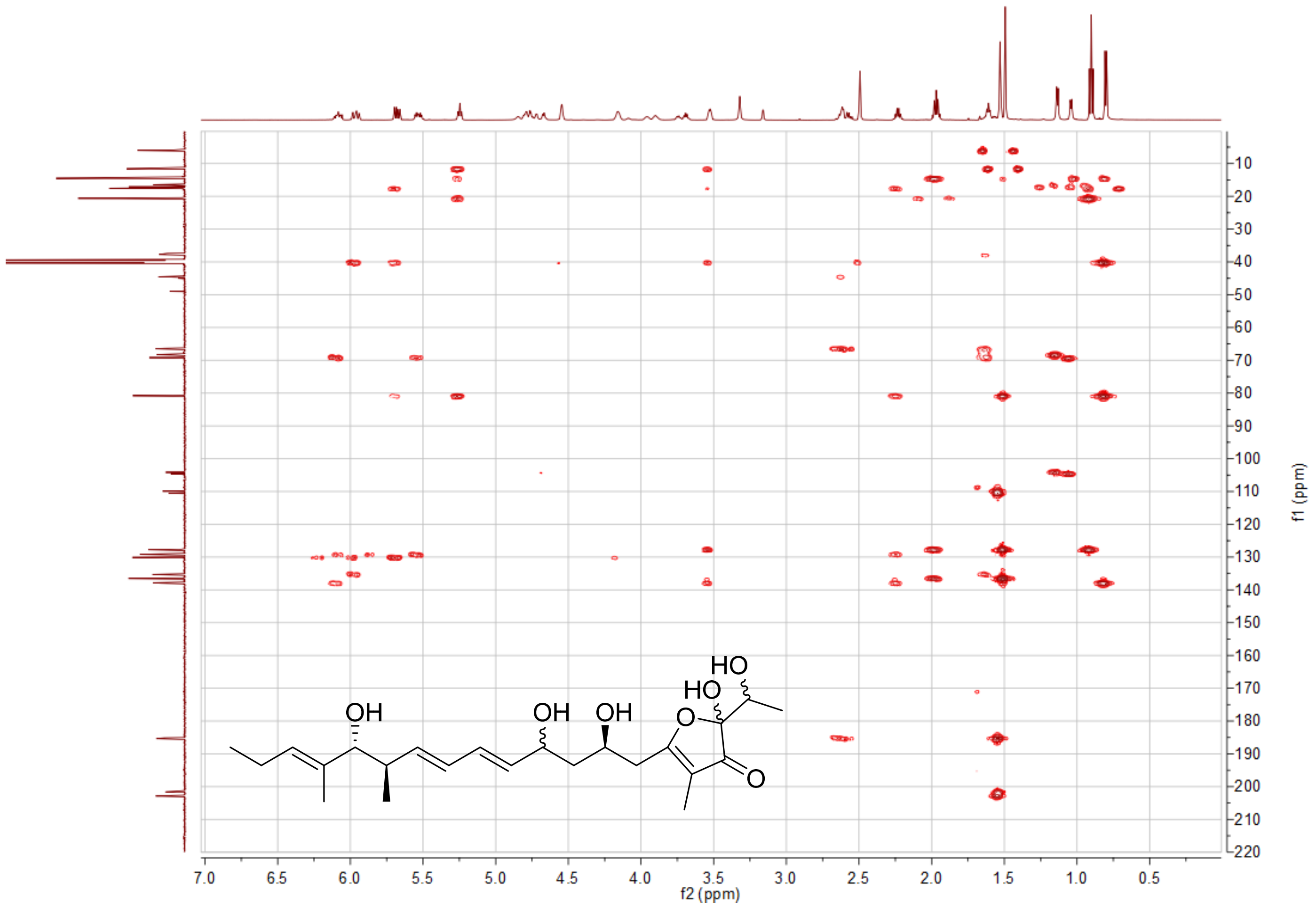

Figure S46. HSQC spectrum of the new compound **5**

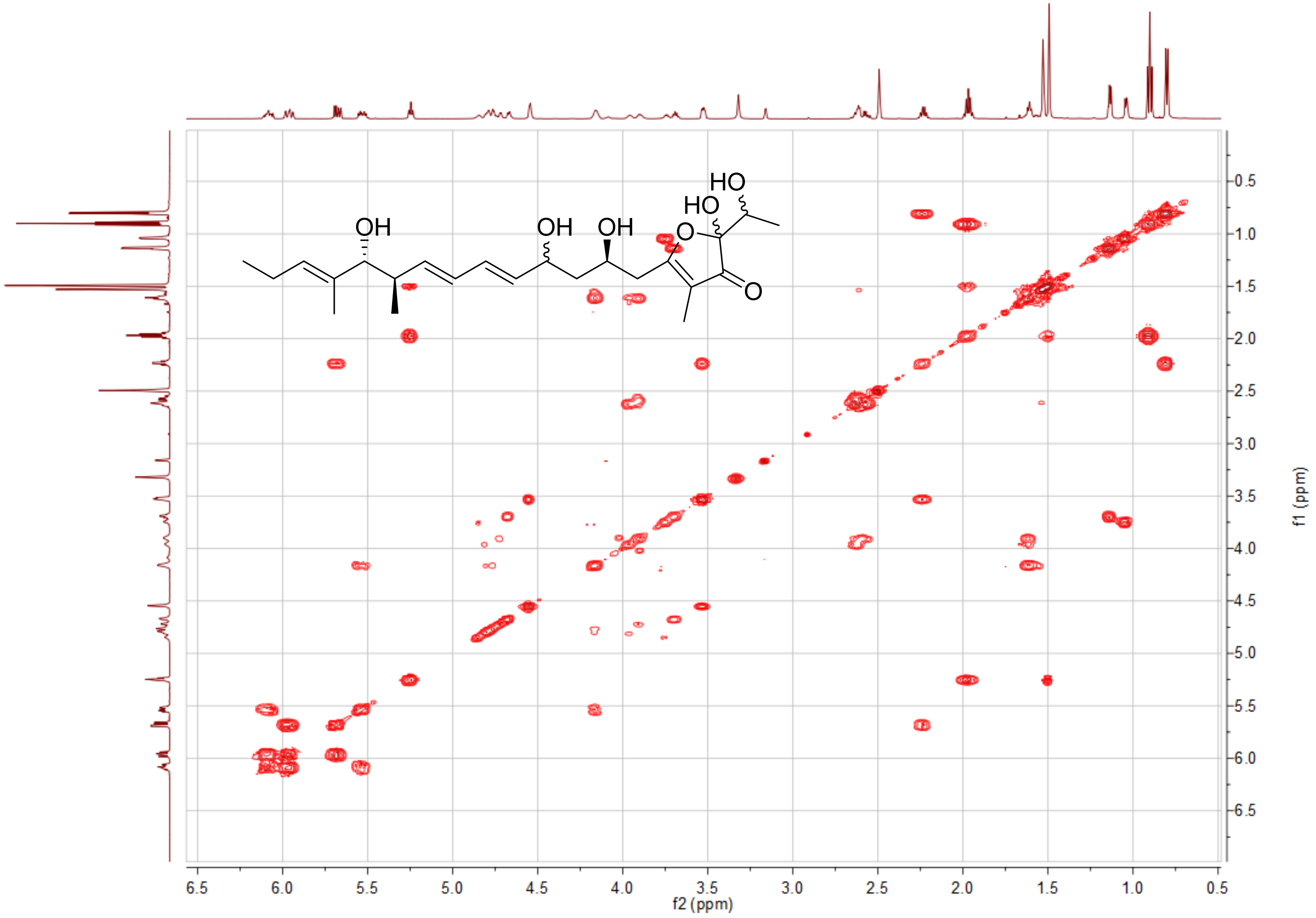

Figure S47. COSY spectrum (DMSO-d_6) of the new compound **5**

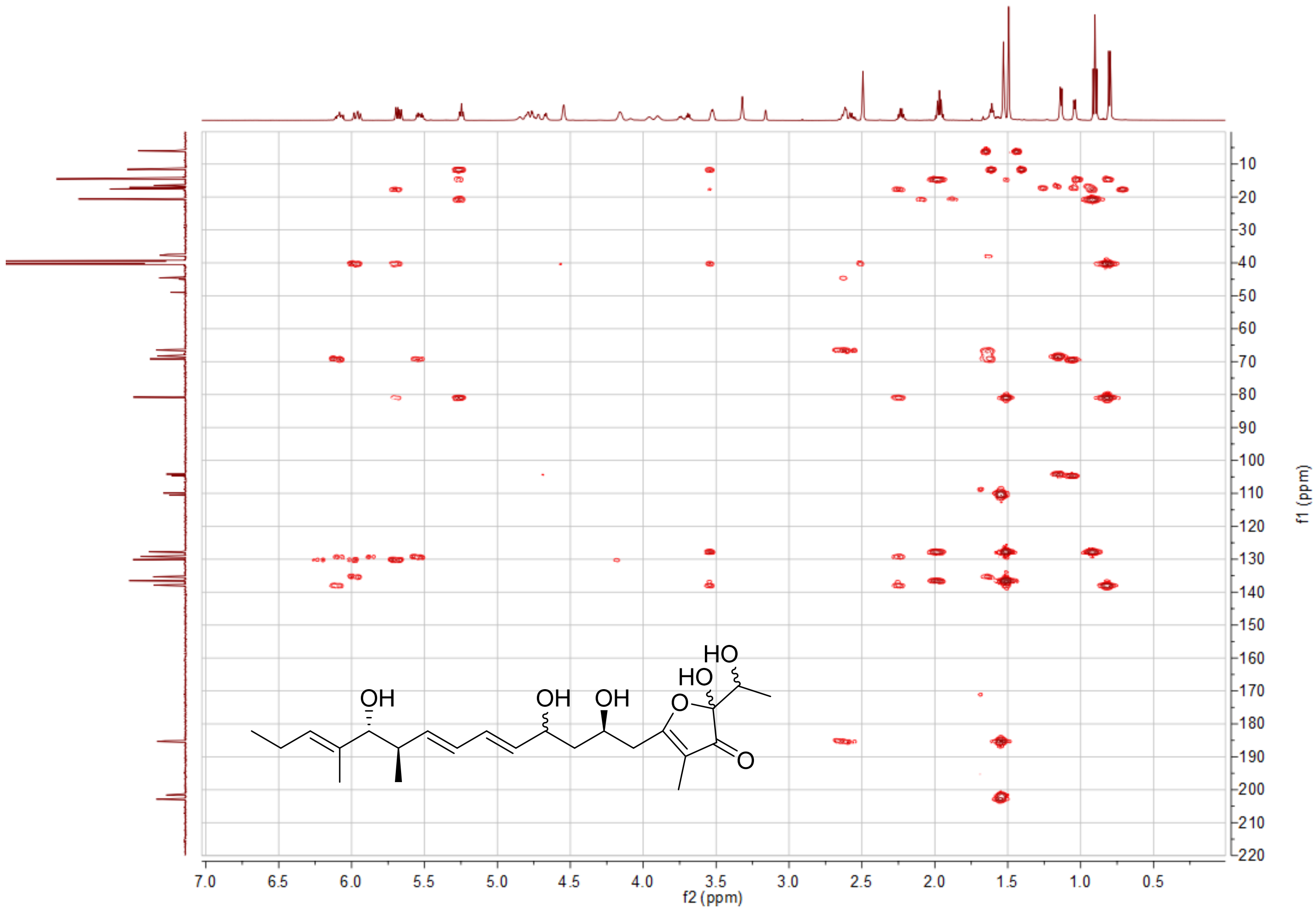

Figure S48. HMBC spectrum (DMSO-d_6) of the new compound **5**

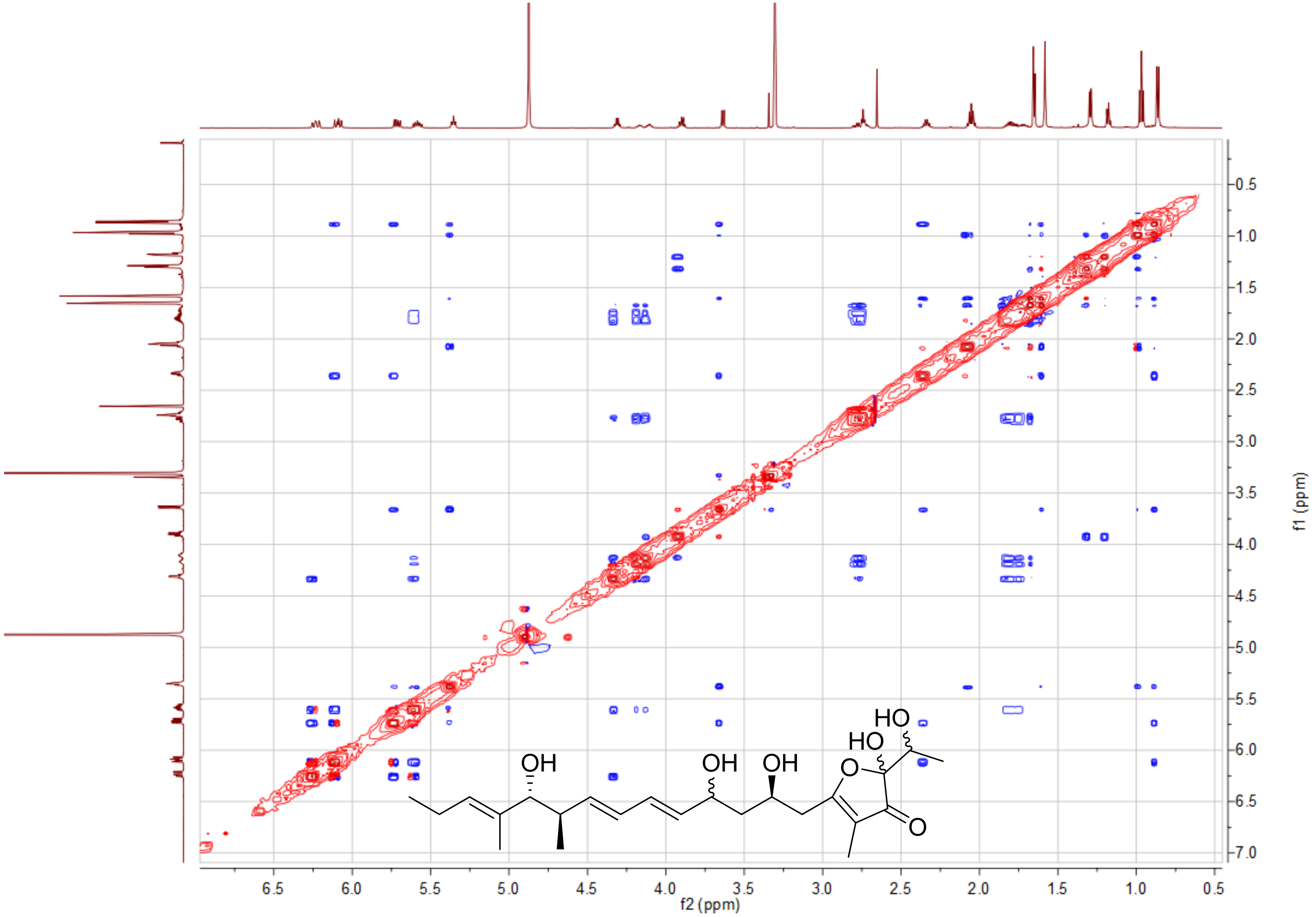

Figure S49. NOESY spectrum (CD_3OD) of the new compound **5**

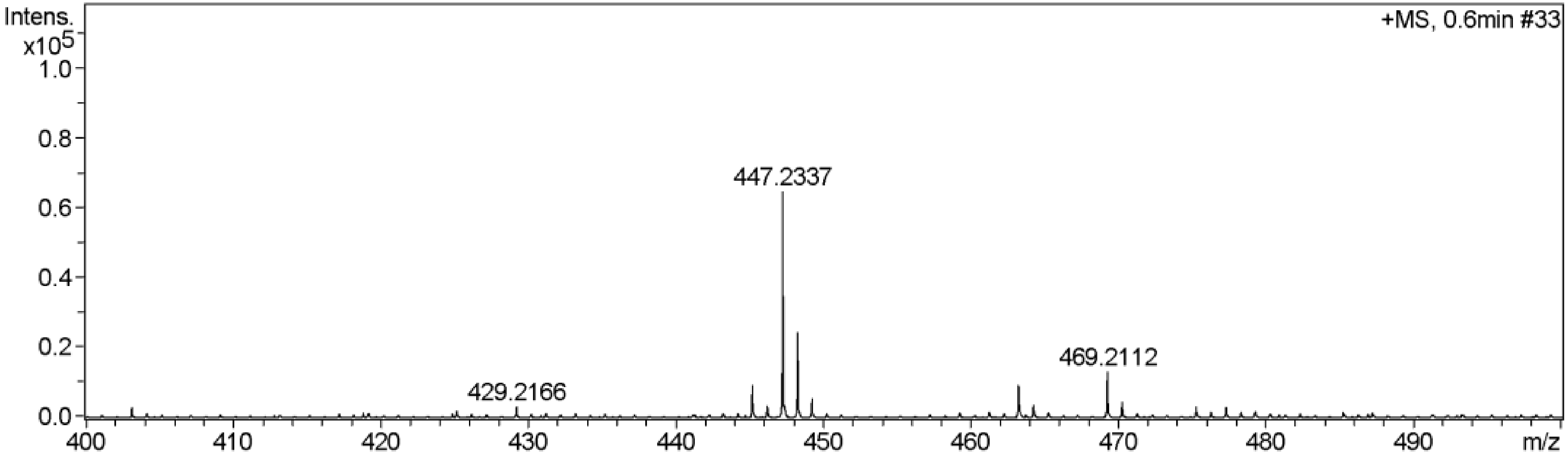

Figure S50. HRESI-MS spectrum of the new compound **6**

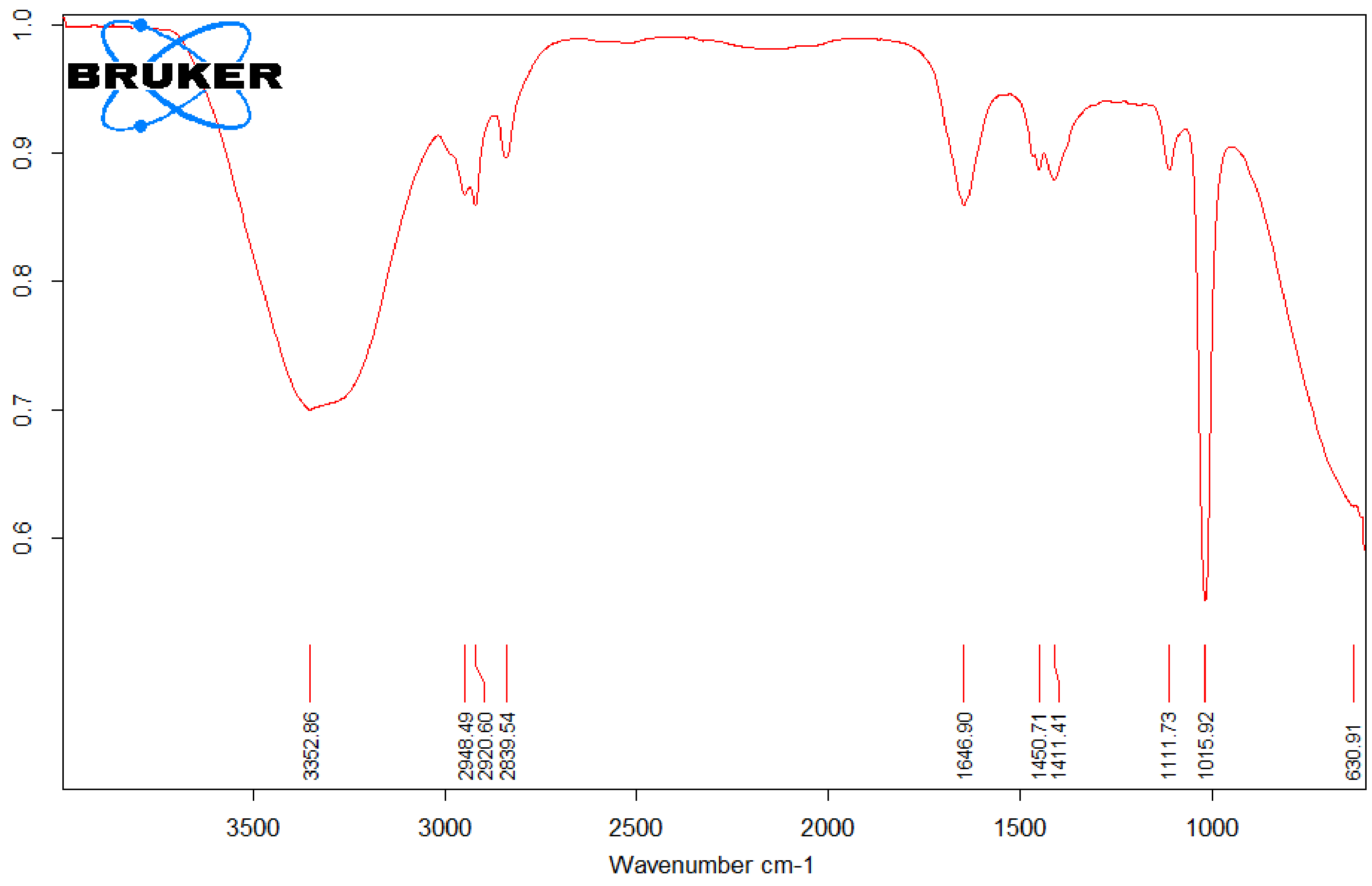

Figure S51. IR spectrum of the new compound **6**

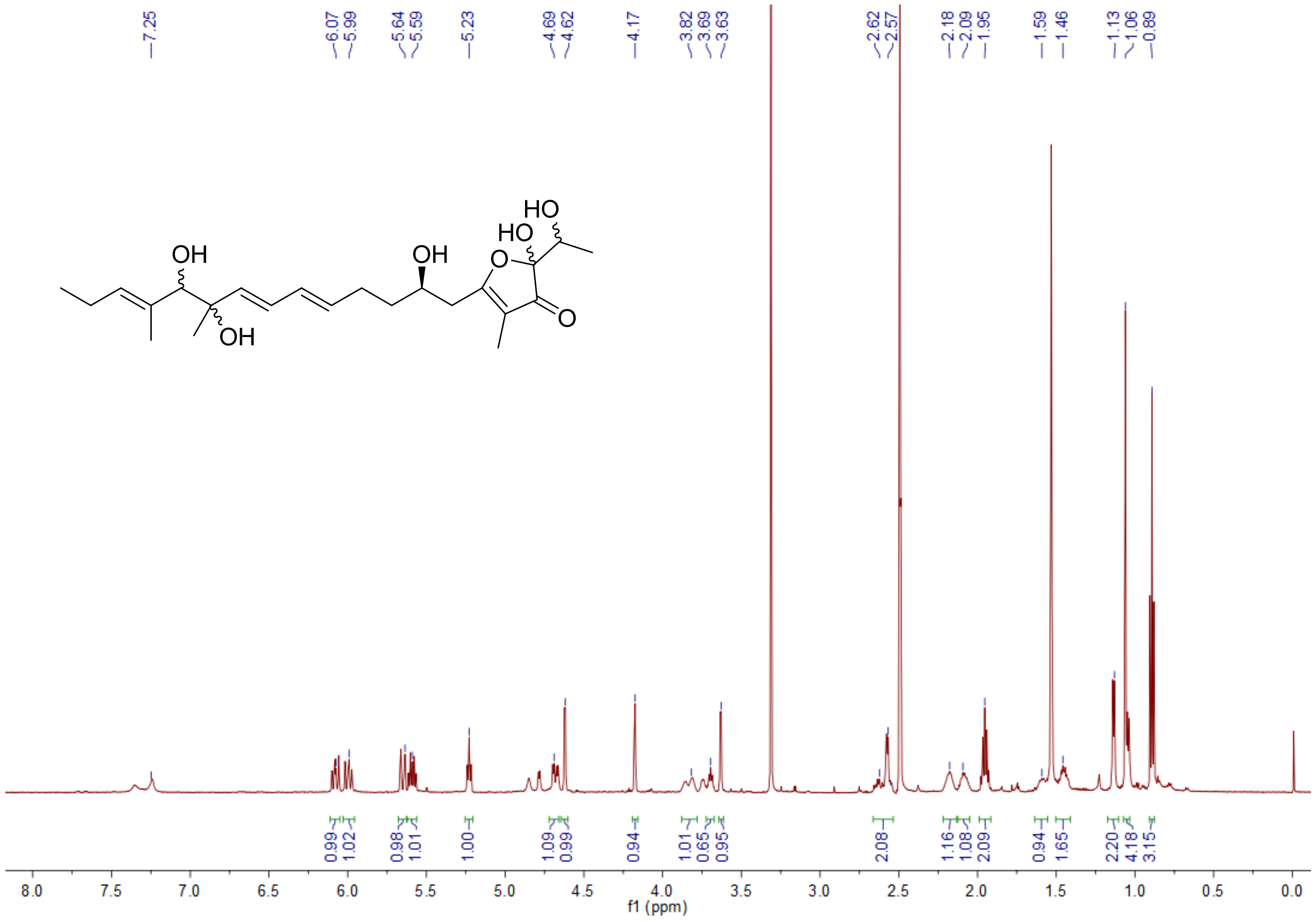

Figure S52. ^{1}H NMR (600 MHz, DMSO-d_6) spectrum of the new compound **6**

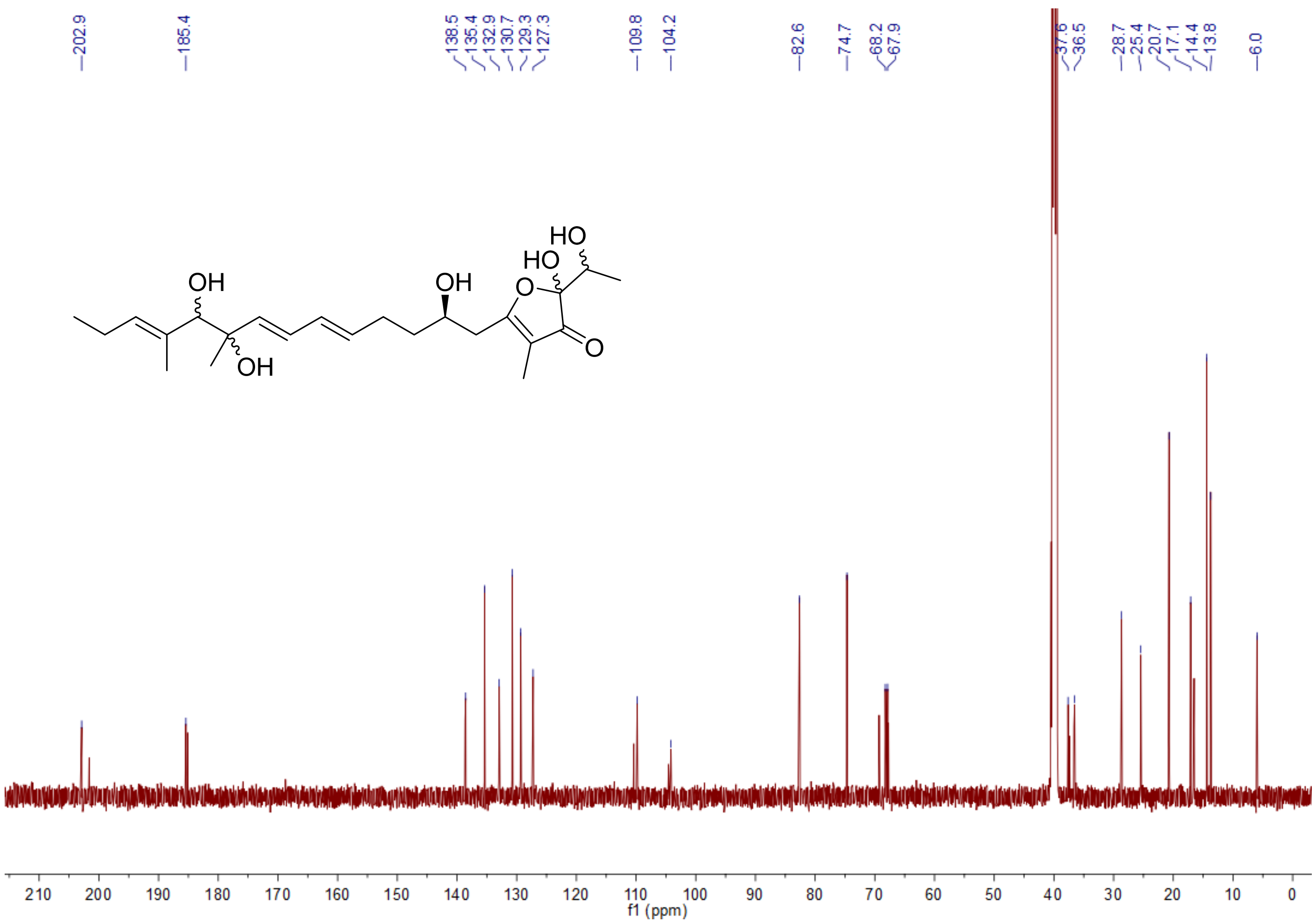

Figure S53. ^{13}C NMR (150 MHz, DMSO-d_6) spectrum of the new compound **6**

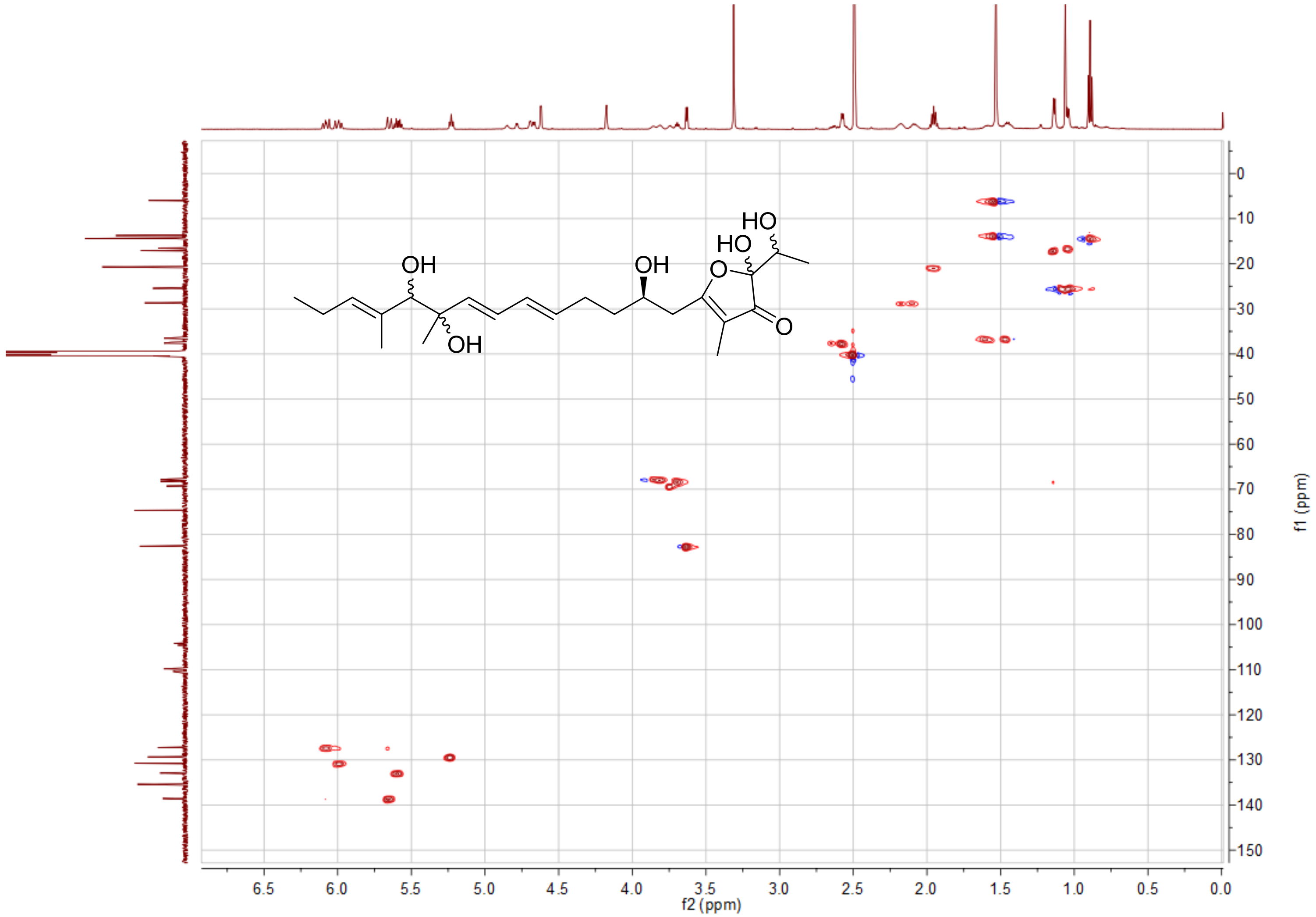

Figure S54. HSQC spectrum of the new compound **6**

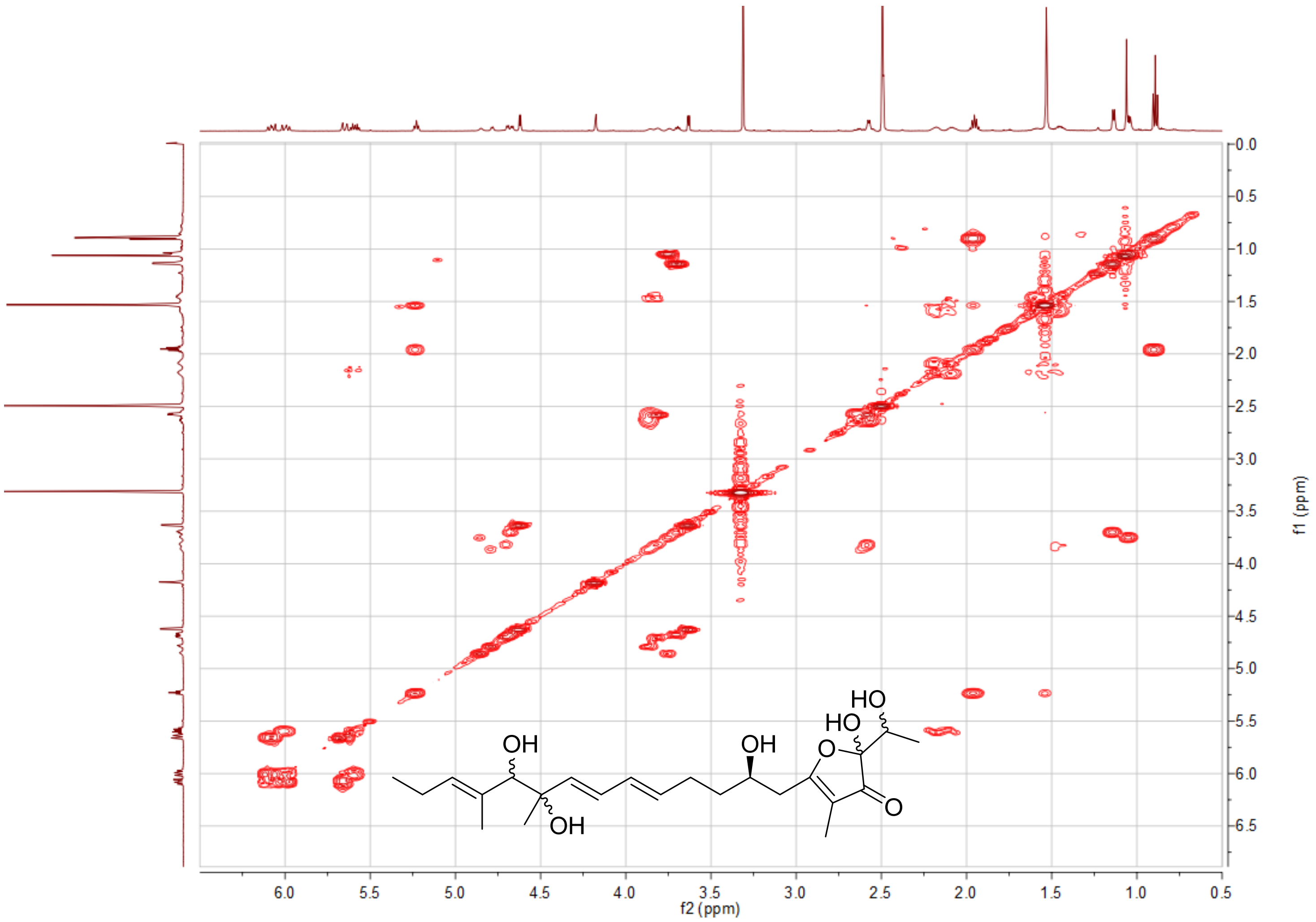

Figure S55. COSY spectrum (DMSO-d_6) of the new compound **6**

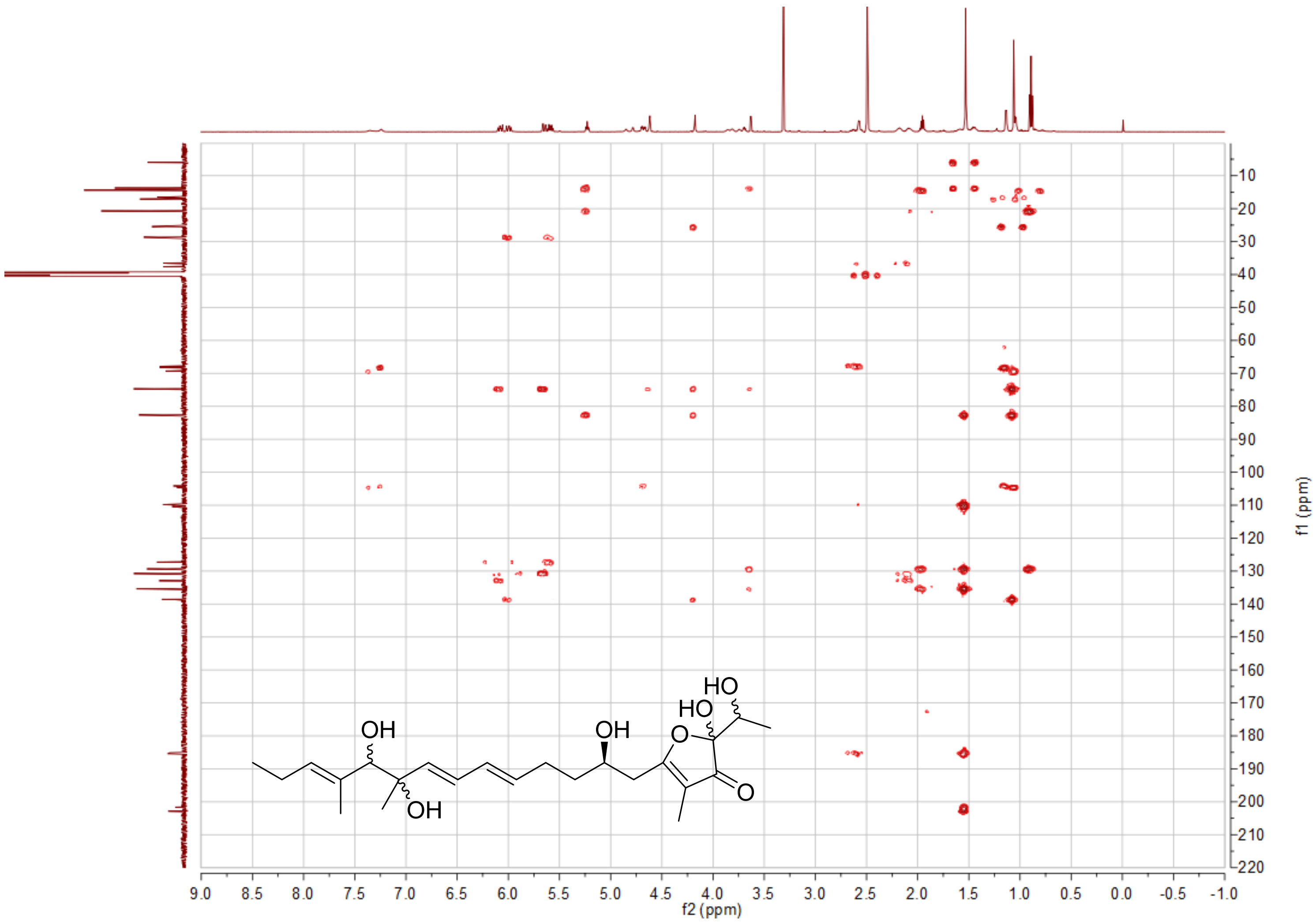

Figure S56. HMBC spectrum (DMSO-d_6) of the new compound **6**

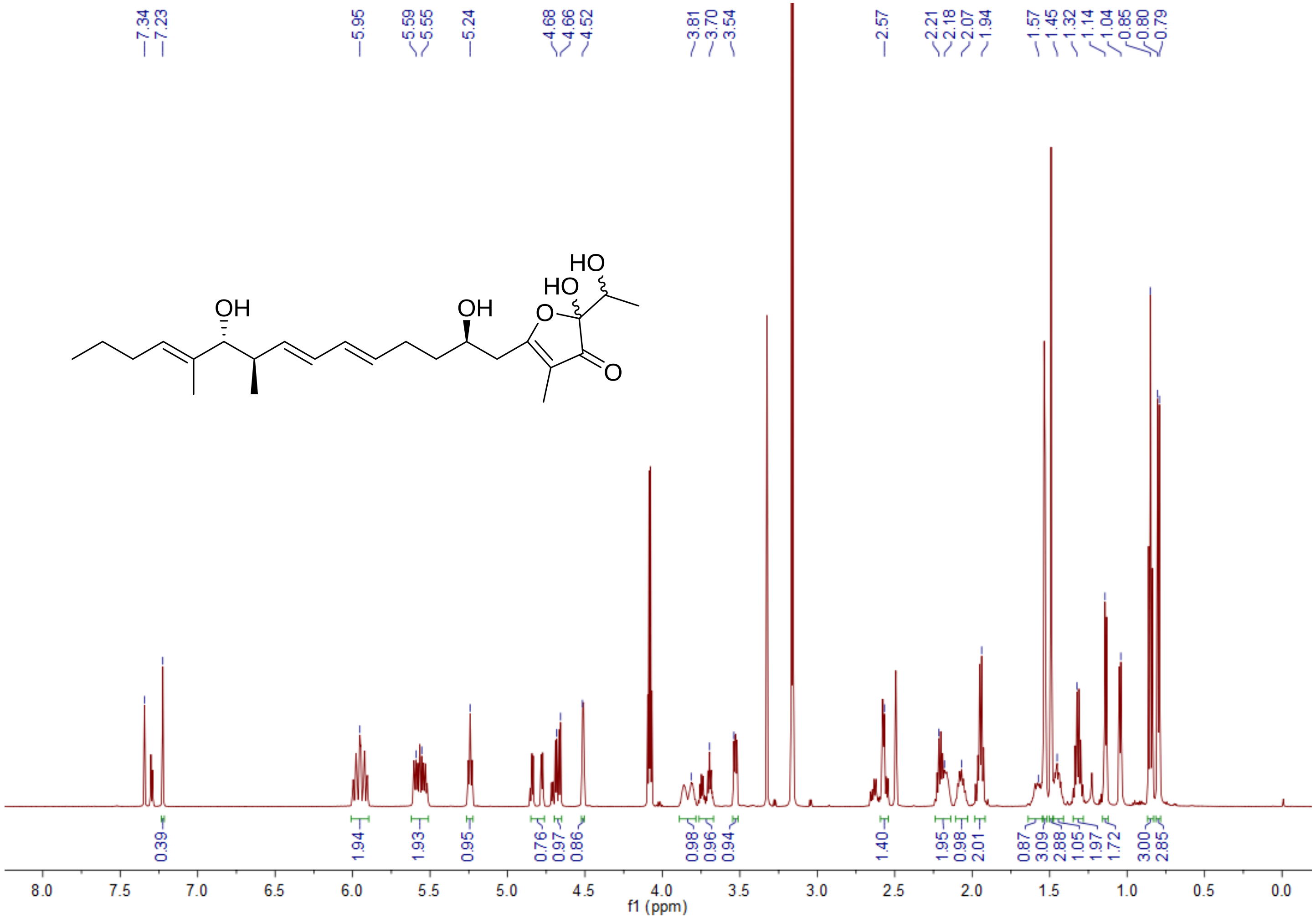

Figure S57. ^{1}H NMR (600 MHz, DMSO-d_6) spectrum of the compound **7**

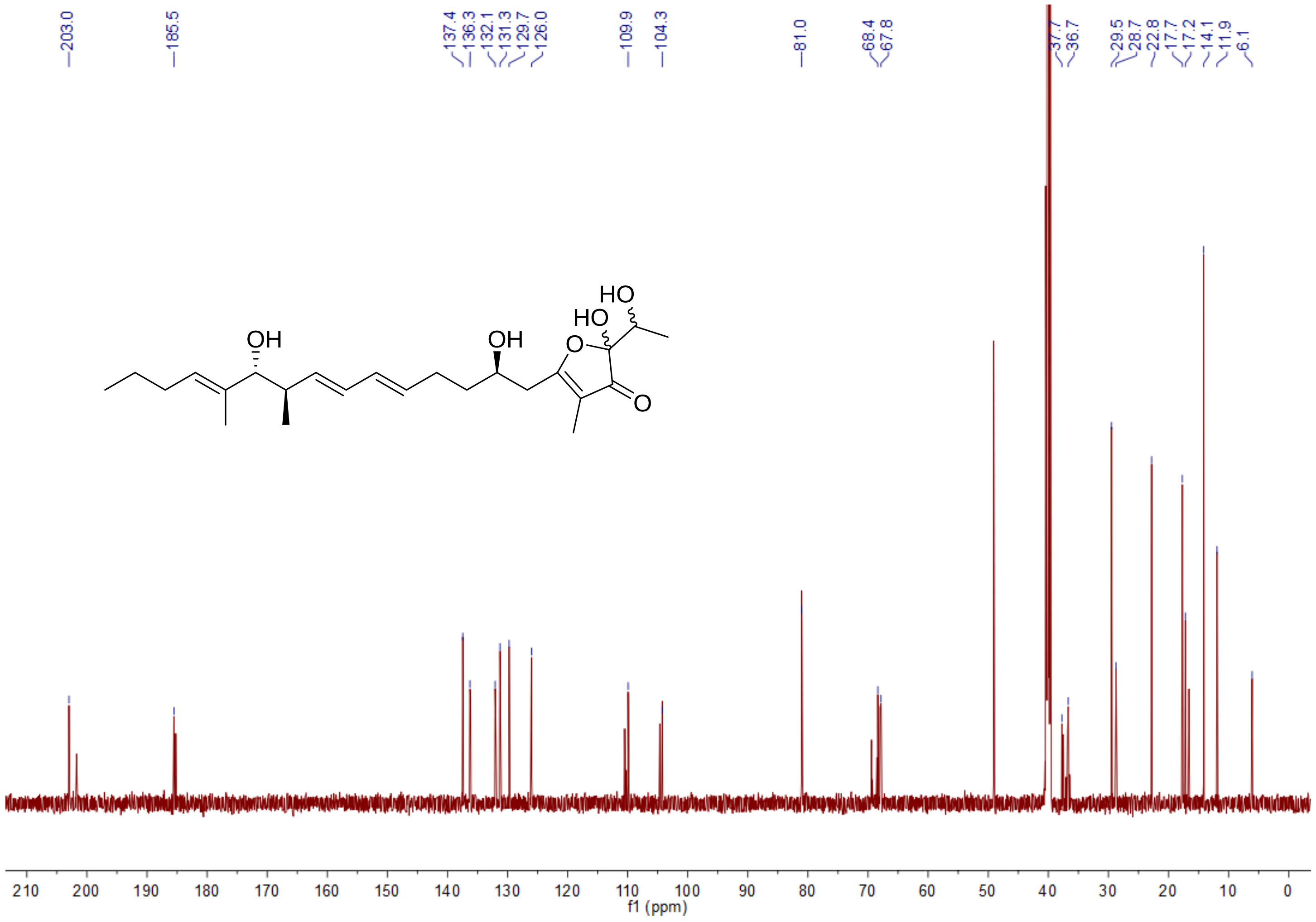

Figure S58. ^{13}C NMR (150 MHz, DMSO-d_6) spectrum of the compound **7**
